# False discovery rates of *qpAdm*-based screens for genetic admixture

**DOI:** 10.1101/2023.04.25.538339

**Published:** 2023-10-18

**Authors:** Eren Yüncü, Ulaş Işıldak, Matthew P. Williams, Christian D. Huber, Olga Flegontova, Leonid A. Vyazov, Piya Changmai, Pavel Flegontov

**Affiliations:** 1 Department of Biology and Ecology, Faculty of Science, University of Ostrava, Ostrava, Czechia; 2 Department of Biology, Eberly College of Science, Pennsylvania State University, PA, USA; 3 Institute of Parasitology, Biology Centre of the Czech Academy of Sciences, České Budějovice, Czechia; 4 Department of Human Evolutionary Biology, Harvard University, Cambridge, MA, USA

## Abstract

Although a broad range of methods exists for reconstructing population history from genome-wide single nucleotide polymorphism data, just a few methods gained popularity in archaeogenetics: principal component analysis (PCA); *ADMIXTURE*, an algorithm that models individuals as mixtures of multiple ancestral sources represented by actual or inferred populations; formal tests for admixture such as *f*_*3*_-statistics and *D/f*_*4*_-statistics; and *qpAdm*, a tool for fitting two-component and more complex admixture models to groups or individuals. Despite their popularity in archaeogenetics, which is explained by modest computational requirements and ability to analyze data of various types and qualities, protocols relying on *qpAdm* that screen numerous alternative models of varying complexity and find “fitting” models (often considering both estimated admixture proportions and *p*-values as a composite criterion of model fit) remain untested on complex simulated population histories in the form of admixture graphs of random topology. We analyzed genotype data extracted from such simulations and tested various types of high-throughput *qpAdm* protocols (“rotating” and “non-rotating”, with or without temporal stratification of target groups and proxy ancestry sources, and with or without a “model competition” step). We caution that high-throughput *qpAdm* protocols may be inappropriate for exploratory analyses in poorly studied regions/periods since their false discovery rates varied between 12% and 68% depending on the details of the protocol and on the amount and quality of simulated data (i.e., >12% of fitting two-way admixture models imply gene flows that were not simulated). We demonstrate that for reducing false discovery rates of *qpAdm* protocols to nearly 0% it is advisable to use large SNP sets with low missing data rates, the rotating *qpAdm* protocol with a strictly enforced rule that target groups do not pre-date their proxy sources, and an unsupervised *ADMIXTURE* analysis as a way to verify feasible *qpAdm* models. Our study has a number of limitations: for instance, these recommendations depend on the assumption that the underlying genetic history is a complex admixture graph and not a stepping-stone model.

## Introduction

Although a broad range of methods exists for reconstructing population history from genome-wide autosomal single nucleotide polymorphism (SNP) data, just a few methods became the cornerstone of archaeogenetic studies: principal component analysis (PCA) (Patterson et al. 2006); an unsupervised or supervised algorithm for admixture inference in individuals, *ADMIXTURE* (Alexander et al. 2009); formal tests for admixture such as *f*_*3*_-statistics (Patterson et al. 2012, Peter 2016, Soraggi and Wiuf 2019, Peter 2022) and *D-*statistics (Green et al. 2010, Durand et al. 2011); and a tool for fitting two-component and more complex admixture models to populations, *qpAdm* (Haak et al. 2015, Harney et al. 2021). The popularity of these methods is explained by their relatively modest computational requirements and versatility since they are capable of analysing unphased biallelic genotype data of various types (pseudo-haploid or diploid), generated using either targeted enrichment on a panel of sites or shotgun sequencing technologies, and low-coverage ancient genomes with high proportions of missing data (Harney et al. 2021). However, only a few studies were devoted to testing the performance of these diverse methods on simulated genetic data (Alexander et al. 2009, Harney et al. 2021, Lazaridis et al. 2017, Martin et al. 2014, McVean 2009, Moreno-Mayar et al. 2018b, Ning et al. 2020, Soraggi and Wiuf 2019), and realistically complex population histories remain virtually unexplored in this respect: both those approximated best by admixture graphs and isolation by distance (stepping-stone models, see Novembre and Stephens 2008, Duforet-Frebourg andSlatkin 2016).

In a typical archaeogenetic study published since the 2010s, PCA is used as a first line of analysis, providing an overview of population structure, and helping to propose hypotheses about migration and admixture. Distribution of individual genomes in two- or higher dimensional spaces of principal components (PCs) does not have unambiguous interpretation since even under ideal conditions (in the absence of missing data, batch artefacts, and selection signals) it is affected by both genetic drift and admixture (McVean 2009). Nevertheless, if context information such as geographical coordinates and dates for ancient individuals is available, PCA is routinely used for revealing “genetic clines” hypothesized as signs of admixture between originally isolated groups at the ends of such clines. However, multiple studies on simulated data showed that clinal PCA patterns do not necessarily indicate historical “admixture clines”: Lissajous curves such as horseshoe-shaped clines appear in PC spaces whenever smooth gradients of various origin exist in data (Podani and Miklós 2002, Diakonis et al. 2008, Novembre and Stephens 2008, Frichot et al. 2012, Duforet-Frebourg andSlatkin 2016, Estavoyer and Francois 2022). Thus, similar clinal patterns in PCAs based on genetic data can arise for many reasons: admixture between previously isolated groups (i.e., “admixture clines”), isolation by distance (Novembre and Stephens 2008), isolation by time in a panmictic population (Duforet-Frebourg andSlatkin 2016), and serial founder models without any gene flow (Estavoyer and Francois 2022). It was demonstrated that the distribution of individuals in the PC space depends on the expected coalescent time (McVean 2009), hence distinct demographic models with the same expected coalescence times are expected to have the same PCA projections. Additionally, imbalanced sampling across genetically divergent populations affects PCA results substantially (McVean 2009) and may obscure smooth Lissajous curves by artefactual sharp angles (Novembre and Stephens 2008). Considering this complexity, using further methods to correlate PCA results with other lines of evidence is necessary for studying migration history (Reich et al. 2008).

Formal tests for genetic admixture such as *f*_*3*_-statistics and *D/f*_*4*_-statistics are often used exactly for this purpose: to prove that a certain cline spotted in PC space is a result of migration and admixture of previously isolated ancestries and does not reflect isolation by distance or recurrent bottlenecks. *D*- and *f*_*4*_-statistics, which are identical except for the denominator and are not affected by genetic drift, test if an unrooted four-population tree fits the data (Reich et al. 2009, Green et al. 2010, Durand et al. 2011, Patterson et al. 2012). A statistically significant deviation of the statistic from 0 (estimated using jackknife or bootstrap resampling) means that either the assumed tree topology is wrong, or gene flow occurred between a pair of branches in the tree, assuming that recurrent mutations (Durand et al. 2011, Patterson et al. 2012) and SNP ascertainment bias (Flegontov et al. 2023) are absent. However, interpretation of these statistics is ambiguous since gene flow directionality remains unknown, and two pairs of branches can be responsible for a deviation of the statistic from 0 (Lipson 2020). Since gene flow may be mediated by ghost groups related only distantly to the sampled groups at the branch tips (Tricou et al. 2022), excluding one pair of branches due to geographical and temporal plausibility of gene flow is also difficult. And interpretation of deviations of *D*- and *f*_*4*_-statistics from 0 becomes hardly possible if both branch pairs are connected by detectable gene flows.

“Admixture” *f*_*3*_-statistics of the form *f*_*3*_(target; proxy source_*1*_, proxy source_*2*_) constitute another formal test for admixture (Patterson et al. 2012). Significant deviation of such a statistic from 0 in the negative direction (Z-score below −3) is considered proof of admixture since allele frequencies at most sites are intermediate in the target group between those in the proxy sources (Patterson et al. 2012). However, “admixture” *f*_*3*_-statistics are usually only applicable for detection of recent admixture events since they become positive when post-admixture genetic drift on the target lineage moves allele frequencies away from these intermediate values (Patterson et al. 2012, Peter 2016, Peter 2022).

Considering these complications, more sophisticated tests for genetic admixture are needed. The *qpAdm* method introduced by Haak *et al*. (2015) is based on matrices of *f*_*4*_-statistics and does not require detailed knowledge of population phylogeny beyond a few assumptions (Lazaridis et al. 2016, Harney et al. 2021). This method tests admixture models in the form of combinations of proxy ancestral groups (“sources” or “references”, Lazaridis et al. 2016) for a “target” (or “test”) population, given a genetically diverse set of “outgroup” populations, and infers ancestry proportions in the target group contributed by the lineages represented by the proxy sources (“outgroups” are often termed “right” populations for convenience since they are usually not outgroups in the phylogenetic sense, and they were termed “references” by Harney *et al*. 2021). See Box 1 for definitions of various *qpAdm*-related terms used in this study.

Eight years later we find *qpAdm*-based protocols routinely employed in large-scale screens of ancient human or animal populations for admixture (often between closely related sources) and used as formal tests for admixture (see Lazaridis et al. 2016, Skoglund et al. 2017, Harney et al. 2018, Mathieson et al. 2018, Antonio et al. 2019, Narasimhan et al. 2019, Fernandes et al. 2020, Marcus et al. 2020, Ning et al. 2020, Wang et al. 2020, Yang et al. 2020, Calhoff et al. 2021, Papac et al. 2021, Librado et al. 2021, Sirak et al. 2021, Wang et al. 2021, Yaka et al. 2021, Zhang et al. 2021, Allentoft et al. 2022, Bergström et al. 2022, Changmai et al. 2022a, Changmai et al. 2022b, Gnecchi-Ruscone et al. 2022, Lazaridis et al. 2022, Maróti et al. 2022, Oliveira et al. 2022, Patterson et al. 2022, Brielle et al. 2023, Lee et al. 2023, Taylor et al. 2023 for examples). *qpAdm* fits admixture models to a matrix of *f*_*4*_-statistics of the form *f*_*4*_(“left” group_*i*_, “left” group_*j*_; “right” group_*i*_, “right” group_*j*_), which in the absence of missing data at the group level can be reduced to a smaller matrix *f*_*4*_(target group, “left” group_*j*_; “right” group_*1*_, “right” group_*j*_), considering algebraic relationships between different *f*_*4*_-statistics (Peter 2016).

**Figure 1.**
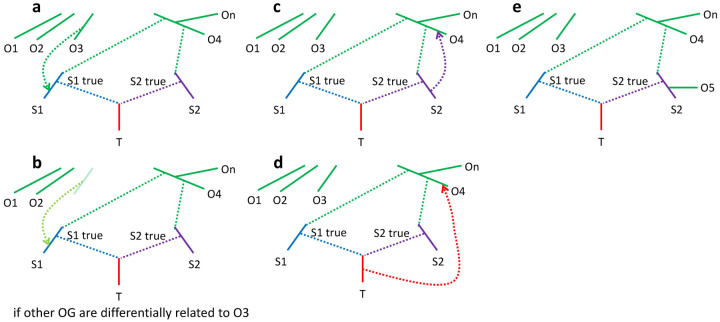
Admixture graphs showing an exhaustive list of assumption violations of the standard *qpAdm* protocol that may lead to rejection of the true simple model, and thus prompt the researcher to test overly complex models. (**a**) A gene flow from an outgroup O* to a proxy source after the divergence of the latter from the true source. (**b**) A gene flow from an unsampled source to a proxy source after the divergence of the latter from the true source. This case is problematic only if the outgroups are differentially related to the unsampled source. (**c**) A gene flow from a proxy source to an outgroup after the divergence of the former from the true source. (**d**) A gene flow from a target to an outgroup. (**e**) An outgroup is cladal with a proxy source.

A *qpAdm* protocol that has become the standard in archaeogenetics (Lazaridis et al. 2016) can be broken down into two parts: estimation of the number of gene flows connecting the “right” and “left” sets (this method was first published as a tool named “*qpWave*”, Reich et al. 2012) and inference of admixture proportions in a target group in the “left” set (Haak et al. 2015). *qpWave* tests for the number of distinct gene flows connecting the “right” and “left” population sets, does not infer directionality of gene flows, and does not identify recipients of gene flow in the “left” or “right” population sets. Notably, the standard *qpAdm* protocol relies on the following assumptions (Lazaridis et al. 2016, Harney et al. 2021): 1) there is at least one “right” population differentially related to the proxy sources; 2) proxy sources are strictly cladal with the true ancestral admixing sources (Fig. 1a, b), 3) there are no gene flows to populations located in the “right” set from the proxy source or target lineages either after the split of the proxy source from the true admixing source population or between the target population and the admixture event that gave rise to it (Fig. 1c–e). In the context of our study, true sources are unsampled populations that participated in a simulated admixture event (labelled as “S1 true” and “S2 true” in Fig. 1, see also Box 1). A more concise definition of these assumptions exists: there should be no genetic drift shared exclusively by proxy sources and “right” populations (but not by the target) and shared exclusively by the target and “right” populations (but not by proxy sources).

If the above assumptions are satisfied, it is safe to say that *qpWave/qpAdm* rejections of simpler models, and a failure to reject more complex models, are the result of a genuinely complex admixture history that connects the source and target populations rather than the false rejection of the simple model due to violations of any one of the assumptions described above. Most notably, violations of the second or third assumptions raise the probability of rejecting a simpler (true) model if enough data is available and prompt the researcher to test more complex (false) models (such as in Fig. 1 rejecting a two-source *qpAdm* model and exploring three-source models).

Harney *et al*. (2021) demonstrated on simulated data that, if the *qpAdm* assumptions are satisfied, it is highly favourable for statistical power of the method (for distinguishing between alternative proxy sources that are unequally distant genetically from the true ancestry source) to move at least some alternative proxy sources between the “left” and “right” sets. In other words, having “on the right” populations that do not violate the topological assumptions of *qpAdm*, but are closely related to proxy sources on the “left”, increases the statistical power greatly (see also Ning et al. 2020 for another demonstration of this on simple simulated histories).

This new type of *qpAdm* protocols, termed “rotating” protocol, has been adopted in archaeogenetics widely (see, e.g., Skoglund et al. 2017, Harney et al. 2019, Narasimhan et al. 2019, Olalde et al. 2019, Calhoff et al. 2021, Fernandes et al. 2021, Librado et al. 2021, Allentoft et al. 2022, Bergström et al. 2022, Lazaridis et al. 2022, Oliveira et al. 2022, Taylor et al. 2023). The most extreme version of the “rotating” protocol simply divides a set of reference populations into all possible combinations of “right” and “proxy source” subsets of certain sizes and rotates these combinations through successive *qpAdm* analyses. Additional constraints can be placed on the rotating combinations such as restricting a set of groups (usually highly divergent from the target) to remain in the “right” set in all models. When evaluating the totality of multiple *qpAdm* tests, the simplest feasible models (e.g., one-way, i.e., unadmixed) are favoured, and increasingly complex models are explored upon the rejection of simpler models. Model rejection for the simplest models is made according to a chosen *p*-value threshold such that *qpAdm* models are considered feasible or “fitting” the data when the *p*-value is above such a threshold (Skoglund et al. 2017, Harney et al. 2018, Narasimhan et al. 2019, Olalde et al. 2019, Yang et al. 2020, Calhoff et al. 2021, Fernandes et al. 2021, Librado et al. 2021, Zhang et al. 2021, Allentoft et al. 2022, Bergström et al. 2022, Lazaridis et al. 2022, Oliveira et al. 2022, Taylor et al. 2023). As an additional criterion of a fitting model, all inferred admixture proportions (Harney et al. 2018, Olalde et al. 2019, Yang et al. 2020, Zhang et al. 2021, Allentoft et al. 2022, Lazaridis et al. 2022, Oliveira et al. 2022), or proportions ± 2 standard errors (Narasimhan et al. 2019), may be required to lie between 0 and 1. It is important to remember that the statistical significance of the *qpAdm/qpWave* test is, strictly speaking, a function of model rejection, and thus the failure to reject a model may have underlying causes other than approximating the true situation well enough (such as lack of statistical power or a lack of suitable “right” groups that capture the divergent ancestry sources amongst the “left” set of populations).

A less exhaustive version of the rotating *qpAdm* protocol, termed “model competition” (e.g., Narasimhan et al. 2019, Fernandes et al. 2020, Calhoff et al. 2021, Sirak et al. 2021, Zhang et al. 2021, Maróti et al. 2022, Brielle et al. 2023, Lee et al. 2023), is used even more widely than the basic rotating protocol. It involves an initial (standard non-rotating) *qpAdm* analysis on a number of source populations (see Box 1). Upon identifying a list of plausible sources for a target, the algorithm re-tests feasible models for this target rotating these plausible sources between the “left” and “right” sets with the expectation of improving the power to reject models including proxy sources that are genetically distant from the true sources.

The rotating *qpAdm* protocol and model competition are increasingly used as central methods for testing admixture hypotheses proposed after inspecting distributions of individuals in PC spaces, outcomes of *ADMIXTURE* analyses, and *f/D*-statistics indicative of an admixture graph rather than a simple tree relationship. Yet, the only study reporting detailed testing of *qpAdm* on simulated data (Harney et al. 2021) was performed in extremely favorable conditions: the simulated graph included just two non-nested admixture events; the sources for the principal target group diverged about 1,200 generations ago (almost 35,000 years ago in the case of humans); the proxy sources were strictly cladal with the actual ancestral groups for the target; several groups differentially related to these ancestry sources were available; the simulated data were free of ascertainment bias since sites were sampled in a random way; one million sites were used for most analyses; and only 50/50% simulated admixture proportions were tested for some analyses. This study confirmed that the method behaves as expected under these ideal conditions and offered some important guidelines on the choice and number of “right” populations for optimal specificity of the method and on model comparison strategies, and also showed that the results are robust to the presence of missing data, imbalanced group sizes, ancient DNA damage, and to a particular type of SNP ascertainment: selecting sites heterozygous in one individual from a certain population (Patterson et al. 2012). Among challenging historical scenarios, only multifurcation and a one-dimensional stepping-stone model with uniform and continuous gene flow among several groups was explored, and it was concluded that *qpAdm* is not applicable in this case (Harney et al. 2021). Meanwhile, false discovery rate (FDR) or false positive rate (FPR) of the method and violations of the topological assumptions of *qpAdm* (Fig. 1) remained virtually unexplored. Thus, the method was proven to work and fail in polar (either extremely favourable or extremely unfavourable) conditions. But what happens in intermediate cases where arguably most of the history of humans and other mammals fits: a history that is not a nearly perfect tree, but that, on the other hand, cannot be approximated solely as isolation by distance?

We are concerned that *qpAdm* performance may be compromised by the fact that the topological assumptions of the method are hard to verify in practice, especially the assumption about cladality of proxy and true ancestry sources (i.e., no gene flow to the proxy source population after its split from the true admixing source population). To explore this problem, we analyse simulated population histories in the form of complex random admixture graphs and test various types of *qpAdm* protocols common in the literature: rotating and non-rotating, with or without temporal stratification of target groups and proxy ancestry sources, and with or without a model competition step. We also reproduced other aspects of a typical archaeogenetic study on simulated data: we combined various *qpAdm* protocols with PCA and an unsupervised *ADMIXTURE* analysis to explore FDR of complex admixture screening pipelines.

## Results

### An overview of published rotating and model competition qpAdm protocols

First, we outline two published rotating *qpAdm* protocols (Narasimhan et al. 2019, Lazaridis et al. 2022) that are typical for this class of protocols (see further examples in Skoglund et al. 2017, Harney et al. 2019, Olalde et al. 2019, Calhoff et al. 2021, Fernandes et al. 2021, Librado et al. 2021, Allentoft et al. 2022, Bergström et al. 2022, Oliveira et al. 2022, Taylor et al. 2023). Lazaridis *et al*. relied on the following set of 15 reference populations: 1) Mbuti (present-day Africans); 2) a Palaeolithic group from the Caucasus (CHG, Caucasian hunter-gatherers); 3) East European Mesolithic (EHG, East European hunter-gatherers); 4) Ganj Dareh (a Neolithic group from Iran); 5) Natufians (an Epipalaeolithic group from Israel); 6) a Pre-pottery Neolithic (PPN) group from the Levant; 7) Taforalt (an Epipalaeolithic group from Morocco); 8) Neolithic Mesopotamia; 9) Afontova Gora 3 (an individual from Late Upper Palaeolithic Siberia); 10) Mal’ta 1 (an individual from Late Upper Palaeolithic Siberia); 11) a Mesolithic group from the Iron Gates region (Serbia); 12) Boncuklu (a Pre-pottery Neolithic group from Central Turkey); 13) Barcın (a Neolithic group from Western Turkey); 14) Pınarbaşı (an Epipalaeolithic individual from Turkey); and 15) Mesolithic and Palaeolithic individuals from Western Europe (WHG, West European hunter-gatherers).

**Figure 2.**
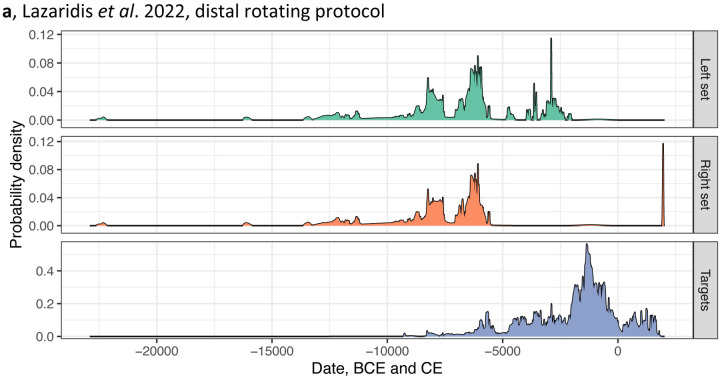

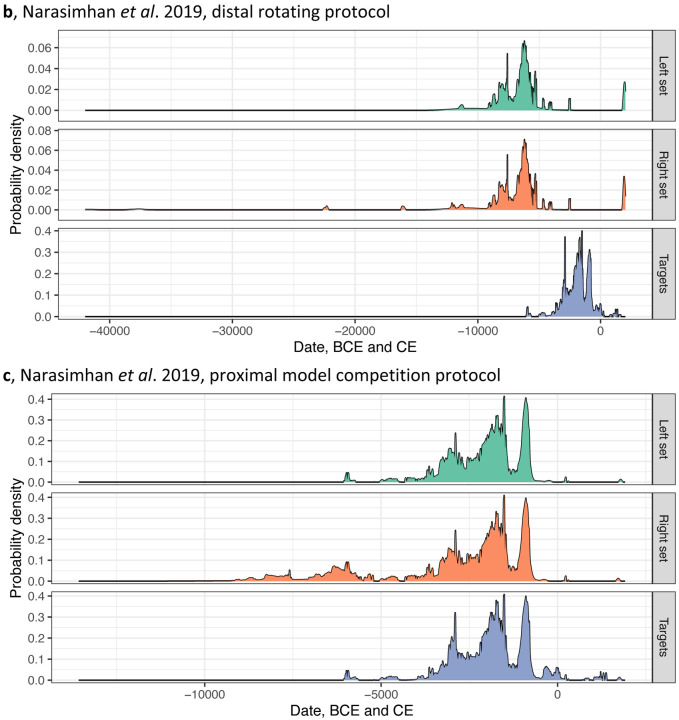
Distributions of radiocarbon and calendar dates for populations sets analyzed with the distal rotating *qpAdm* protocols by Lazaridis *et al*. 2022 (**a**) and Narasimhan *et al*. 2019 (**b**), and with the proximal model competition protocol from Narasimhan *et al*. 2019 (**c**). Probability density curves are shown for three sets of groups: 1) those appearing in the “right” set in at least one *qpAdm* model; 2) those appearing in the “left” set in at least one *qpAdm* model; 3) target groups. Targets in the former study were composed of large clusters of West Eurasian individuals, some of them dating back to the Palaeolithic (Lazaridis et al. 2022). For that reason, the date distribution for targets in panel **a** is very wide.

This reference set was divided into all possible “right” and proxy source subsets, except for the African group (Mbuti) which stayed in the “right” set in all models. Three Chalcolithic groups from Iran and an Early Bronze Age group from Russia (Yamnaya) were considered as proxy sources only and not rotated to the “right” set, and various clusters of Chalcolithic and Bronze Age individuals (most of them dated between ca. 5000 and 1000 years BCE) from the Balkans, Anatolia, Levant, Caucasus, Mesopotamia, and Iran were target groups for the *qpAdm* analyses. Thus, this protocol can be classified as a distal rotating protocol (Box 1) since most (but not all) targets do not pre-date the proxy sources (Fig. 2a). For each target, progressively more complex admixture models were tested, including from one to five proxy sources, and in most cases only the simplest feasible models were interpreted. Model feasibility criteria were as follows: estimated admixture proportions between 0 and 1, and *p-*value > 0.01. Among alternative models for the same target, those having a higher *p*-value were considered fitting the data better (Lazaridis et al. 2022).

As shown in Fig. 2a, in this analytical setup there is a large temporal overlap between “left” groups (targets and, on average, earlier proxy sources) and “right” groups. For instance, such a divergent and ancient group as the Mal’ta 1 individual from the vicinity of Lake Baikal (dated to ca. 24,000 years before present, yBP, Raghavan et al. 2014) appeared “on the left” in some *qpAdm* models. Thus, “left-to-right” gene flows (that may lead to erroneous conclusions from a *qpAdm* analysis, see Fig. 1) are expected to be common in the analytical setup used by Lazaridis *et al*.

Narasimhan *et al*. (2019) used both proximal and distal *qpAdm* protocols (Box 1). The distal rotating protocol relied on the following set of 16 reference populations: 1) Mota (a 4500-years-old individual from Ethiopia); 2) Ust’-Ishim (an Upper Palaeolithic individual from West Siberia); 3) Tianyuan (an Upper Palaeolithic individual from Northeast China); 4) Late Upper Palaeolithic individuals from Siberia (Afontova Gora 3 and Mal’ta 1, collectively labelled “ANE” or “Ancient North Eurasians”); 5) a Late Upper Palaeolithic individual from Italy (Villabruna); 6) Natufians (an Epipalaeolithic group from Raqefet, Israel); 7) a Mesolithic individual from Iran (Belt Cave); 8) present-day Andamanese; 9) East European Mesolithic individuals (EEHG, East European hunter-gatherers); 10) West Siberian Mesolithic (WSHG, West Siberian hunter-gatherers); 11) a Pre-pottery Neolithic (PPN) group from the Levant; 12) a Mesolithic group from the Iron Gates region (WEHG, West European hunter-gatherers); 13) Anatolian Neolithic individuals; 14) Ganj Dareh (a Neolithic group from Iran); 15) an Early Neolithic group from the Baikal region (ESHG, East Siberian hunter-gatherers); and 16) present-day Han Chinese. This reference set was split into all possible “right” and proxy source subsets, except for the Upper Palaeolithic individuals/groups (Ust’-Ishim, Tianyuan, ANE, Villabruna) who stayed in the “right” set in all models. Diverse groups from Iran, Pakistan, Central Asia, and the Russian steppe zone (dated from the Chalcolithic to the historical period) were used as targets for the *qpAdm* protocol. For each target, generally post-dating the proxy sources (Fig. 2b), progressively more complex admixture models were tested, from one- to five-way mixture models, and in most cases only the simplest feasible models were interpreted. Model feasibility criteria were as follows: estimated admixture proportions ± 2 standard errors are between 0 and 1, and *p*-value > 0.01 (Narasimhan et al. 2019).

In summary, this *qpAdm* protocol rotated a diverse set of groups between the “right” and “left” sets: from present-day to Mesolithic groups older than 10,000 years, and from Africans to South and East Asians (see date distributions in Fig. 2b). Another *qpAdm* protocol used by Narasimhan *et al*., termed “proximal” protocol (Box 1), relied on a smaller fixed set of groups that were kept always “on the right”: 1) Mota (a 4500-years-old individual from Ethiopia); 2) East European Mesolithic individuals (EEHG, East European hunter-gatherers); 3) West Siberian Mesolithic (WSHG, West Siberian hunter-gatherers); 4) a Pre-pottery Neolithic (PPN) group from the Levant; 5) a Mesolithic group from the Iron Gates region (WEHG, West European hunter-gatherers); 6) Anatolian Neolithic individuals; 7) Ganj Dareh (a Neolithic group from Iran); 8) an Early Neolithic group from the Baikal region (ESHG, East Siberian hunter-gatherers). Thirty-one diverse Neolithic, Chalcolithic and Bronze Age groups from Eurasia were originally used as proxy sources in one- to three-way models, but if several feasible models were found for the target, the proxy sources from those models were moved one by one from the “left” to the “right” sets (i.e., model competition was performed). The set of targets and the model feasibility criteria matched those for the “distal” protocol. At the model competition step, groups very close in space and time appeared on both sides of the “left” - “right” and proxy source - target divides (Fig. 2c), making “left-to-right” gene flows (Fig. 1) highly likely. Of 45 target groups, 23 groups were also used as rotated proxy sources. For this reason, we interpreted the Narasimhan *et al*. proximal model competition protocol as follows (Box 1): its first step is a non-rotating *qpAdm* protocol with temporal stratification of “right” and “left” sets, but with no (or very limited) temporal stratification of targets and proxy sources; and the second step is a model competition protocol with no (or very limited, see Fig. 2c) temporal stratification of targets and proxy sources. We note that any such interpretation is an approximation that captures most important features of a published protocol and omits some details.

Since the sets of groups that are split into the “left” and “right” subsets in the protocols summarized above are very diverse chronologically and genetically, and since there are substantial overlaps in dates between the “left” and “right” subsets (Fig. 2), we argue that this approach is essentially similar to taking an admixture graph connecting populations sampled at widely different times in history, with divergence dates ranging from the Palaeolithic (up to ca. 87,000 yBP in our simulations) to the “present” in the context of the dataset, and *randomly* splitting this graph into “left” and “right” population sets.

### Testing qpAdm performance on complex simulation histories

**Figure 3.**
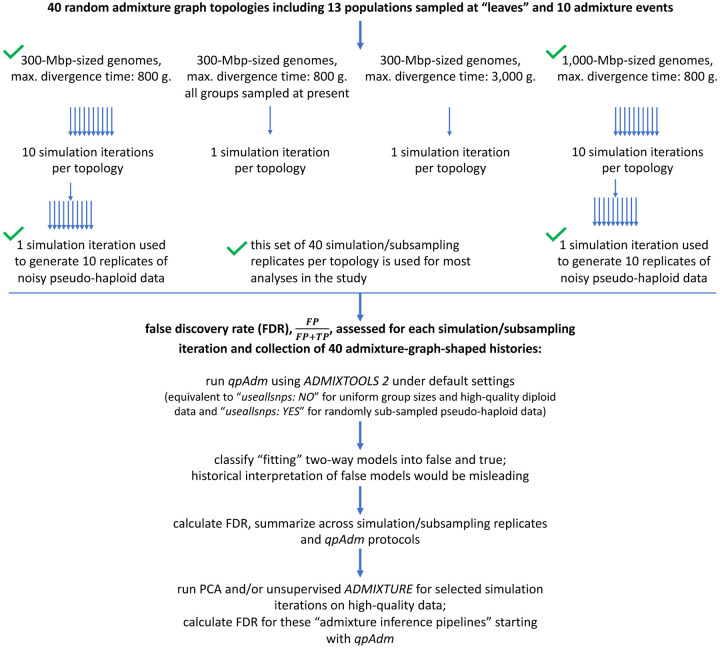
A diagram illustrating our analytical workflow.

Below we explore performance on simulated data (mainly FDR) of *qpAdm* protocols representing the spectrum of protocols used in the literature (for an overview of our workflow see Fig. 3). The most extreme example is a protocol where all groups are rotated and all are treated alternatively as outgroups, targets, and proxy sources, i.e., there is no temporal stratification between the latter categories. We term this protocol “proximal rotating” (see Tables S1 and S2). Although such an extreme situation is, to our knowledge, rare among published *qpAdm* protocols (see Calhoff et al. 2021, Oliveira et al. 2022; in the latter study the rotating *qpAdm* strategy was used to model groups dated to 450–2600 yBP as mixtures of present-day groups), we use it to illustrate the effects of poor temporal stratification of targets and proxy sources in rotating protocols (Fig. 2). Models with targets pre-dating proxy sources are encountered in high-throughput *qpAdm* screens, but do not constitute a majority of models (Narasimhan et al. 2019, Librado et al. 2021, Allentoft et al. 2022, Bergström et al. 2022, Lazaridis et al. 2022, Taylor et al. 2023). We also explore FDR of the proximal non-rotating (Harney et al. 2018, van de Loosdrecht et al. 2018, Narasimhan et al. 2019, Prendergast et al. 2019, Wang et al. 2020, Calhoff et al. 2021, Wang et al. 2021, Zhang et al. 2021, Changmai et al. 2022a, Changmai et al. 2022b, Maróti et al. 2022, Brielle et al. 2023, Lee et al. 2023), distal rotating (Narasimhan et al. 2019, Librado et al. 2021, Allentoft et al. 2022, Bergström et al. 2022, Lazaridis et al. 2022, Taylor et al. 2023), and distal non-rotating protocols (Haak et al. 2015, Mathieson et al. 2015, Lazaridis et al. 2016, Antonio et al. 2019, Mathieson et al. 2018, Marcus et al. 2020, Yang et al. 2020, Papac et al. 2021, Yaka et al. 2021, Patterson et al. 2022) (Tables 1 and 2). In the distal protocols, only *qpAdm* models where target group’s sampling date is strictly contemporaneous with or post-dates sampling of both proxy sources were considered.

We tested performance of these *qpAdm* protocols on complex simulated genetic histories in the shape of admixture graphs: 13 populations connected with each other by admixture graphs of random topology, including 10 pulse-like admixture events. Ten diploid individuals with no missing data were sampled from each population at “leaves” of the graph. Forty such random topologies were simulated, with an upper bound on the graph depth at 800 generations (ca. 23,000 years in the case of humans). These simulations generated sets of populations sampled at widely different dates in the past or, in other words, located at various genetic distances from the root, and matching intra-continental levels of human genetic differentiation (median *F*_*ST*_ per simulated topology ranged from 0.022 to 0.051, see also Fig. S1). Further details on the simulated population histories are presented in Methods and illustrated by 14 examples in Fig. S2. To explore the influence of data amount on *qpAdm* performance and compare it across protocols, we generated two independent sets of ten simulation replicates for each graph topology: with genomes composed of three or ten 100-Mbp-sized chromosomes (see Fig. S3 for the number of SNP loci polymorphic in the simulated datasets). These sets of simulations are referred to as “setup no. 1” and “setup no. 2” below. To explore the parameter space further, we generated single simulation replicates for two further setups: “setup no. 3”, with maximal simulated history depth increased to 3,000 generations (ca. 87,000 yBP for humans), scaling all dates up proportionally; and “setup no. 4”, with all terminal branches extended to the “present” of the simulation and sampled at that point (Fig. 3). These latter simulations generated populations with median *F*_*ST*_ at the inter-continental level (no. 3) or below it (no. 4, Fig. S1).

A typical archaeogenetic dataset is composed of pseudo-haploid data with high proportions of missing sites and with widely different group sizes, including singleton groups. To generate more realistic “noisy” data, we also performed randomized subsampling of SNP datasets for simulation setups no. 1 and 2 (300- and 1,000-Mbp-sized genomes), for one simulation replicate per each setup (Fig. 3, see Methods for details). The resulting data were pseudohaploid, had missing data rates ranging from 5% to 95% across individuals, and had uneven group sizes ranging from 1 to 10 individuals. Ten independent subsampled datasets were generated for each simulated topology (400 replicates in total per simulation setup), including in the case of 300-Mbp-sized genomes from ca. 20,200 to 518,000 SNP loci with no missing data at the group level and polymorphic across 13 groups (median = 89,700), and in the case of 1,000-Mbp-sized genomes from ca. 66,600 to 2,095,700 such SNPs (median = 259,400, Fig. S3). Ranking of key simulation setups by these median site counts is as follows: 300-Mbp-sized genomes, low-quality data; 1,000-Mbp-sized genomes, low-quality data; 300-Mbp-sized genomes, high-quality data; 1,000-Mbp-sized genomes, high-quality data (Fig. S3).

As detailed in the preceding section, various versions of *qpAdm* protocols form a core of many recent archaeogenetic studies. These protocols are aimed at finding the simplest feasible *qpAdm* models for target groups, where feasibility is defined by setting a threshold for *qpAdm/qpWave p*-values and by setting plausibility criteria for admixture proportions estimated by *qpAdm*. Finding a feasible two-way or more complex admixture model for a target is often interpreted as solid evidence for gene flow, especially if PCA and *ADMIXTURE* methods confirm the same signal. Thus, *qpAdm* protocols are used in fact as formal tests for admixture, whereas the latter two methods are not formal tests.

Relying on general principles, we argue that any high-throughput *qpAdm* protocol on poorly understood complex demographic relationships approximated as admixture graphs and/or stepping-stone models is questionable as a formal test for admixture since the *p*-value threshold allows to *reject*, but not to *accept* models, and it is safer to interpret those models as a certain number of gene flows connecting “left” and “right” sets in any direction, not in the form of proxy sources and admixture proportions for a target. The model feasibility criterion including both *p*-values and admixture proportions estimated with *qpAdm* is a complex construct relying on the topological assumptions outlined in Fig. 1. We expect that taking “left” and “right” sets that are not well-separated in time or contemporaneous (Fig. 2), and where relationships among groups are poorly understood (which is almost always true for exploratory studies), enriches the system for “left-to-right” gene flows, which in turn leads to frequent rejection of true simple admixture models. Since the behavior of *qpAdm* admixture proportion estimates under diverse demographic scenarios is poorly understood, it is possible that a large fraction of these non-rejected complex models emerges as feasible, resulting in false signals of admixture. Another approach probably leading to the same outcome (enriching the system for “left-to-right” gene flows and other violations of the topological assumptions) is selection of pairs of “right” populations maximizing deviations of statistics *f*_*4*_(left_*i*_, left_*j*_, right_*i*_, right_*j*_) from zero since such deviations due to tree-like topologies or “left-to-right” gene flows are impossible to tell apart. This approach is sometimes used for increasing the power of *qpAdm* to discriminate between alternative proxy sources.

The *qpAdm* protocols we applied to the simulated data were focused on the simplest models: one- and two-way admixture models (we note that histories that are more complex than two-way mixtures were common in the data, Fig. S2). The model feasibility criterion followed Narasimhan *et al*. (2019), see Box 1 for a definition. Thus, we tested all possible two-way admixture models for 40 complex population histories (34,320 proximal and distal rotating models per simulation setup and replicate).

The non-rotating *qpAdm* approach was implemented as follows: for each simulated graph six most ancient groups were selected as a fixed “right” set (ties were resolved in alphabetical order; these “right” sets remained unchanged for a given graph topology across independent simulations) and for the remaining seven groups all possible one-way and two-way admixture models were tested, applying the same composite feasibility criterion that was used for the rotating protocol (4,200 proximal and distal models per simulation setup and replicate).

### Case studies illustrating false and true feasible qpAdm models

#### False positive models

In the context of complex and random admixture graph topologies it is hard to draw a strict boundary between true and false admixture models composed of a target and only two proxy sources. However, we classified the feasible *qpAdm* models into false and true ones relying on a set of rules. By far the most common class of false feasible *qpAdm* models (referred to as “false positive” or FP models), comprising 50.9% of all FP models generated by the proximal rotating protocol across all the simulation and subsampling replicates (setups no. 1 and 2), occurs when the target group is falsely rejected as forming a clade with one or both proxy sources whilst they are simulated as clades. In other words, these are false rejections of single-source admixture models. Interestingly, under proximal non-rotating protocol across all the simulation and subsampling replicates false rejection of cladality accounted only for 10.1% of FP models, revealing that model violations become ‘visible’ under the rotating protocol.

**Figure 4.**
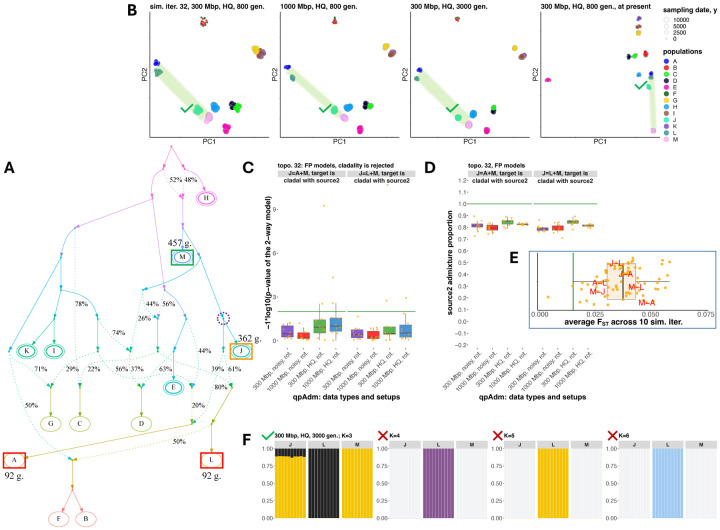
A case study illustrating the most common class of false positive *qpAdm* models supported by the proximal rotating protocol (accounts for 50.9% of all FP models across all the simulation and subsampling replicates in Table 2 and **Suppl. dataset 1**). Models of this type include at least one proxy ancestry source that is simulated as fully cladal with the target. The other proxy source may be simulated as a descendant of the target lineage (as shown here), may belong to an unrelated lineage (Fig. S4a), or may be also cladal with the target. Both models shown here, “*J = A + M*” and “*J = L + M*”, are also fully supported by three-dimensional PCA and by an unsupervised *ADMIXTURE* analysis at one or more *K* values. Below each panel of the figure is described in detail. **A.** Simulated history in the form of an admixture graph (only topology is shown here, for divergence/admixture dates and effective population sizes see the respective simulated history shown in Fig. S2). Sampled populations are marked by letters. The target population from the *qpAdm* models illustrated here is enclosed in an orange rectangle; correct proxy sources – in green rectangles, correct proxy sources with violations of the topological assumptions of *qpAdm* – in green-yellow rectangles, and inappropriate proxy sources, if any – in red rectangles. Sampling dates for these groups (in generations before present) are shown beside the rectangles (the dates are from the “shallow” simulations up to 800 generations deep). True simulated ancestry source(s) for the target are enclosed in dashed circles. Six populations used as outgroups for the non-rotating *qpAdm* protocol are enclosed in double ovals. **B.** Two-dimensional PCA plots for four simulated high-quality datasets indicated in plot titles. Simulated populations are colored according to the legend on the right, and larger points correspond to older sampling dates. The space between the proxy sources is shaded in green. If admixture model(s) are supported by three-dimensional (sic!) PCA (PC1 vs. PC2 vs. PC3) according to the criteria listed in Methods, a green tick mark is placed beside the target group in the plot, and a red cross mark is placed otherwise. **C.** Boxplots summarizing *p*-values of two-way *qpAdm* models (indicated in plot titles) across simulation or subsampling replicates, grouped by simulation setups and *qpAdm* protocols as indicated on the x-axis. Green lines show the *p*-value threshold of 0.01 used in this study for model rejection. *P*-values for one-way models are not shown for brevity. The *qpAdm* models shown here were not tested using the non-rotating protocol since two “left” populations belong to the set of six “right” populations. **D.** Boxplots summarizing estimated admixture proportions across simulation or subsampling replicates, grouped by simulation setups and *qpAdm* protocols. The proportions are shown either for the first or second proxy source, as indicated on the y-axis; green lines show the simulated admixture proportion. **E.** All *F*_*ST*_ values for population pairs from the simulated history. Average *F*_*ST*_ values are shown across 10 simulation replicates for 1000-Mbp-sized genomes and high-quality data. Population pairs formed by components of the illustrated two-way *qpAdm* model(s) are labelled in red. The green line shows median *F*_*ST*_ (0.015) for the Bronze Age West Asian populations analyzed by Lazaridis et al. (2016) as an example of human genetic diversity at the sub-continental scale. **F.** Ancestry proportions estimated with unsupervised *ADMIXTURE* for the groups constituting the *qpAdm* model(s). For brevity, results are shown for one or two *qpAdm* models, for one simulated dataset indicated in the plot title (usually simulation setup no. 3: 300-Mbp-sized genomes, max. divergence date of 3,000 generations, high-quality data), and for four selected *K* values. If admixture model(s) are supported by this analysis at a given *K* value, a green tick mark is placed beside the target group in the plot, and a red cross mark is placed otherwise.

An example of the situation whereby the target group is falsely rejected as forming a clade is shown in Fig. 4 where the clade relationship between the target (*J*) and source (*M*) is rejected by the rotating protocol due “left-to-right” gene flows violating the topological assumptions of *qpAdm*, and more complex models (“*J = A + M*” and “*J = L + M*”) are evaluated as true. When a true one-way model “*J = M*” is tested, *M* represents a proxy for the only true source of ancestry in *J*, and outgroups *A*, *C*, *D*, and *L* are partially derived from the target lineage, resulting in rejection of the one-way model with *p*-values from ~10^−32^ to ~10^−50^ (in the case of 1000-Mbp-sized genomes and high-quality data). Rejections of two-way models “*J = A + M*” and “*J = L + M*” according to *p*-values are rare (Fig. 4c), and nearly all estimated ancestry proportions ±2 SE are between 0 and 1 even on low-quality pseudo-haploid data (Fig. 4d), that is why these models are classified as fitting in nearly all cases (in 34 and 36 of 40 simulation/subsampling replicates, respectively). Groups *A* and *L* are 270 generations younger than *J*, and >50% of their ancestry was simulated as derived from the *J* lineage (Fig. 4a). These FP models illustrate not only cladality rejection, but also incorrect gene flow direction suggested by the *qpAdm* results.

These results illustrate risks of applying rotating *qpAdm* protocols to sets including ancient groups “on the left” and much younger groups “on the right” (Calhoff et al. 2021, Oliveira et al. 2022), since those may be descended partially from the “left” groups (Fig. 1c, d, e) and thus lead to false positive findings of gene flow. Three-dimensional PCA on all four datasets we analyzed (Fig. 4b) and unsupervised *ADMIXTURE* results at some *K* values (Fig. 4f) support these FP models. Thus, a researcher co-analyzing ancient and modern groups due to lack of ancient samples from certain regions may encounter this situation in practice and, given support by all methods in the standard archaeogenetic toolkit (*qpAdm*, PCA, and *ADMIXTURE*), is likely to conclude that FP models are true. We illustrate another similar situation often leading to rejection of cladality and acceptance of two-way or even more complex models (in 33–37 of 40 simulation/subsampling replicates, depending on model and *qpAdm* protocol) in Fig. S4a.

Other topological classes of FP models can be concisely described as follows (and are more precisely defined in Methods): 1) gene flow goes from the target lineage (after the last admixture event in its history) to a proxy source lineage (Fig. 4, Fig. S4a, b); 2) a proxy source included in the model is symmetrically related to all real sources of ancestry in the target (see examples of such feasible *qpAdm* models in Fig. S4c, d), or both proxy sources represent the same true source and are symmetrically related to all the other true sources (Fig. S4e); 3) both proxy sources represent distinct real sources, however a proxy source is a heavily admixed group sharing less than 40% of ancestry with the real source (Fig. S4a, d); 4) the target gets a gene flow from a deep-branching source not represented by any sampled population, and an inappropriate proxy source is included in the fitting model (Fig. S4f, g). Violations of the topological assumptions of *qpAdm* encountered in the examples of FP model classes are described below. All the models shown below were selected among feasible models that were outcomes of the proximal or distal rotating protocols.

In Fig. S4b, the target group, *G*, was simulated as a two-way mixture, and an incorrect proximal model “*G = C + J*” emerged as fitting in 18 of 40 simulation/subsampling replicates. Here, *C* is a group genetically similar to *G* (*F*_*ST*_ < 0.015 in Fig. S4b) and is largely its descendant (87% of ancestry in *C* is derived from the *G/I* lineage, see also Fig. S2c) rather than a proxy source (only 1.7% of its ancestry is derived from one of the true ancestry sources for *G*). Despite the small *F*_*ST*_, the cladality of groups *G* and *C* was rejected in 31 of 40 simulation/subsampling replicates due to simulated gene flows from outgroup (*L* and *M*) lineages into *C* (Fig. S4b; this situation is schematically depicted in Fig. 1a). The model “*G = C + J*” was rejected according to *p*-values only when a lot of data was available (1,000-Mbp-sized genomes and high-quality data, Fig. S4b). This rejection of the incorrect two-way model does not mean that the outcome of *qpAdm* analysis for the target is acceptable since even more complex three-way false models may end up being fitting, although we did not fit three-way models in this study. A correct model we picked as an example, “*G = A + J*”, was universally rejected according to *p*-values (Fig. S4b) due to multiple violations of the topological assumptions: for instance, outgroups *C* and *I* are derived from the target lineage after the admixture event (Fig. 1d). However, removal of various violating outgroups did not make the model “*G = A + J*” and similar models “*G = L/M + J*” fitting. On all datasets the correct model “*G = A + J*” was not supported by PCA and *ADMIXTURE*, whereas the FP model “*G = C + J*” was supported by these methods in most cases (Fig. S4b). Moreover, no true two-way model for group *G* emerged as feasible across the 40 simulation/subsampling replicates we explored in this analysis (**Suppl. dataset 1**): other FP models were “*G = B + C*” (also supported by PCA, see Fig. S4b) and “*G = I + X*”. This case study is another illustration of incorrect inference of gene flow direction by rotating *qpAdm* and of the risk associated with application of rotating protocols in the situation when proxy sources post-date putative target groups (group *C* is much younger than *G* and is a descendant of a population closely related to it, see Fig. S2c). This case study also shows that certain combinations of simulated admixture graph topologies and population sampling patterns create very unfavorable conditions for rotating *qpAdm* protocols and for certain target groups lead to false findings exclusively; and these false findings are supported by other methods such as PCA and *ADMIXTURE*.

In Fig. S4c, the target group, *A*, was simulated as a two-way mixture, and we expect that proximal models “*A = B + C/K*” would be fitting. The topological assumptions are violated when testing these models with the rotating protocol (Fig. S4c): groups *C* and *K* are cladal, with one of them appearing in the “left” set and the other one in the “right” set (this situation is schematically illustrated in Fig. 1e); a gene flow from an outgroup branch, *D*, enters the *C/K* branch after it splits from one of the true ancestry sources (Fig. 1a). However, *p*-values of the models “*A = B + C/K*” were high, but they were universally considered unfeasible due to estimated admixture proportions being negative (“*A = B + K*” is shown as an example in Fig. S4c). Removal of the outgroups *C, D,* and *K* does not make these models fitting. In contrast, incorrect proximal models “*A = B + G/J*” often emerged as fitting. The proxy sources *G* and *J* are symmetrically related to both true ancestry sources in the target. In this case proxy sources *B* and *G/J* do not have equal standing: while group *B* is indeed related to one of the two real sources for group *A*, *G* and *J* are groups completely “external” with respect to the admixture history of *A*. The model “*A = B + G*” shown as an example in Fig. S4c was rejected in some replicates according to *p*-values, but only if a large amount of data was available (1000-Mbp-sized genomes, high-quality data). Overall, it was fitting in 18 of 40 simulation/subsampling replicates (**Suppl. dataset 1**). Both the false (“*A = B + G*”) and true positive (“*A = B + K*”) models discussed as examples here were universally supported by three-dimensional PCA and unsupervised *ADMIXTURE* (at some *K* values) on all datasets analyzed (Fig. S4c). Of 72 feasible two-way models found for group *A* as a target across the 40 simulation/subsampling replicates (**Suppl. dataset 1**), all were classified as FP, and all followed the pattern “*A = B + X*”, with *G*, *J*, *D*, *E*, and *I* being the most common second sources. Another example of an inappropriate proxy source symmetrically related to both true ancestry sources for a target is shown in Fig. S4d: that is distal model “*E = K + L*” which was usually rejected by the rotating protocol if enough data is available (feasible in 6 of 40 simulation/subsampling replicates) but was feasible in the case of the non-rotating protocol (in 34 of 40 simulation/subsampling replicates). This model was also supported by PCA on all four datasets used for this analysis.

In Fig. S4e, the target group, *A*, was simulated as a two-way mixture, and both correct and incorrect two-way models were fitting approximately equally often for this target: across the 40 simulation/subsampling replicates, 38 true positive (TP) models and 47 FP models were found (**Suppl. dataset 1**). We selected two models as examples: both correct (“*A = B + F*”) and incorrect (“*A = F + M*”) proximal models fitted approximately equally often (in 28 and 23 of 40 replicates, respectively), suggesting that violations of the topological assumptions of *qpAdm* play no role in the emergence of this FP model. FDR for target *A* varied between 56% and 61% in the case of low-quality data and/or 300-Mbp-sized genomes; in contrast, for 1,000-Mbp-sized genomes and high-quality data FDR dropped to 14%. Failure to reject the model “*A = F + M*”, where both proxy sources represent only one true ancestry source and are symmetrically related to the other, may be attributed to low *F*_*ST*_ between all the populations involved in both models (Fig. S4e) and to the lack of data: increasing the simulated genome size to 1,000 Mbp and using high-quality data resulted in rejection (according to *p*-values) of the correct model “*A = B + F*” in 4 of 10 replicates, and in rejection of the incorrect model “*A = F + M*” in 9 of 10 replicates (Fig. S4e; **Suppl. dataset 1**). Both models shown here as examples had universal support by PCA and support by unsupervised *ADMIXTURE* (at some *K* values; Fig. S4e). In this case study, the two-way model “*A = F + M*” would lead to misleading historical interpretations: although the target is indeed a two-way mixture, one of the true sources is completely missing in the model.

In Fig. S4d, the target group, *E*, was simulated as a two-way mixture, but no appropriate source proxy was sampled for one of the true ancestry sources: in groups *H* and *A*, 30% and 6% of their ancestry, respectively, is derived from that true source. Thus, multiple gene flows from the “right” set enter the *A* lineage after its split from the true ancestry source, and the same is true for the *H* lineage, and these are violations of the topological assumptions of *qpAdm* (Fig. 1a). The other true source for group *E* has a closely related proxy sampled (group *L*, see Fig. S2g), and *F*_*ST*_ between groups *E* and *L* is low, < 0.015 (Fig. S4d). Despite this low *F*_*ST*_, the (inaccurate) one-way model “*E* = *L*” was rejected by both the rotating and non-rotating *qpAdm* protocols in 39 of 40 simulation/subsampling replicates (**Suppl. dataset 1**). The two-way model “*E = H + L*” was rejected according to *p*-values in 37 of 40 replicates; in contrast, the proximal model “*E = A + L*” was rejected according to *p*-values only in the case of 1,000-Mbp-sized genomes and high-quality data (Fig. S4d). A similar proximal model, “*E = A + M*”, was usually rejected by the rotating protocol (feasible in 6 of 40 replicates; group *L* is closer to the respective real source than group M), but both models “*E = A + L/M*” were feasible if the non-rotating protocol was used (in 36 or 38 of 40 replicates). The ancestry proportion contributed by the proxy sources *L* or *M* was consistently overestimated by all the models and protocols we investigated (Fig. S4d); this appears to be related to the lack of appropriate proxies for the other true ancestry source. Accepting models like “*E = A + L/M*” at face value may lead to erroneous historical interpretations as the “present-day” group *A* is genetically far from the real source, having diverged from it 390 generations ago and having received 94% of its ancestry from other sources (Fig. S2g). But all thresholds for classifying models of this type into false and true are arbitrary. We chose 40% as a threshold percentage of proxy source’s ancestry derived from the corresponding true source. All 212 two-way feasible models for target *E* (across 40 replicates and both rotating and non-rotating protocols) were classified as false (**Suppl. dataset 1**). Since no true model was feasible for target *E*, rejection of an incorrect two-way model given ample data is not an acceptable result, and even more complex false models (which we did not test) may emerge as feasible. Models “*E = A + L/M*” were supported by PCA on all four datasets we explored and by unsupervised *ADMIXTURE* on two of these datasets (Fig. S4d). This case study illustrates again that applying *qpAdm* protocols to poorly sampled regions of the world (where no close proxies are available for some key ancestry sources) and without temporal stratification of “right” and “left” sets may be risky.

In Fig. S4f, the target group, *H*, was simulated as a two-way mixture, with one source being the deepest lineage in the graph, and the other source close to a sampled group, *M*. This situation is similar to the basal Eurasian hypothesis (Lazaridis et al. 2016). A group of FP models was found for this target. One way-models “*H = M*” were almost always rejected (in 39 of 40 simulation/subsampling replicates; **Suppl. dataset 1**), despite low *F*_*ST*_ for this population pair (Fig. S4f), and two-way distal models “*H = F/I/L + M*” were often fitting, especially the model “*H = F + M*” which was fitting in 35 of 40 simulation/subsampling replicates including those with the highest amount of data. Group *F* is the second-deepest lineage in this simulated graph after the true ancestry source for *H*, but it is distinct from the latter. The distal model “*H = F + M*” was universally supported by three-dimensional PCA and negative *f*_*3*_-statistics *f*_*3*_(target; proxy source_*1*_, proxy source_*2*_) (Fig. S4f, Suppl. text 1), as well as strongly supported by the unsupervised *ADMIXTURE* analysis (Fig. S4f) in the case of simulation setup no. 3 (at 7 of 8 *K* values tested). Given the simulated history and population sampling pattern, a researcher relying on the standard archaeogenetic toolkit would conclude that group *H*, sampled 44 generations ago, is derived from a lineage closely related to *M*, sampled 74 generation ago, and an extinct lineage related to a much more ancient group *F* sampled 604 generation ago. While the conclusion about deeply-divergent ancestry in *H* is correct, and the proportion of that ancestry estimated on low- or high-quality data is precise (Fig. S4f), its true source is a separate unsampled lineage not cladal with *F*. Interpretation of such a *qpAdm* model in terms of population history would be misleading if the true divergence point of the deep ancestry is not revealed by an admixture graph analysis similar to that supporting the basal Eurasian hypothesis (Lazaridis et al. 2016). Importantly, all 113 two-way feasible models for target *H* (across 40 replicates and both rotating and non-rotating protocols) were classified as false (**Suppl. dataset 1**).

Another situation where deeply-divergent ancestry in a target is misrepresented by an inappropriate proxy source is model “*F = A + E*” in Fig. S4g. In contrast to the situation in Fig. S4f, the deeply-divergent ancestry in *F* is not unique to this group, but also contributed to several other sampled groups. The distal model “*F = A + E*” was feasible in 24 of 40 simulation/subsampling replicates (for both rotating and non-rotating *qpAdm* protocols; **Suppl. dataset 1**). It was weakly rejected when a lot of data was available (Fig. S4g), and on the lowest-quality data we tested (300-Mbp-sized genomes, noisy data) it was often rejected (e.g., in 5 of 10 replicates analyzed with the rotating protocol) due to negative admixture proportion estimates and/or high *p*-values for one-way models. In contrast to most models discussed above, this model was not supported by PCA and unsupervised *ADMIXTURE* (Fig. S4g). Of 70 two-way feasible models for target *F* (across 40 replicates and both rotating and non-rotating protocols), 69 were classified as false. Thus, this situation would likely be ambiguous for a researcher encountering it in practice.

#### True positive models

Above we have discussed several examples of FP models that in practice would result in misleading conclusions about population history, and now we turn to feasible models that were interpreted as true in our analysis. Two-way admixture models for targets with population history best approximated with three-way and more complex models were considered as true positives if they included at least two source proxies *not* satisfying the topological criteria of false models listed above. We also note that TP models were not restricted to those including most optimal source proxies, if the models do *not* satisfy the false positivity criteria. On a random sample of 400 two-way admixture models from our 40 simulated histories, the fraction of models that were classified as appropriate (true) according to the rules described above was 17.7%. Since groups that are truly admixed are common in our simulations, we do not expect to encounter a “needle in a haystack” situation where finding true admixture models is exceedingly hard.

In Fig. S4h (and Fig. S2a), a set of ideal distal TP models is presented. The target, group *K*, was simulated as a two way-mixture, and close (*H, I*) and more distant proxies (*G, M*) for both true sources were sampled. All four alternative proxy sources display no violations of the topological assumptions of *qpAdm* (Fig. 1), and outgroups such as *L* and *C* related differentially to the true and proxy sources are available (Fig. S4h). An ideal model including the closest proxy sources sampled, “*K = H + I*”, and a nearly ideal model (with one proxy being more distant from the true source), “*K = G + I*”, were nearly universally fitting when the rotating protocol was applied (in 38 and 35 of 40 simulation/subsampling iterations, respectively) and yielded precise estimates of admixture proportions (Fig. S4h). However, the non-rotating protocol demonstrated low power for this target on noisy pseudo-haploid data, resulting in models being unfeasible due to high *p*-values for one-way models and/or negative admixture proportion estimates: the “*K = H + I*” and “*K = G + I*” models were feasible in just 2 or 4 of 20 subsampling iterations, respectively. The two-way models were not rejected according to *p*-values in this case, and had usually non-negative, but imprecise, admixture proportion estimates (Fig. S4h). This observation illustrates the power of rotating protocols (Harney et al. 2021) and the motivation behind their adoption. In the case of high-quality data though, both models were unfeasible in just 1 of 20 simulation iterations analyzed by the non-rotating protocol. Non-optimal models “*K = H + M*” and “*K = G + M*” were rejected according to *p*-values by the rotating protocol (but not the non-rotating one) if enough data was available (Fig. S4h). All 112 two-way feasible models for target *K* (across 40 replicates analyzed by the rotating protocol) were classified as true, but of 142 feasible models that were outcomes of the non-rotating protocol, just 86 were classified as true (**Suppl. dataset 1**). These observations demonstrate again the advantage of the rotating protocol (in rejecting models with non-optimal or inappropriate proxy sources) that exist when there are no violations of the topological assumptions of *qpAdm*. All four models for *K* had universal support by three-dimensional PCA and unsupervised *ADMIXTURE* analyses (at least at some *K* values, see Fig. S4h).

Another series of examples (Fig. S4i–j, Fig. S2c, e) illustrates models that very often emerge as feasible despite imperfect cladality of proxy and true ancestry sources (see schematic representations in Fig. 1a, b). The targets in these cases were simulated as two-way mixtures. Despite non-cladality of one proxy source with the corresponding true source, distal model “*C = I + K*” (Fig. S4i) was usually feasible when both the rotating and non-rotating protocols were applied (in 37 and 30 of 40 simulation/subsampling replicates, respectively) and yielded precise estimates of admixture proportions (Fig. S4i; **Suppl. dataset 1**). The proximal models illustrated in Fig. S4j, “*K =C + D*” and “*K = D + L*”, demonstrated violations of the topological assumptions at one or two proxy sources, were almost universally rejected by the rotating (but not the non-rotating) protocol, and produced estimates of admixture proportions that were always biased (Fig. S4j). Given that the true model in this case is two-way, rejection of a model due to non-cladality of true and proxy sources may lead to erroneous interpretations if more complex (and false) three-way models emerge as feasible. However, as stated above, three-way models were not explored in this study. All true (or false) two-way models for target *K* were rejected by the rotating protocol on high-quality data (300- or 1000-Mbp-sized genomes). Thus, this case study (Fig. S4j) demonstrates a disadvantage of the rotating protocol when all proxies sampled for a certain true ancestry source demonstrate violations of the topological assumptions of *qpAdm*.

Finally, we consider examples of targets that were simulated as three-way mixtures, but for whom two-way *qpAdm* models were often feasible (Fig. S4i, k–m). In this case rejection of such a two-way model is considered a desired outcome and was indeed observed very often when the rotating protocol was applied to high-quality data. In Fig. S4i (target *E*), all three true sources were represented by sampled proxies, however two of them demonstrated non-cladality with the true sources. A two-way model, “*E = C +K*”, was rejected by the rotating (but not the non-rotating) protocol only when a large amount of data was available and, surprisingly, yielded precise estimates of admixture proportions (Fig. S4i). This incomplete (two-way) model for target *E* was also not supported by PCA and *ADMIXTURE* (Fig. S4i). In Fig. S4k, two-way models for a three-way admixed target were almost always rejected by the rotating protocol (but not the non-rotating protocol) according to *p*-values. In one case (“*I = D + J*”) rejection was driven also by negative estimates of admixture proportions (Fig. S4k). The two-way models for target *I* were overwhelmingly supported by PCA and *ADMIXTURE* (Fig. S4k). That is, the third ancestry component in group *I* is largely invisible to these methods. A similar situation is illustrated in Fig. S4l, where both the rotating and non-rotating protocols tend to reject all three two-way models shown (according to *p*-values), but only if a lot of data is available (1000-Mbp-sized genomes and high-quality data). The two-way models for target *H* were also overwhelmingly supported by PCA and *ADMIXTURE* (Fig. S4l). In Fig. S4m, an opposite situation is shown. The model “*D = F + I*” was feasible in nearly all cases (35 of 40 replicates when the rotating protocol was applied and 38 of 40 replicates when the non-rotating protocol was applied; **Suppl. dataset 1**) since proxy source *I* represents two true sources at the same time (Fig. S4m), however it lacked support by PCA and *ADMIXTURE* (Fig. S4m). Ancestry proportions were estimated with a small bias since proxy source *I* does not have the correct ratio of ancestries. We stress that whenever two-way models were feasible for targets modelled as more complex mixtures, they were nevertheless considered as TP in our subsequent analyses.

**Figure 5.**
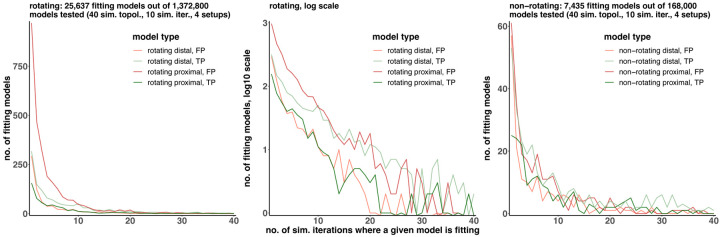
Plots illustrating that for any *qpAdm* protocol or model type (false or true), the vast majority of models only fit the data sporadically. Model feasibility was assessed across the 40 simulation or subsampling replicates analyzed by the rotating and non-rotating *qpAdm* protocols. Models are binned by the number of simulation/subsampling replicates where a model is feasible (fitting) (on the x-axis). Separate decay curves are shown for TP and FP, proximal and distal two-way admixture models. In the case of the rotating protocol, two plots illustrate the same data: a linear-scale plot on the left and a log-scale plot in the center.

The case studies discussed above illustrate advantages of the rotating *qpAdm* protocol over the non-rotating one, but also its problems, especially in the case of proximal models. The admixture models discussed as examples above were feasible in large fractions of the 40 simulation or subsampling replicates (Fig. 3) analyzed with at least one *qpAdm* protocol (rotating or non-rotating), however there is a long tail composed of thousands of models that were just sporadically feasible in our analysis (Fig. 5). This exponential decay pattern is common to FP and TP, proximal and distal models.

Below we move from case studies on selected target groups and selected simulated histories to exploring performance of *qpAdm* protocols on a collection of random complex genetic histories through the lens of FDR. While FP *qpAdm* models are expected for complex genetic histories, the practical usage of *qpAdm* protocols relies on an assumption that false positives are rare. However, FDR of the four *qpAdm* protocols tested here (rotating and non-rotating, proximal and distal) varied between 12.1% and 68.1% (across all simulation setups and replicates summarized in Table 1). Key statistics in our study are false discovery rate (FDR) and false omission rate (FOR); see Box 1 for definitions. We estimated FDR and FOR instead of false positive and false negative rates of the *qpAdm* protocols and other methods due to a technical limitation: the process of model classification into true and false ones cannot be fully automated since it requires careful interpretation of the topology and simulated admixture proportions. Therefore, classifying all possible 34,320 two-way admixture models (858 per simulated topology) into true and false was hardly feasible. We estimated false positive (FPR) and true positive rates (TPR) only approximately, relying on the fractions of negatives and positives in random samples of two-way admixture models (see below). FPR in our context is the probability that a two-way model with an unadmixed target and/or inappropriate proxy source(s) emerges as fitting in a single *qpAdm* test. TPR in our context is the probability that a two-way model with an admixed target and both proxy sources being appropriate emerges as fitting in a *qpAdm* test.

### Influence of the amount of data and temporal stratification on the performance of qpAdm protocols

**Figure 6.**
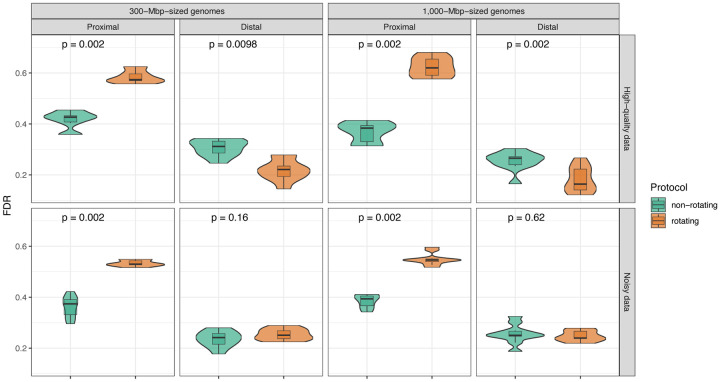
Distributions of FDR values across 10 simulation replicates (for high-quality data) or across 10 subsampling replicates derived from a single simulation replicate (for low-quality data). Distributions are summarized with boxplots and violin plots for four *qpAdm* protocols (proximal and distal, rotating and non-rotating) and two simulated genome sizes (300 Mbp and 1,000 Mbp). *qpAdm* protocols were compared with the paired two-sided Wilcoxon test, and *p*-values for “rotating vs. non-rotating” comparisons are shown in the panels (for the other *p*-values see Table 2).

The amount of data (3.3-fold difference in simulated genome sizes) had no influence on FDR of any *qpAdm* protocols in the case of randomly subsampled pseudo-haploid data (no statistically significant difference was found between sets of 10 subsampling replicates in the case of four *qpAdm* protocols; Table 2, Fig. 6). In contrast, small but statistically significant influence of the data amount on FDR (according to the two-sided Wilcoxon test) was observed for three of four *qpAdm* protocols when applied to high-quality data: proximal rotating and non-rotating, and distal non-rotating (Table 2, Fig. 6). For a complete list including 33,072 feasible two-way *qpAdm* models on which Table 2 and Fig. 6 are based (and for corresponding one-way *qpAdm* models) see **Suppl. dataset 1**.

In the case of the most extreme *qpAdm* protocol, proximal rotating, adding data increased median FDR from 57.4% to 62%, and this difference is statistically significant (Table 2). A large fraction of false positives in the case of the proximal rotating protocol emerges due to false rejections of one-way models because of violations of the topological assumptions (Fig. 1), for instance, due to “left-to-right” gene flows, and that prompts the investigator to test more complex two-way models, which often emerge as feasible (see the case studies above). And model rejection is more efficient when more data is available, explaining the effect on FDR observed here. As we show in Suppl. text 1, it is probably impossible to decrease FDR of the proximal rotating protocol dramatically by combining it with other methods (PCA and unsupervised *ADMIXTURE*) as additional controls or by adjusting *p*-value thresholds in the *qpAdm* protocol.

However, in the case of both proximal and distal non-rotating protocols, adding data led to a small but statistically significant decrease in FDR: 42.6% vs. 38.4%, 31.2% vs. 26.5% (Table 2, Fig. 6), suggesting that false model rejections due to assumption violations play a less important role here, which is expected for “right” and “left” population sets which are stratified temporally. Notably, we found no significant effect of the data amount on FDR in the case of the distal rotating protocol, which demonstrated the best median FDR values overall (as low as 16.4%; Table 2, Fig. 6). We did not compare FDR between high-quality and low-quality datasets with the Wilcoxon test since replicates in these cases were generated differently (in the latter case subsampling replicates were derived from one simulation replicate per simulation setup, while in the former case ten simulation replicates were considered per simulation setup), but we note that random subsampling of SNPs and individuals and a less robust algorithm for calculating *f*_*4*_-statistics (with different statistics calculated on different SNP sets) did not lead to dramatic increases/decreases in FDR (Table 2, Fig. 6).

The fact that the decrease in FDR with increasing amount of data is small is possibly explained in the following way. *P*-values of individual two-way models (FP or “non-ideal” TP models) in most cases decrease exponentially with the amount of data (Fig. S4). However, model rejection is not always “beneficial” for protocol’s performance assessed through FDR since both models with inappropriate proxy sources (false models) and true models with violations of the topological assumptions (Fig. 1) are rejected more often when more data is added.

Next, we assessed the influence of *qpAdm* protocol details on FDR. Temporal stratification of targets and proxy sources (the former are not allowed to pre-date the latter) is the best way of reducing FDR, according to our analysis: from ~50%–60% to ~15%–25% for rotating protocols, and from ~40% to ~25%–30% for non-rotating protocols (Table 2, Fig. 6). All these differences are statistically significant according to the two-sided Wilcoxon test (the paired version of the test was used in this case since different *qpAdm* protocols applied to the same simulation/subsampling replicate are not totally independent experiments). Temporal stratification of “right” and “left” sets (i.e., the non-rotating protocol) is helpful in the absence of the former type of temporal stratification, of targets and proxy sources: FDR drops from ~50%–60% to ~40% (these differences are also statistically significant; Table 2, Fig. 6). However, it is not helpful (no significant difference) or even damaging to *qpAdm* performance (significantly worse) when applied to distal protocols (Table 2, Fig. 6). This result supports the conclusion by Harney et al. (2021) (also illustrated by some examples in the previous section) that rotating *qpAdm* protocols should be preferred to non-rotating ones. However, according to our analysis, this conclusion is conditional on strictly enforced temporal stratification of targets and proxy sources since the rotating protocol without such stratification (“proximal rotating”) demonstrated by far the worst performance in our analysis. There is a possibility that this conclusion is also conditional on the underlying history being approximated as a complex graph, but not as a stepping-stone model.

Another observation is that absolute FDR values are high for all *qpAdm* protocols tested (median FDR values below 16.4% were not observed), however these absolute values are expected to depend on the complexity of simulated histories and on the amount of data (Table 2), which also depends on the time depth of simulated histories (Fig. S3, see Discussion).

The fraction of two-way admixture models that are inappropriate according to our topological criteria (the fraction of negatives) in a random sample of 400 models from all simulated graph-shaped genetic histories is 82.3%, which allows us to approximately estimate not only FDR (51.6%–68.1%, see Table 1), but the FPR of the proximal rotating protocol = number of false positives per simulation replicate / number of negatives per simulation replicate [858 models × 40 graphs × fraction of negatives] = 0.4%–2.6% across simulation parameter combinations and replicates summarized in Table 2. The TPR of the proximal rotating protocol = number of true positives per simulation replicate / number of positives per simulation replicate [858 models ´ 40 graphs × (1 – fraction of negatives)] = 1.1%–10.1%. Here are the same statistics for the distal non-rotating protocol: the fraction of negatives in a random sample of 400 distal non-rotating models from all simulated graphs, 74.3%; total number of distal non-rotating two-way models across all graph topologies, 1804; FDR across simulation parameter combinations and replicates from Table 2, 16.4%–34.4%; FPR, 0.8%–3.2%; TPR, 9.7%–24.4%. Thus, although FPR of a single *qpAdm* test applied to a random graph-shaped genetic history is low, due to the high proportion of negatives among all models (Fig. 5), the large number of models tested in high-throughput *qpAdm* screens, and the low TPR, FDR becomes high, compromising historical interpretation of such screens for admixture.

**Figure 7.**
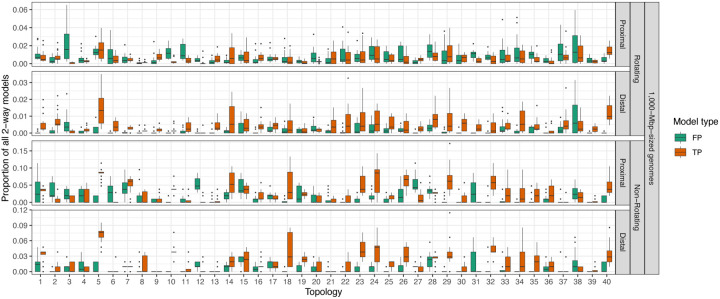
Proportions of all possible two-way *qpAdm* models that are false positive (FP), or true positive (TP), binned by simulated graph topology. There are 858 two-way admixture models per simulated topology including 13 groups if the rotating *qpAdm* protocol is applied, and 105 models if the non-rotating protocol with 6 outgroups is applied. For brevity, results are shown for 1000-Mbp-sized genomes and high-quality data only. The boxplots summarize distributions of FP and TP model fractions across simulation replicates.

The fraction of feasible *qpAdm* models that are false (FP) varies substantially depending on topology of simulated graph-shaped genetic history (Fig. 7), and hence it is hard to predict if a particular real genetic history is prone to generating FP signals of admixture when *qpAdm* protocols are applied. Among 80 combinations of proximal *qpAdm* protocols (rotating or non-rotating), simulation setups, and simulation/subsampling replicates we tested, in one combination only a topology accounts for >20% of FP *qpAdm* models found across all the 40 simulated topologies. In contrast, in the case of distal *qpAdm* protocols, results are much more uneven across topologies: for 25 of 80 “protocol/simulation setup/replicate” combinations, at least one topology accounts for >20% of FP *qpAdm* models found across all the 40 simulated topologies. For an illustration of the three topologies most problematic for distal *qpAdm* protocols see Fig. S5. These differences in *qpAdm* performance across topologies are probably driven at least in part by the fact that some simulated graph-shaped histories have much “denser” violations of the *qpAdm* topological assumptions than others, but this frequency of assumption violations was not quantified in our study since it requires another layer of manual topological analysis.

### Admixture inference pipelines and model competition qpAdm protocols

An implicit assumption of many archaeogenetic studies relying on *qpAdm* protocols is that admixture models supported by clines observed in (usually two-dimensional) spaces of principal components, and/or by an *ADMIXTURE* analysis, and/or by individual *D*-, *f*_*4*_- or *f*_*3*_-statistics are especially robust. And, *vice versa*, *qpAdm* results are often interpreted as a formal test of hypotheses about admixture formulated based on PCA and/or *ADMIXTURE* results. We constructed “admixture inference pipelines” composed of a *qpAdm* protocol and one or two further methods to test these assumptions on simulated data. We note that all patterns in our PCA or *ADMIXTURE* analyses consistent with admixture were not explored with *qpAdm*. *Vice versa*, all feasible *qpAdm* models were checked by the PCA and/or *ADMIXTURE* methods.

We considered a two-way admixture model to be supported by PCA if the target group was located on a straight line between the two proxy source groups in the space of first three PCs when all 13 simulated groups were co-analyzed on high-quality data. Deviation from the straight line due to post-admixture genetic drift was acceptable to a certain extent (see Methods) considering theoretical results by Peter (2022). Non-linear PCA clines are often observed on real data (de Barros Damgaard et al. 2018, Jeong et al. 2019), and they were also common among TP two-way *qpAdm* models in this study (see Methods for details and Fig. 4 and Fig. S4 for examples). This situation is expected since many target groups in our simulations represent three-way and more complex mixtures (see examples in Fig. S4k–m), and since arrangement of populations in PC spaces is influenced not only by admixture of previously isolated groups or continuous gene flows decaying with distance (2016 Duforet-Frebourg and Slatkin 2016), but also by genetic drift (McVean 2009, Peter 2022). Our requirements for a model to be declared supported by PCA were more stringent than those usually applied in the literature since we considered three-dimensional PC spaces instead of two-dimensional ones (however, 2D PCAs are shown in Fig. S4 for clarity). Also see Methods for the rules we used to judge if an admixture model is supported by an unsupervised *ADMIXTURE* analysis and see Fig. 4 and Fig. S4 for examples.

A much more limited form of group rotation, “model competition”, is used in the literature widely (Narasimhan et al. 2019, Fernandes et al. 2020, Calhoff et al. 2021, Sirak et al. 2021, Zhang et al. 2021, Maróti et al. 2022, Brielle et al. 2023, Lee et al. 2023), and we explored FDR of this method as well. A typical model competition protocol (Narasimhan et al. 2019, Maróti et al. 2022, Brielle et al. 2023) consists of two stages. First, the oldest, e.g., Palaeolithic, populations (and/or those most divergent from the target group) are used as a fixed “right” set, and populations sampled at later dates are used as proxy sources and targets. As usual, progressively more complex models are tested for targets of interest, and a composite feasibility criterion is applied.

In many publications (e.g., Haak et al. 2015, Mathieson et al. 2015, Antonio et al. 2019, Mathieson et al. 2018, Prendergast et al. 2019, Marcus et al. 2020, Papac et al. 2021, Wang et al. 2021, Yaka et al. 2021, Changmai et al. 2022a, 2022b, Patterson et al. 2022) this first non-rotating step remains the only *qpAdm* protocol used (in its distal or proximal forms). In a model competition protocol, subsequent analysis is focused on targets for whom two or more alternative *qpAdm* models emerge as feasible at the first step. For each target, alternative proxy sources are pooled and rotated between the “left” and “right” sets, testing only the models that emerged as feasible at the first step and applying a composite feasibility criterion (e.g., *p*-value > 0.01, estimated admixture proportions ± 2 SE are within 0 and 1).

Rotation of alternative proxy sources can be performed in various ways: “whatever is not on the left is on the right” (Brielle et al. 2023), or placing alternative sources in the ”right” set one by one (Calhoff et al. 2021, Maróti et al. 2022, Brielle et al. 2023). In the latter case several “right” sets are tested for each model, and the model is considered supported by the model competition protocol only if it is not rejected under any of these “right” sets (Maróti et al. 2022). The reasoning behind this protocol is as follows: model rejection due to violations of the topological assumptions of *qpAdm* is not expected for a model composed of sources very close to the true ones since in this case branches private to the proxy sources are short, and it is unlikely that gene flows to or from the “right” population set happened on these short branches. Models composed of sources closely related to the true ones are also not expected to be rejected when more distant proxy sources are placed in the “right” set (Harney et al. 2021; see also a case study in Fig. S4h).

For reasons detailed in the Discussion section, we explored the *qpAdm* model competition protocol and multi-method admixture inference pipelines on one replicate per simulation setup, and three (Table 3) or four (Table 1) simulation setups were involved in this analysis. The two alternative model competition protocols described above were applied to targets for whom more than one model was feasible given a fixed “right” set composed of six groups. If only one model was feasible for a target, such a model was evaluated as passing model competition (such models accounted only for 7%–11% of models feasible at the first step). The model competition protocols failed to improve *qpAdm* performance: FDR ranged from 29% to 46% (as compared to 36%–46% prior to the model competition step), and both model competition protocols demonstrated very similar results (Table 3). FOR of the model competition protocols varied from 59% to 72%. FDR also remained high for models supported by proximal non-rotating *qpAdm* & PCA or by proximal non-rotating *qpAdm* & model competition & PCA (Table 3).

Considering all the simulation setups and replicates shown in Table 2, there were only 1,591 instances when a two-way admixture model was supported by both the proximal rotating and proximal non-rotating protocols on the same simulated data (this corresponds to 1,591´2 = 3,182, or 9.6%, of 33,072 unique combinations of *qpAdm* protocols, simulated datasets, and feasible two-way admixture models that we found; see **Suppl. dataset 1**). In contrast, there were 5,844 (17.7%) and 24,046 (72.7%) instances when a model was supported exclusively by the proximal non-rotating and rotating protocols, respectively. Notably, FP models supported by the proximal non-rotating *qpAdm* protocol largely lacked support by an unsupervised *ADMIXTURE* analysis (Table 3), in contrast to outcomes of the proximal rotating protocol (Table S1). FDR of a pipeline composed of these two methods ranged from 5% to 21% across three simulation setups tested (Table 3). Adding a model competition step to this pipeline increased both FDR and FOR in 4 of 6 cases; and, in general, the proximal non-rotating *qpAdm* protocol combined with *ADMIXTURE* is the bestperforming protocol in this analysis (Table 3) according to FDR and FOR.

The fact that our *ADMIXTURE* analysis supports a large fraction of FP two-way mixture models emerging as outcomes of the proximal rotating *qpAdm* protocol reflects known problems in modelling with *ADMIXTURE* very ancient individuals in the context of modern populations. These individuals are often modelled (Raghavan et al. 2014, Haak et al. 2015, Moreno-Mayar et al. 2018a) as complex mixtures of ancestry components typical for modern populations, which is obviously an artefact. Sampling dates for unique targets from FP models supported by both proximal rotating *qpAdm* and *ADMIXTURE* ranged from the present to 665 generations in the past (median = 406 generations), while the median sampling date for unique targets from both FP and TP models supported by proximal rotating *qpAdm* was 215 generations in the past (for comparison across simulation setups, all the dates were rescaled to a maximum simulation depth of 800 generations). The proximal non-rotating protocol by design did not consider the oldest groups as targets for *qpAdm* and *ADMIXTURE* analyses, thus largely avoiding this problem (sampling dates for unique targets from FP models supported by both proximal non-rotating *qpAdm* and *ADMIXTURE* ranged from the present to 366 generations in the past; median = 44 generations).

Above we have discussed multi-method pipelines based on the proximal non-rotating *qpAdm* protocol. Combining distal *qpAdm* protocols with PCA allows to reduce FDR of both rotating and non-rotating protocols further, to 10%–24%, and distal *qpAdm* protocols combined with an unsupervised *ADMIXTURE* analysis demonstrated even better FDR values ca. 0%–8% (Table 1). If target and proxy source populations are sampled at approximately the same time (such as those from our simulation setup no. 4 and from the proximal analysis in Narasimhan et al. 2019, Fig. 2c) applying this approach is impossible. However, if our simulations with all branches extended to the present are treated in the same way as their topological counterparts with date-variable sampling, performance gains (decrease in FDR) of temporal stratification of admixture models are similar to those mentioned above (Table 1). In this case the temporal stratification procedure retains models with the latest admixture event in target’s history that is more recent than (or as recent as) the latest admixture events in proxy sources’ history. However, in the case of simulation setup no. 4 performance gains of the “*qpAdm* + PCA” and “*qpAdm* + *ADMIXTURE*” method combinations were moderate (Table 1).

## Discussion

In this study we explored performance of various *qpAdm* protocols on a collection of simulated genetic histories in the shape of complex admixture graphs of random topology and the same complexity, where admixture history of target groups may vary from the simplest (no admixture) to very complex. It is because of this research design and other limitations discussed below that our study is focused mostly on one performance metric: false discovery rate or FDR. In simple terms, we focused our analysis only on models of a chosen complexity class (two-way models) supported by a *qpAdm* protocol (feasible models), classified them manually into false and true positives according to a set of topological rules, and subjected them to further screening by PCA and/or *ADMIXTURE* methods. We did not attempt to classify rejections of two-way models by *qpAdm* or other methods into false rejections due to violations of the topological assumptions of *qpAdm* (Fig. 1) and true rejections when the true admixture history of the target does not fit a two-way model. This problem was deliberately left out since in the literature more attention is paid to interpretation of “fitting” (“feasible” or “positive”) than of rejected *qpAdm* models.

Another limitation of our study is that we had to use idealized versions of *qpAdm*, PCA, and *ADMIXTURE* protocols, while in the archaeogenetic literature manual adjustment of analytical protocols is common: protocols often vary from one target group to another (see, e.g., Lazaridis et al. 2016, Zhang et al. 2021, Brielle et al. 2023, Lee et al. 2023) and from study to study. These extensive details are very hard to formalize and reproduce. In the case of *qpAdm* protocols, certain groups of populations may be placed exclusively in “right” or in “left” sets, with the rest rotated between these sets, and relative sizes and compositions of these three groups vary from study to study: in the case of model competition protocols, this rotated subset is small, and rotation may be restricted to a particular model complexity class, but in other cases it may encompass all or nearly all populations analyzed (see, e.g., Narasimhan et al. 2019, Librado et al. 2021, Bergström et al. 2022, Lazaridis et al. 2022, Oliveira et al. 2022, Taylor et al. 2023). Reproducing all aspects of PCA and *ADMIXTURE* protocols used in the literature is also hardly possible on simulated data. For instance, PCs in archaeogenetic studies are usually calculated based on present-day populations, and ancient individuals are projected on the resulting PCs (e.g., Haak et al. 2015, Mathieson et al. 2018, Narasimhan et al. 2019, Furtwängler et al. 2020, Marcus et al. 2020, Lazaridis et al. 2022). In contrast, in our study all simulated individuals were co-analyzed for calculating PCs since this analysis was based on high-quality data only. Unsupervised *ADMIXTURE* analyses in the literature are usually performed on worldwide or continent-wide panels of populations that often overlap just partially with population sets used for *qpAdm* analyses (see, for instance, Rasmussen et al. 2010, Haak et al. 2015, Harney et al. 2018, Moreno-Mayar et al. 2018a, Zhang et al. 2021, Changmai et al. 2022a, Brielle et al. 2023), while in our study identical population sets were used for *qpAdm*, PCA, and *ADMIXTURE* analyses.

Another important caveat is that complexity of genetic history in the region and period of interest often remains unknown and it is difficult to judge if a particular admixture graph complexity is adequate for simulating the real history. However, we have not explored *qpAdm* performance over a range of simulated admixture graph complexities, over a range of model feasibility criteria (except for those in Table S2), for models more complex than two-way, and have estimated FDR and FOR instead of false positive and false negative rates due to an important technical limitation: the process of model classification into true and false ones cannot be fully automated since it requires careful interpretation of the simulated topology and simulated admixture proportions (this is illustrated by the case studies in Fig. S4). For similar reasons, some comparisons of method performance in this study, such as *qpAdm* vs. “*qpAdm* combined with *ADMIXTURE*”, are qualitative rather than quantitative: we applied the PCA and *ADMIXTURE* methods to one simulation replicate only per simulation setup since automated classifiers of admixture models into positive and negative ones based on 3D PCA and *ADMIXTURE* results were not available. Despite these limitations, our simulations reproduce the most important aspects of typical *qpAdm* protocols.

Finally, a notable limitation of our analysis is that isolation by distance (stepping-stone) models were not considered. Strictly speaking, it remains unknown if admixture history of humans, wolves (Bergström et al. 2022), and horses (Librado et al. 2021, Taylor et al. 2023), to cite some recent examples from the archaeogenetic literature, is approximated reasonably well by complex admixture graphs composed of isolated populations and episodes of admixture, or rather by two-dimensional stepping-stone models (with additional features such as non-symmetric gene flows, extinction and repopulation), or a combination thereof. Performance of *qpAdm* on a very simple one-dimensional stepping-stone history was explored just briefly by Harney et al. (2021) and was deemed poor.

We demonstrated that application of the proximal rotating *qpAdm* protocol that can be summarized as “whatever is not on the right is on the left” without any temporal stratification of the “right” and “left” sets and of proxy sources and targets carries a risk of an FDR above 50% or 60%. Adding further levels of support (considering only models supported by PCA and/or an *ADMIXTURE* analysis) does not help to decrease FDR drastically in this case (Table S1, Suppl. text 1).

The proximal rotating protocol is an extreme example of *qpAdm* protocols that is rarely encountered in the archaeogenetic literature (Calhoff et al. 2021, Oliveira et al. 2022) but serves as a reference point in our analysis. Other protocols such as distal rotating (e.g., Narasimhan et al. 2019, Librado et al. 2021, Allentoft et al. 2022, Bergström et al. 2022, Lazaridis et al. 2022, Taylor et al. 2023), distal non-rotating (e.g., Haak et al. 2015, Mathieson et al. 2015, Lazaridis et al. 2016, Antonio et al. 2019, Marcus et al. 2020, Yang et al. 2020, Papac et al. 2021, Yaka et al. 2021, Patterson et al. 2022), and proximal model competition (e.g., Narasimhan et al. 2019, Calhoff et al. 2021, Zhang et al. 2021, Maróti et al. 2022, Brielle et al. 2023, Lee et al. 2023) are often used in practice, and FDR of these three classes of protocols on our simulated data ranged from 12% to 46% across simulation parameter combinations and replicates (Tables 1 and 3). These FDR for best-performing standalone *qpAdm* protocols are high but should not be over-interpreted since they are expected to depend on the complexity of simulated histories and on the amount of data (Table 2), which also depends on the time depth of simulated histories (Fig. S3). Only one graph complexity level was tested, that is 13 groups and 10 admixture events; and only one time depth, 800 generations, was tested in a high-throughput way (Table 1). Thus, it is hard to extrapolate this aspect of our results to real analyses and predict FDR levels on real data.

Temporal stratification tested in this study and practiced in the literature is of two sorts: 1) most or all populations in the “right” set are sampled deeper in the past than those in the “left” set (non-rotating protocols); 2) a target group post-dates (or is as old as) all its proxy sources (distal protocols). We showed that both temporal stratification approaches helped to decrease FDR of *qpAdm* admixture screens significantly, and the latter approach demonstrated the best FDR among standalone *qpAdm* protocols (Table 2).

Although restricting analyses to distal models is often *necessary* for reducing FDR below an arbitrary threshold at 10%, it is not *sufficient* for reaching this objective (Table 1) given the complexity of our simulated admixture graph-shaped histories and the amounts of data we generated. Respecting this threshold, only the following admixture screening protocols demonstrated acceptable performance (we did not consider protocols demonstrating FOR above 90% as useful in practice):

1. proximal non-rotating *qpAdm* with a requirement that admixture models are supported by both *qpAdm* and an unsupervised *ADMIXTURE* analysis (Tables 1 and 3), under simulation setups no. 1 and 2 (groups sampled at different dates in the past, maximal simulated history depth = 800 generations, 300-Mbp-sized or 1000-Mbp-sized genomes simulated);
2. distal non-rotating or rotating *qpAdm* with a requirement that admixture models are supported by both *qpAdm* and an unsupervised *ADMIXTURE* analysis (Table 1), under simulation setups no. 1, 2, and 3 (groups sampled at different dates in the past, maximal simulated history depth = 800 or 3000 generations, 300-Mbp-sized or 1000-Mbp-sized genomes simulated).

FDR of these protocols was 0% – 8% (Table 1). In contrast, adding a model competition step to the proximal non-rotating *qpAdm* protocol did not help to reduce FDR below 10%. The performance of this type of protocols is explored in detail in Table 3.

### Best practices

As outcomes of this study, we make the following suggestions for improving robustness of admixture inference in archaeogenetics:

1. Our results suggest that temporal stratification of targets and proxy sources is a very efficient way of reducing FDR of *qpAdm* protocols (Tables 1 and 2, Fig. 6). The distal rotating and non-rotating protocols invariably demonstrated FDR significantly lower than those of the proximal non-rotating and rotating protocols (Table 2). Although the proximal model competition protocol (Narasimhan et al. 2019, Calhoff et al. 2021, Zhang et al. 2021, Maróti et al. 2022, Brielle et al. 2023, Lee et al. 2023) was not tested on multiple simulation or subsampling replicates (Table 3), we note that it demonstrated FDR values higher than those of the distal non-rotating protocol (Table 1). These results and our case studies (Fig. 4, Fig. S4a–e) imply that *qpAdm* protocols where all populations are sampled at present (similar to our setup no. 4; see also Jeong et al. 2019, Changmai et al. 2022a) or where present-day groups are used as proxy ancestry sources for ancient groups (e.g., Mathieson et al. 2015, van de Loosdrecht et al. 2018, Narasimhan et al. 2019, Prendergast et al. 2019, Shinde et al. 2019, Wang et al. 2020, Wang et al. 2021, Changmai et al. 2022b, Calhoff et al. 2021, Oliveira et al. 2022) are less reliable than those where target groups are not allowed to pre-date their proxy sources. While the proximal non-rotating *qpAdm* protocol demonstrated FDR significantly lower than that of the proximal rotating protocol (Table 2), the distal rotating protocol was in terms of FDR as good as the distal non-rotating protocol (on low-quality data) or significantly better (on high-quality data, Table 2).
2. Another way of radically improving FDR of *qpAdm* protocols is combining *qpAdm* with an unsupervised *ADMIXTURE* analysis. These two approaches should possibly be combined for optimal performance (Table 1).
3. Adding 3.3 times more data led to a small but significant decrease in FDR only in the case of high-quality diploid data, but not in the case of pseudo-haploid data with high missing rates (Table 2). This observation deserves further investigation.
4. It is safest to use the *qpAdm* method in controlled conditions, when relationships among populations are understood well enough to exclude violations of the topological assumptions, when radiocarbon or context dates of ancient populations are reliable and allow accurate temporal stratification, or when sets of potential proxy sources are well-constrained based on archaeological or historical scholarship: see, for instance, the earliest publications where the *qpAdm* method was employed (Haak et al. 2015, Mathieson et al. 2015) and some recent studies (e.g., Marcus et al. 2020, Papac et al. 2021, Yaka et al. 2021, Changmai et al. 2022a, 2022b, Patterson et al. 2022). Obviously, the amount of new information that the *qpAdm* method provides in these conditions is limited. However, considering that it was possible to reach FDR levels as low as 0% to 8% on our simulated data, we do not recommend avoiding *qpAdm*-based high-throughput admixture screens altogether.
5. Summing up all the results above, for reducing FDR of *qpAdm* admixture screens to nearly 0% we suggest using large SNP sets with low missing data rates, using the rotating *qpAdm* protocol with a strictly enforced rule that targets do not pre-date their proxy sources, and performing an unsupervised *ADMIXTURE* analysis to verify feasible *qpAdm* models.
6. Our study has multiple limitations and caveats discussed above, mostly related to difficulties in simulating all the details of published *qpAdm*, PCA, and *ADMIXTURE* protocols, to uncertainties about the level of admixture graph complexity that is adequate for simulating real population histories (all our simulations were of the same complexity: 13 groups and 10 pulse-like admixture events), to uncertainty about applicability of admixture graph models in general since stepping-stone models may constitute a more adequate simplification if human population history, to difficulties in interpreting topologies of random admixture graphs in an automated way for classifying even simple admixture models into true and false ones, and to difficulties in interpreting 3D PCA and *ADMIXTURE* results in an automated way. Nevertheless, our results surpass in scale previous simulation studies of *qpAdm* protocols (Lazaridis et al. 2017, Ning et al. 2020, Harney et al. 2021) by several orders of magnitude and may serve as a guide for users of high-throughput *qpAdm* protocols.
7. Feasible *qpAdm* models are sometimes ranked by *p*-values, with a model having the highest *p*-value highlighted as the most plausible one (see, for instance, Lazaridis et al. 2022, van de Loosdrecht et al. 2018, Oliveira et al. 2022, Taylor et al. 2023). *qpWave p-*values for pairs of individuals were also used in lieu of genetic distances in the former study (Lazaridis et al. 2022). Of 1,201 instances when both false and true feasible *qpAdm* models were found for the same target group on the same data (all simulation setups, simulation/subsampling replicates, and *qpAdm* protocols), a model having the highest *p-*value was an FP in 463 (38.6%) cases, and the difference in maximal *p*-values between the TP and FP model classes was significant according to the paired two-sided Wilcoxon test (TP > FP, *p*-value = 2.2 × 10^−16^). Thus, our limited analysis suggests that the approach of ranking *qpAdm* models by *p*-values is justified (see also related results in Fig. S6 and Table S2), but it generates noisy results.
8. *. f*_*3*_-statistic is a simple method for proving that a population is admixed, and it demonstrated FDR values much lower (6%, see Suppl. text 1) than those of standalone *qpAdm* protocols, but *f*_*3*_-statistics are applicable only to recent admixture events and/or populations of large effective size since post-admixture drift on the target lineage obscures the signal (Patterson et al. 2012, Peter 2022). Moreover, calculating *f*_*3*_-statistics for a target composed of a single pseudo-haploid individual is impossible since a heterozygosity estimate is required (Maier et al. 2023), and such singleton groups are common in archaeogenetic studies. Researchers should also be aware that *f*_*3*_-statistics are defined on unrooted trees, and that may lead to rare but strong false signals of admixture (Fig. S4f).

The risks of interpreting *qpAdm* results for proximal models discussed above are apparently due to topological properties of the histories in the form of admixture graphs that we simulated: complexity of genetic history of a sampled population randomly picked from these graphs tends to grow with time from the root, as well as the chance to encounter violations of the topological assumptions of *qpAdm* (Fig. 1) grows with the length of the branch between a true ancestry source and a sampled proxy source or between a target group when it is formed by admixture and when it is sampled. Although it is very often assumed that real genetic history of humans and many other species is reasonably approximated as complex admixture graphs with these properties, this assumption remains unproven.

## Methods

### Simulating random admixture graphs with msprime v.1.1.1

For simulating genetic data, we used *msprime v.1.1.1* which allows accurate simulation of recombination and of multi-chromosome diploid genomes relying on the Wright-Fisher model (Nelson et al. 2020, Baumdicker et al. 2022). We simulated three or ten diploid chromosomes (each 100 Mbp long) by specifying a flat recombination rate (2 ×10^−8^ per nt per generation) along the chromosome and a much higher rate at the chromosome boundaries (log_e_2 or ~0.693 per nt per generation, see https://tskit.dev/msprime/docs/stable/ancestry.html#multiple-chromosomes). A flat mutation rate, 1.25×10^−8^ per nt per generation (Scally & Durbin 2012), and the binary mutation model were used. To maintain the correct correlation between chromosomes, the discrete time Wright-Fischer model was used for 25 generations into the past, and deeper in the past the standard coalescent simulation algorithm was used (as recommended by Nelson et al. 2020).

Genetic histories in the form of admixture graphs of random topology including 13 populations and 10 pulse-like admixture events were generated using the *random_admixturegraph* and *random_sim* functions from the *ADMIXTOOLS 2* package (https://uqrmaie1.github.io/admixtools/reference/random_sim.html), which produced scripts for running the *msprime v.1.1.1* simulator. Demographic events were separated by date intervals ranging randomly between 20 and 120 generations, with an upper bound on the graph depth at 800 generations (or ca. 23,000 years in the case of humans). In another set of simulations, all the dates were scaled up 3.75 times, with an upper bound on the graph depth at 3,000 generations (or 87,000 years in the case of humans). To be more precise, demographic events were not placed in time entirely randomly, but were tied to one or few other events of the same “topological depth” within the graph, as illustrated by ten examples of simulated topologies in Fig. S2. The same principle was applied to sampling dates, which were tied to other demographic events such as divergence and admixture of other populations. These restrictions were used to ensure topological consistency of random graphs.

Ten diploid individuals with no missing data were sampled from each population at “leaves” of the graph. Effective population sizes were constant along each edge and were picked randomly from the range of 1,000 – 10,000 diploid individuals. Admixture proportions for all admixture events varied randomly between 10% and 50%. This setup generates groups sampled at widely different dates in the past or, in other words, located at various genetic distances from the root. Alternatively, all terminal branches were extended to the “present” of the simulation and sampled at “present”, keeping their respective effective population sizes and topological relationships unchanged. Thus, another set of simulations was generated for the same topologies, where groups were more drifted with respect to each other (see *F*_*ST*_ distributions in Fig. S1).

In summary, four sets of independent simulations (“simulation setups”) differing by the amount of data generated and by population divergence metrics were performed for a set of 40 random admixture graph topologies (see an overview in Fig. 3):

1. three 100-Mbp-sized chromosomes; groups sampled at different points in time; maximal simulated history depth at 800 generations (10 simulation replicates, median number of polymorphic sites = 669,655, see Fig. S3);
2. ten 100-Mbp-sized chromosomes; groups sampled at different points in time; maximal simulated history depth at 800 generations (10 simulation replicates, median number of polymorphic sites = 2,229,459);
3. three 100-Mbp-sized chromosomes; groups sampled at different points in time; maximal simulated history depth at 3,000 generations (one simulation replicate, median number of polymorphic sites = 1,074,336);
4. three 100-Mbp-sized chromosomes; all terminal branches extended to the “present” of the simulation and sampled at that point; maximal simulated history depth at 800 generations (one simulation replicate, median number of polymorphic sites = 838,297).

To create more realistic datasets, we performed randomised subsampling of polymorphic sites and individuals (replicates no. 1 of the first and second simulation setups were used for this, see the list above). First, we randomly sampled alleles at heterozygous sites, creating pseudo-haploid data. Then we introduced missing data by randomly selecting a missing rate between 5% and 95%, followed by randomly selecting sites according to the missing rate. This site subsampling was repeated for each individual independently. Lastly, we randomly sampled *n* (from 1 to 10) individuals from each population independently. The subsampling procedure described above was conditioned on the number of sites polymorphic in the set of 13 simulated populations and was repeated until a subsampling replicate with more than 20,000 (for 300-Mbp-sized genomes) or 66,000 such sites (for 1000-Mbp-sized genomes) was obtained. We generated 10 independent subsampled replicates for each topology and simulation setup (800 replicates in total).

Polymorphism data in the *EIGENSTRAT* format were generated from the tree sequences using the *TreeSequence.genotype_matrix* function (https://tskit.dev/tskit/docs/stable/pythonapi.html#tskit.TreeSequence.genotype_matrix) and used for all subsequent analyses (*f*-statistics and *qpAdm*, PCA, *ADMIXTURE*).

For all the work on *f*-statistics and *qpAdm*, the *ADMIXTOOLS 2* software package (Maier et al. 2023) was used. For diploid SNP sets without missing data, we first calculated all possible *f*_*2*_-statistics for 4-Mbp-sized genome blocks (with the “*maxmiss=0*”, “*adjust_pseudohaploid=TRUE*”, and “*minac2=FALSE*” settings) and then used them for calculating *f*_*3*_- and *f*_*4*_-statistics as linear combinations of *f*_*2*_-statistics and for testing *qpAdm* models using the *qpadm* function in *ADMIXTOOLS 2* (https://uqrmaie1.github.io/admixtools/) under default settings. Inferred admixture proportions were not constrained between 0 and 1. For pseudo-haploid SNP sets with missing data and uneven group sizes, the *qpadm* function was applied directly to genotype files, with the “*allsnps=TRUE*” setting. In other words, *f*_*4*_-statistics utilized by *qpAdm* and *f*_*3*_-statistics were calculated off the genotype files without intermediate *f*_*2*_-statistics, and removal of missing data was done for each population quadruplet or triplet separately. This setup is often used in the literature in the presence of missing data (e.g., Harney et al. 2018, Harney et al. 2019, Narasimhan et al. 2019, Lazaridis et al. 2022).

### Rotating qpAdm protocols

*QpWave* tests were performed on sets of 13 groups divided randomly into 2 “left” and 11 “right” groups, testing all possible bisections of this form. *QpAdm* was applied to the same sets of 13 groups divided randomly into 3 “left” and 10 “right” groups, testing all possible bisections of this form for all possible target groups in “left” sets (858 such models per simulated history). This proximal rotating protocol was applied to all simulation setups. Subsequent work was focused only on feasible *qpAdm* models defined as follows: 1) *p*-values calculated by *qpWav*e for one-way models “target = proxy source_*1*_”, “target = proxy source_*2*_”, and “proxy source_*1*_ = proxy source_*2*_” are all below 0.01; 2) in the case of the two-way model “target = proxy source_*1*_ + proxy source_*2*_”, estimated admixture proportions ± 2 standard errors are between 0 and 1; 3) the *p*-value calculated by *qpAdm* for the two-way model ≥ 0.01.

For exploring performance of the distal rotating protocol, feasible two-way *qpAdm* models were simply filtered according to sampling dates of target groups and proxy sources. If target group’s sampling date was equal to or smaller than sampling dates of both proxy sources, such a model was considered distal.

### Non-rotating and model competition qpAdm protocols

In the non-rotating protocol, for each simulated admixture graph six oldest sampled groups were selected as a fixed “right” set (ties in sampling dates were resolved in alphabetical order; these “right” sets remained unchanged for a given topology across all independent simulations), and for the remaining seven groups all possible one-way and two-way admixture models were tested (105 models), applying the same composite feasibility criterion that was used above for the rotating protocol. This is the proximal non-rotating protocol, and alternatively we focused on distal admixture models only (distal non-rotating protocol).

In the proximal model competition protocol, subsequent analysis was focused on targets for whom two or more alternative *qpAdm* models emerged as feasible at the first step. For each target, alternative proxy sources were pooled and rotated between the “left” and “right” sets, testing only the models that emerged as feasible at the first step and applying the composite feasibility criterion (*p*-value 3 0.01, estimated admixture proportions ± 2 SE are between 0 and 1). Rotation of alternative proxy sources was performed in two alternative ways: “whatever is not on the left is on the right”, or placement of alternative sources in the “right” set one by one. In the latter case several “right” sets were tested for each model, and the model was considered supported by the model competition protocol only if it was not rejected under any of these “right” sets (the latter protocol follows Maróti et al. 2022). If only one model was feasible for a target, such a model was evaluated as passing the model competition procedure. A distal model competition protocol was not tested in this study.

For testing statistical significance of differences in FDR between *qpAdm* protocols, the following approach was used. FDR was calculated either on low-quality data for 10 random site/individual subsampling replicates derived from simulation replicate no. 1 (simulation setups no. 1 and 2) or on high-quality data for 10 independent simulation replicates (simulation setups no. 1 and 2). Comparisons of four *qpAdm* protocols (rotating and non-rotating, proximal and distal) were performed independently on these four sets of replicates, using the two-sided paired Wilcoxon test (Table 2). Comparisons of the same *qpAdm* protocols on lower and higher amounts of data (300-Mbp-sized vs. 1,000-Mbp-sized simulated genomes) were performed using the two-sided non-paired Wilcoxon test since simulation replicates were independent unlike alternative *qpAdm* protocols applied to the same data (Table 2).

### Classifying two-way admixture models into false and true positives

Since the simulated admixture graph topologies were complex and random, target groups modelled with *qpAdm* had very complex admixture history in some cases, being a part of gene flow networks. In this context it is hard to draw a strict boundary between true and false admixture models composed of a target and only two proxy sources. Two-way admixture models were considered false only if at least one of the following criteria was satisfied (considering only graph topologies and admixture proportions):

1. The target and at least one of the proxy sources are simulated as strictly cladal (Fig. 4, Fig. S4a). In this case the target may either be unadmixed, or it may have experienced gene flows earlier in its history that do not break its cladality with one of the proxy sources.
2. A proxy source lineage is a recipient of gene flow from the target lineage (after the last admixture event in target’s history), possibly mediated by other lineages (Fig. 4, Fig. S4a, b). In other words, the incorrect proxy source is a descendant of the target lineage, i.e., the expected gene flow direction is reversed.
3. A proxy source does not represent any true source. In other words, it is symmetrically related to all true sources of ancestry in the target (Fig. S4c, d). Alternatively, both proxy sources represent the same true source, and are symmetrically related to all the other true sources (Fig. S4e).
4. A proxy source shares genetic drift with the corresponding true source that is not shared by the second proxy source (and the same is true for the other proxy source and another true source, i.e., condition no. 3 above is not satisfied), however less than 40% of its ancestry is derived from the true source (Fig. S4a, d).
5. The target gets a gene flow from a deep-branching source not represented by any sampled population, and an inappropriate proxy source is included in the fitting model (Fig. S4f, g).

We illustrate these topological rules with eight case studies of FP and feasible *qpAdm* models in Fig. 4 and Fig. S4a–g. Two-way models for targets whose population history is best approximated with three-way and more complex models were considered as TP if they included source proxies (that do *not* satisfy the criteria above) for at least two of three or more true ancestry sources (see three case studies in Fig. S4k–m).

### Principal component analysis

PCA was performed for one simulation replicate per simulation setup. Only high-quality data was used, and all individuals sampled from a simulation were co-analyzed. Prior to the analysis, linked sites were pruned with *PLINK v.2.00a3LM* (Chang et al. 2015) using the following settings: window size, 2000 SNPs; window step, 100 SNPs; *r*^*2*^ threshold = 0.5 (argument “--indep-pairwise 2000 100 0.5”). PCA was also performed using *PLINK v.2.00a3LM* under default settings, calculating 10 PCs. Interactive three-dimensional plots visualizing PC1, PC2, and PC3 were made using the *plotly* R package. A two-way admixture model was considered supported by PCA if:

1. the target group (the center of the cluster of target individuals, to be precise) lay between the clusters of proxy source individuals on a straight line in the three-dimensional PC space;
2. or if it was located at a distance of no more than three target cluster diameters from that straight line connecting the proxy source clusters.

The second pattern was more common among both TP and FP two-way admixture models: 1.5 and 1.3 times, respectively (across all non-subsampled simulated datasets). This situation is expected since many targets represent three-way and more complex mixtures, and due to post-admixture genetic drift (Peter 2022).

### ADMIXTURE analysis

*ADMIXTURE* analysis was performed for one simulation replicate per simulation setup. Only high-quality data was used, and all individuals sampled from a simulation were co-analyzed. Prior to the analysis, linked sites were pruned with *PLINK v.2.00a3LM* (Chang et al. 2015) using the following settings: window size, 2000 SNPs; window step, 100 SNPs; *r*^*2*^ threshold = 0.5 (argument “--indep-pairwise 2000 100 0.5”). *ADMIXTURE v.1.3* (Alexander et al. 2009) was used in the unsupervised mode under the default settings. The algorithm was run on each SNP dataset only once, with the number of hypothetical ancestral populations (*K*) ranging from 3 to 10. This range was selected since the total number of populations in each simulated history was 13. A two-way admixture model was considered supported by *ADMIXTURE* analysis if:

1. for at least one *K*, at least 5 of 10 target individuals were modelled as a mixture of at least two ancestry components, with a minor ancestry component exceeding 2%;
2. typically, ancestry component *A* in the target group was shared with at least 5 individuals in proxy source 1, but not in proxy source 2, and ancestry component *B* was shared with at least 5 individuals in proxy source 2, but not in proxy source 1 (see examples in Fig. 4 and Fig. S4); in some cases, both components *A* and *B* were found in the proxy sources, but in different proportions;
3. if only one ancestry component in the target was shared with the two proxy sources, the model was considered unsupported;
4. ancestry components in the target that are absent in any of the sources were ignored since three-way and more complex admixture histories are common in the set of random admixture graphs explored here (see examples of such topologies in Fig. S4k–m);
5. ancestry components in a proxy source that are absent in the target were also ignored since a proxy source may not be fully cladal with the real source (see examples of such topologies in Fig. S4i, j, m).

These rules were designed to reproduce typical reasoning of an archaeogeneticist interpreting *ADMIXTURE* results. Observing a pattern of ancestry components in the target group and proxy sources compatible with the admixture model “target = proxy source_*1*_ + proxy source_*2*_” for one *K* value was enough for declaring that the model is supported by the *ADMIXTURE* analysis. This condition was motivated by an observation that models supported at one *K* value only were equally common among FP and TP *qpAdm* models (10% and 13%, respectively, across the four simulation setups). Models supported at four or more *K* values were more common among TP *qpAdm* models (3.3% of FP and 12.6% of TP models across the four simulation setups).

### Probability density curves for radiocarbon and calendar dates

Probability density curves for published radiocarbon and calendar dates were constructed in *OxCal v.4.4*. For calendar dates, we used the *C-Simulate* function in *OxCal v.4.4* for simulating normally distributed dating methods, taking the average calendar date as a median and the length of the timespan as a 95% confidence interval. For radiocarbon dates, we used calibration based on the IntCal20 calibration curve. Probability densities were summarized using the *Sum* function in *OxCal v.4.4* for each of the three groups of individuals, those included in the “left”, “right”, and “target” population sets in at least one of the published *qpAdm* models (Narasimhan et al. 2019, Lazaridis et al. 2022), and then plotted together.

## Supplementary Material

Supplement 1

Supplement 2

## Figures and Tables

**Table 1.** Assessing the effect of temporal stratification of targets and proxy sources on FDR of non-rotating and rotating *qpAdm* protocols and their combinations with PCA and *ADMIXTURE* analyses. Another kind of temporal stratification, stratification of the “right” and “left” sets, was a part of the non-rotating protocols, but not of the rotating ones. In the left half of the table, feasible two-way models are binned by model type (false or true positive) and *qpAdm* protocol (rotating or non-rotating), and for each bin its size (number of models) and fraction of models that are distal (magenta bars) are shown. FDR values are visualized by cell background color. In the right half of the table these bins are further subdivided into models supported/not supported (highlighted in green and red, respectively) by another method (PCA or *ADMIXTURE*). In the case of simulation setups no. 1 and 2, 10 simulation replicates and 10 subsampling replicates derived from simulation replicate no. 1 were generated, and median, minimal, and maximal values across the replicates are shown for sizes of the bins, for fractions of models that are distal, and for FDR.

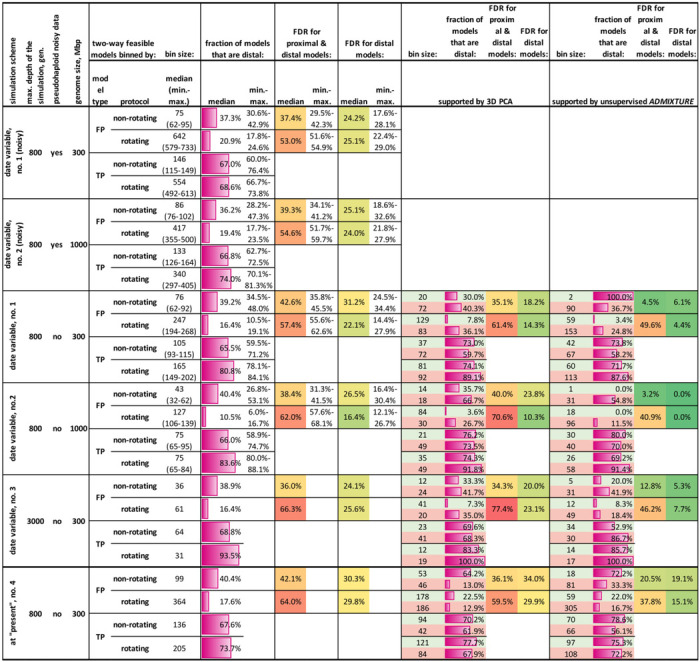

**Table 2.** Comparing FDR of four *qpAdm* protocols, proximal and distal, rotating and non-rotating, on 10 subsampling replicates derived from a single simulation replicate (low-quality data: top box) or on 10 simulation replicates and high-quality genomes (300-Mbp-sized or 1000-Mbp-sized genomes: bottom box). For details on generating replicates of low-quality data see Methods. Median FDR values for *qpAdm* protocols are shown on the diagonal and color-coded from largest (red) to smallest (green). Significance of FDR differences between each *qpAdm* analysis protocol was tested using the two-sided Wilcoxon test and the results are shown in the cells above the diagonal FDR values. For significance of FDR differences between 300-Mbp and 1000-Mbp-sized genomes see the top right cell diagonal under 1000-Mbp-sized genomes.

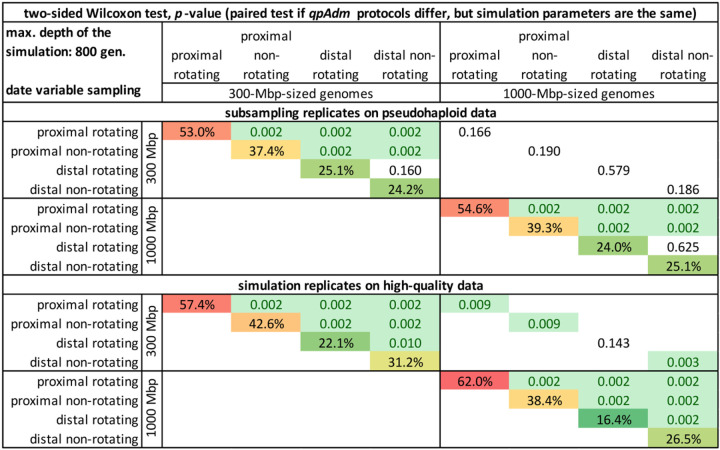

**Table 3.** Assessing FDR of the proximal non-rotating *qpAdm* protocol combined with model competition (Narasimhan et al. 2019). For each simulation setup, the analysis relies on simulation replicate no. 1 only. For constructing pipelines resembling those common in the archaeogenetic literature, we used five methods: proximal non-rotating *qpAdm*, *qpAdm* model competition (two alternative protocols), “admixture” *f*_*3*_-statistics, 3D PCA with all individuals co-analyzed, and unsupervised *ADMIXTURE* with all individuals co-analyzed. For declaring a positive result, support of an admixture model by all methods in the pipeline was required, hence the order of methods is not important except for the first method, which was the proximal non-rotating *qpAdm* in all cases. In each column, methods comprising a pipeline are color-coded and numbered by their order. All feasible two-way *qpAdm* models emerging as outcomes of the proximal non-rotating protocol were classified into FP and TP (highlighted in red and green in the leftmost column, respectively). The other columns are structured like bifurcating trees: FP *qpAdm* models supported by method no. 2; FP *qpAdm* models not supported by method no. 2; TP *qpAdm* models supported by method no. 2; TP *qpAdm* models not supported by method no. 2. The same principle is used for representing results of more complex pipelines. All model counts are normalized by the number of feasible *qpAdm* models (FP + TP) that are outcomes of the first method. Percentages of models supported/not supported by the last method in the pipeline are highlighted in green and red, respectively. FDR values are shown for these different pipelines. Fractions of TP *qpAdm* models that are pruned out by progressively more stringent support requirements are also shown (false omission rate or FOR).

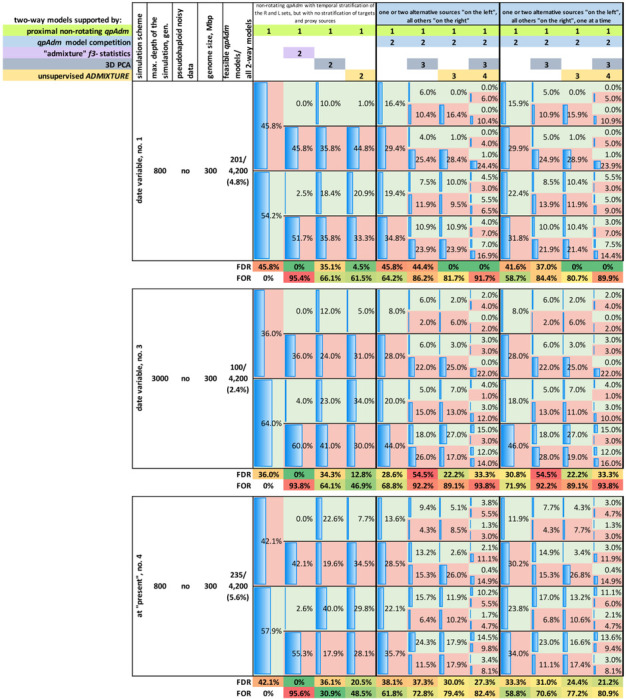

## Data Availability

The study is based on simulated data exclusively. **Suppl. dataset 1** contains summaries of 33,072 feasible two-way *qpAdm* models on which Table 2 and Fig. 6 are based; it is deposited at the GSA Figshare portal. Principal software packages used in the study are *ADMIXTOOLS 2* (Maier et al. 2023, https://github.com/uqrmaie1/admixtools) and *msprime v. 1.1.1* (Baumdicker et al. 2022, https://github.com/tskit-dev/msprime). For details on data simulations and computational analyses see lines 1313–1541 in this manuscript.

## Box 1. Terminology used in this study for describing admixture models, results of screens for admixture, and *qpAdm* protocols.

**Table:**

“right” (or outgroup) populations/groups	In a *qpAdm* protocol, these are reference populations needed for testing admixture models composed of a target and one or several proxy source groups.
target (or test) population/group	In an admixture model, this is a population/group whose genetic history is being modelled.
true (ancestry) source	In the context of simulated admixture-graph-shaped histories, this is a population directly participating in an admixture event(s) giving rise to a target group, and its ancestral (unsampled) population before its merging with other populations deeper in time.
proxy (ancestry) source	In the context of simulated admixture graphs, this is a sampled population included in an admixture model as a potential source and assumed to be cladal with one of true ancestry sources.
model complexity	Number of proposed proxy ancestry sources in an admixture model “target = proxy source_*1*_ + proxy source_*2*_ + … proxy source_*i*_”.
“left” populations/groups	In a *qpAdm* model, these are proxy sources and a target.
reference populations/groups	“Right” and proxy source populations combined.
(composite) model feasibility criterion	A criterion for identifying fitting (feasible) *qpAdm* models that relies on both estimated admixture proportions and p-values. The following criterion was used in this study (all the conditions listed below should be satisfied): 1) p-values for all models with *n—1* ancestry sources, such as one-way models “target = proxy source_*1*_’, “target = proxy source_*2*_, and “proxy source_*1*_ = proxy source_*2*_, are below 0.01; 2) for a model with *n* ancestry sources, such as a two-way model “target = proxy source_*1*_ + proxy source_*2*_, estimated admixture proportions ± 2 standard errors are between 0 and 1; 3) the p-value for a model with *n* ancestry sources > 0.01. Other versions of this criterion are also found in the literature: with different values of the p-value cut-off (usually 0.05 or 0.01) and/or imposing limits on estimated admixture proportions, but not considering their standard errors.
feasible (or fitting) *qpAdm* model	A *qpAdm* model satisfying the feasibility criterion above.
positive/negative admixture model	An admixture model supported/not supported by one or several analytical tools such as *qpAdm,* PCA, *ADMIXTURE*.
false discovery rate (FDR)	The fraction of admixture models of a certain complexity (e.g., two-way) satisfying the *qpAdm* model feasibility criteria but classified as false considering the simulated graph topology and simulated admixture proportions. For topological criteria used for classifying two-way admixture models into true and false ones see the Results and Methods sections. The term FDR is also applied to outcomes of more complex admixture inference pipelines composed of several methods.
false omission rate (FOR)	The fraction of feasible *qpAdm* models that are classified as true considering the simulated graph topology and simulated admixture proportions but are not supported by another method *(qpAdm* model competition, PCA, or *ADMIXTURE)* or a combination of methods.
“rotating” *qpAdm* protocol	A protocol having the following feature: a large subset of reference populations or all of them are distributed between the “right” and “left” sets according to the principle “whatever is not in the left set is in the right set”, testing all possible bisections of this sort for a given rotated set and a given range of model complexities. In the most extreme case, target groups are also included in this rotation. Model testing starts from the simplest one-way models and moves on to the next complexity level if all simple models for a given target are rejected according to the composite feasibility criterion. The goal of this approach, as compared to “non-rotating” protocols, is to increase the power of the method to reject non-optimal proxy ancestry sources.
“non-rotating” *qpAdm* protocol	This protocol we also term “standard” or “basic”: all models are tested with one or few fixed sets of “right” groups which usually pre-date “left” groups or are contemporaneous with them. In practice, modern populations genetically divergent from the target are often included in such a “right” set if ancient reference groups are unavailable. Model testing starts from the simplest one-way models and moves on to the next complexity level if all simple models for a given target are rejected according to the composite feasibility criterion.
temporal stratification of targets and proxy sources	A requirement that target groups post-date or are contemporaneous with all proxy sources in each model. In practice, this requirement is often included in rotating and nonrotating *qpAdm* protocols.
“distal” *qpAdm* protocol	A rotating or non-rotating *qpAdm* protocol with temporal stratification of targets and proxy sources.
“proximal” *qpAdm* protocol	A rotating or non-rotating *qpAdm* protocol without temporal stratification of targets and proxy sources.
model competition	A *qpAdm* protocol which starts from sets of alternative feasible *qpAdm* models of a certain complexity level for a given target, outcomes of a non-rotating protocol. Alternative proxy sources from these models form a rotated set. Rotation can be performed in two ways (groups from the rotated set are placed in the “left” set one by one, and the other groups from the rotated set are placed in the “right” set; or groups from the rotated set are placed in both “left” and “right” sets one by one), and the composite feasibility criterion is applied. The goal of this approach is to increase the power of the method to reject non- optimal proxy ancestry sources.
proximal model competition *qpAdm* protocol	As introduced by Narasimhan *et al*. (2019), this is a non-rotating *qpAdm* protocol with temporal stratification of “right” and “left” sets, with a subsequent model competition step, and with no (or very limited) temporal stratification of targets and proxy sources at both steps.
