# Performance of *qpAdm* -based screens for genetic admixture on admixture-graph-shaped histories and stepping-stone landscapes

**DOI:** 10.1101/2023.04.25.538339

**Published:** 2024-09-03

**Authors:** Olga Flegontova, Ulaş Işıldak, Eren Yüncü, Matthew P. Williams, Christian D. Huber, Jan Kočí, Leonid A. Vyazov, Piya Changmai, Pavel Flegontov

## Abstract

*qpAdm* is a statistical tool that is often used in exploratory archaeogenetic studies for finding optimal admixture models of population history. Despite its popularity, *qpAdm* remains untested on histories in the form of admixture graphs of random topology or stepping-stone landscapes. We analyzed data from such simulations and found that while for admixture-graph-shaped histories there exist simple solutions (temporal stratification) for minimizing false findings of gene flow, in the case of stepping-stone landscapes the method generates results that do not appear suspect but are misleading: feasible *qpAdm* models are either accurate but simplistic in the context of landscapes, or highly inaccurate in the case of multi-component models. This is largely is due to two reasons: 1) because of complex migration networks that violate the assumptions of the method, there is poor correlation between *qpAdm p* -values and model optimality in many sections of the parameter space; 2) admixture fraction estimates between 0 and 1 are largely restricted to symmetric source configurations around targets, hence popular [0, 1] model plausibility criteria confound analyses of landscape-type demographies, unless their interpretations are explicitly spatial. For many species/regions/periods archaeogenetic sampling is very sparse and may be random with respect to population density of ancient individuals. In this situation only a specific combination of landscape properties and feasibility criteria allows to efficiently reject highly asymmetric non-optimal models most abundant in random deme sets. This problem may obscure useful signal (rare optimal models) and might be responsible for some claims about rapid long-distance migrations in the literature.

## Full Text Availability

The license terms selected by the author(s) for this preprint version do not permit archiving in PMC. The full text is available from the preprint server.

