# Supplementary material for: False discovery rates of qpAdm-based screens for genetic admixture
Source: bioRxiv. 2023 Oct 18:2023.04.25.538339. Preprint. [Version 2] doi: 10.1101/2023.04.25.538339 (PMC10614728; doi:10.1101/2023.04.25.538339)
Supplement: Supplementary file 1 [file media-2.pdf]

Supplementary text 1

There is no practical way of reducing FDR of the proximal rotating qpAdm protocol

We observe that among two-way admixture models supported by both proximal rotating *qpAdm* and PCA, FDR remains very high: from 60% to 77%, and this comes at a cost of rejecting 41% or 61% of TP *qpAdm* models (false omission rate, FOR) as those were not supported by PCA (**Table S1**). Adding an unsupervised *ADMIXTURE* analysis applied to all 13 simulated groups as another level of control helped to drive FDR below 50% (to 42%) only in the case of the fourth simulation setup where all groups were sampled at "present" (**Table S1**). Considering only admixture models supported by all three methods increases FOR to 69 – 76% (**Table S1**).

There exists a test for admixture that helped to reduce the FDR of the proximal rotating *qpAdm* protocol more substantially than PCA or *ADMIXTURE*, to 14 – 33% on high-quality data (**Table S1**), and these are "admixture" f_3-statistics of the form f_3(target; proxy source$_1$, proxy source$_2$) (Patterson et al. 2012). FDR of models supported by f_3-statistics not combined with *qpAdm* was even lower, 6% (7 of 115 statistics with Z-score < -2 in the case of at least one of four sets of simulated high-quality data; simulation replicate no. 1 was considered for each simulation setup). However, "admixture" f_3-statistics tend to become positive when genetic drift on the target lineage moves allele frequencies away from their original values intermediate between those in the real ancestry sources (Patterson et al. 2012). Indeed, FOR for models supported by both *qpAdm* and f_3-statistics varied from 93% to 100% (**Table S1**), and just 115 of 34,320 population triplets tested with f_3-statistics demonstrated Z-scores < -2 under at least one simulation setup (one replicate per setup considered). Thus, using f_3-statistics is impractical in large-scale screens for admixture.

Moreover, in the case of one simulated history, we found a class of false two-way admixture models consistently supported by negative f_3-statistic Z-scores down to -18.4 (on 1000-Mbp-sized genomes and high-quality data), and also having strong support by *qpAdm*, PCA, and *ADMIXTURE* (**Fig. S4f**). In this case the target receives a gene flow from the most divergent lineage that remains unsampled, and other deep-branching lineages unrelated to the true source emerge as proxy sources in fitting *qpAdm* models (**Fig. S4f**). f_4-statistics are defined on

unrooted trees, and f_3-statistics, being just a special case of them (Patterson et al. 2012), have the same property, which explains our observation.

Table S1. Assessing FDR of the proximal rotating *qpAdm* protocol and of more complex admixture inference pipelines based on it. For each simulation setup, the analysis relies on simulation replicate no. 1 only since PCA and *ADMIXTURE* results were not generated for multiple replicates (see Discussion). For constructing the pipelines, we used three additional methods: "admixture" f_3-statistics, 3D PCA with all individuals co-analysed, and unsupervised *ADMIXTURE* with all individuals co-analysed. In each pipeline, for declaring a positive result support of an admixture model by all methods was required, hence the order of methods is not important except for the first method, which was proximal rotating *qpAdm* in all cases. In each column, methods comprising a pipeline are color-coded and numbered by their order. All feasible two-way *qpAdm* models emerging as outcomes of the proximal rotating protocol were classified into FP and TP (highlighted in red and green in the leftmost column, respectively). The other columns are structured like bifurcating trees: FP *qpAdm* models supported by method no. 2; FP *qpAdm* models not supported by method no. 2; TP *qpAdm* models supported by method no. 2; TP *qpAdm* models not supported by method no. 2. The same principle is used for representing results of more complex pipelines. All model counts are normalized by the number of feasible *qpAdm* models (FP + TP), outcomes of the first method. Percentages of models supported/not supported by the last method in the pipeline are highlighted in green and red, respectively. FDR values are shown for these different pipelines. Fractions of TP *qpAdm* models that are pruned out by progressively more stringent support requirements are also shown (false omission rate or FOR).

simulation scheme	max. depth of the simulation, gen.	pseudohaploid noisy data	genome size, Mbp	feasible *qpAdm* models/ all 2-way models	two-way models supported by:				
					proximal rotating *qpAdm*				
					"admixture" *f3*- statistics				
					3D PCA				
					unsupervised *ADMIXTURE*				
					1	1	1	1	1
						2			
							2		2
								2	3
date variable, no. 1	800	no	300	385/ 34,320 (1.1%)	55.1%	0.5%	33.5%	15.3%	10.9%
									22.6%
						54.5%	21.6%	39.7%	4.4%
									17.1%
					44.9%	2.1%	21.0%	15.6%	10.6%
									10.4%
						42.9%	23.9%	29.4%	4.9%
									19.0%
				FDR	55.1%	20.0%	61.4%	49.6%	50.6%
				FOR	0%	95.4%	53.2%	65.3%	76.3%
date variable (no. 2)	800	no	1000	198/ 34,320 (0.6%)	57.6%	0.5%	42.4%	9.1%	8.1%
									34.3%
						57.1%	15.2%	48.5%	1.0%
									14.1%
					42.4%	3.0%	17.7%	13.1%	10.1%
									7.6%
						39.4%	24.7%	29.3%	3.0%
									21.7%
				FDR	57.6%	14.3%	70.6%	40.9%	44.4%
				FOR	0%	92.9%	58.3%	69.0%	76.2%
date variable, no. 3	3000	no	300	92/ 34,320 (0.3%)	66.3%	1.1%	44.6%	13.0%	13.0%
									31.5%
						65.2%	21.7%	53.3%	0.0%
									21.7%
					33.7%	2.2%	13.0%	15.2%	8.7%
									4.3%
						31.5%	20.7%	18.5%	6.5%
									14.1%
				FDR	66.3%	33.3%	77.4%	46.2%	60.0%
				FOR	0%	93.5%	61.3%	54.8%	74.2%
at "present", no. 4	800	no	300	569/ 34,320 (1.7%)	64.0%	0.4%	31.3%	10.4%	7.9%
									23.4%
						63.6%	32.7%	53.6%	2.5%
									30.2%
					36.0%	0.0%	21.3%	17.0%	11.1%
									10.2%
						36.0%	14.8%	19.0%	6.0%
									8.8%
				FDR	64.0%	N/A	59.5%	37.8%	41.7%
				FOR	0%	100.0%	41.0%	52.7%	69.3%

Another way of reducing FDR of the proximal rotating *qpAdm* protocol is adjustment of *p*-value thresholds. All published rotating *qpAdm* protocols, to our knowledge, rely on one *p*-value threshold (usually 0.01 or 0.05), both for rejection of simple models and for acceptance of more complex models with an additional ancestry source. Here we compare distributions of *p*-values for FP and TP two-way models and the corresponding one-way models (we note that the distributions are conditional on the admixture graphs simulated). The means of the distributions of log_{10}(*p*-values) for one-way models were significantly different for TP and FP two-way models, although the distributions largely overlap (**Fig. S6b**; a one-way model with the highest *p*-value was considered for each population triplet). On high-quality diploid data, average log_{10}(p-values) for two-way models were significantly different for true and false *qpAdm* models only in the case of the fourth simulation setup (with groups sampled at "present") (**Fig. S6b**).

		0.01				0.1				***p*-value theshold for 2-way models**
**simulation setup**	**model supported by:**	**0.01**	**1.E-10**	**1.E-20**	**1.E-50**	**0.01**	**1.E-10**	**1.E-20**	**1.E-50**	***p*-value theshold for 1-way models**
sampling dates variable, 1,000 Mbp	*qpAdm*	57.6%	46.2%	45.5%	44.1%	54.9%	44.1%	44.3%	38.0%	
sampling dates variable, 300 Mbp, noisy data	*qpAdm*	53.3%	44.1%	41.1%	38.7%	48.0%	37.4%	33.6%	32.6%	
sampling dates variable, 300 Mbp	*qpAdm*	55.1%	50.2%	47.6%	43.7%	51.9%	44.4%	41.1%	33.3%	
	qpAdm + PCA	61.4%	53.8%	49.3%	43.6%	60.0%	51.3%	47.8%	41.5%	
	qpAdm + PCA + *ADMIXTURE*	50.6%	43.7%	37.7%	35.4%	50.0%	42.1%	36.4%	32.1%	**false discovery rate (FDR)**
sampling dates variable, deep divergence, 300 Mbp	*qpAdm*	66.3%	58.7%	58.6%	58.7%	68.6%	59.3%	60.0%	59.1%	
	qpAdm + PCA	77.4%	72.7%	72.1%	71.1%	73.1%	68.2%	66.7%	63.2%	
	qpAdm + PCA + *ADMIXTURE*	60.0%	57.9%	55.6%	50.0%	50.0%	50.0%	44.4%	28.6%	
sampled at "present", 300 Mbp	*qpAdm*	64.0%	56.8%	50.8%	50.5%	55.9%	48.8%	44.5%	39.0%	
	qpAdm + PCA	59.5%	52.9%	49.2%	44.1%	53.2%	45.1%	43.4%	32.2%	
	qpAdm + PCA + *ADMIXTURE*	41.7%	36.9%	35.4%	37.3%	36.1%	30.0%	29.8%	26.7%	
sampling dates variable, 1,000 Mbp	*qpAdm*	0.0%	8.3%	14.3%	21.4%	51.2%	54.8%	59.5%	63.1%	
sampling dates variable, 300 Mbp, noisy data	*qpAdm*	0.0%	37.8%	53.6%	75.0%	46.6%	66.7%	74.8%	86.7%	
sampling dates variable, 300 Mbp	*qpAdm*	0.0%	15.0%	24.3%	35.8%	48.0%	57.2%	61.8%	66.5%	
	qpAdm + PCA	53.2%	57.2%	60.1%	67.1%	74.6%	77.5%	79.2%	82.1%	
	qpAdm + PCA + *ADMIXTURE*	76.3%	76.9%	78.0%	82.1%	86.7%	87.3%	87.9%	89.0%	
sampling dates variable, deep divergence, 300 Mbp	*qpAdm*	0.0%	0.0%	6.5%	16.1%	64.5%	64.5%	67.7%	71.0%	**false omission rate (FOR)**
	qpAdm + PCA	61.3%	61.3%	61.3%	64.5%	77.4%	77.4%	77.4%	77.4%	
	qpAdm + PCA + *ADMIXTURE*	74.2%	74.2%	74.2%	74.2%	83.9%	83.9%	83.9%	83.9%	
sampled at "present", 300 Mbp	*qpAdm*	0.0%	18.0%	24.4%	50.2%	38.5%	48.8%	53.2%	68.8%	
	qpAdm + PCA	41.0%	51.7%	54.6%	67.8%	64.4%	69.8%	72.7%	80.5%	
	qpAdm + PCA + *ADMIXTURE*	69.3%	74.1%	75.1%	84.4%	81.0%	82.9%	83.9%	89.3%	

Table S2. False discovery and false omission rates (FDR, FOR) of the proximal rotating *qpAdm* protocol for a range of model positivity criteria. Simulation setups and methods used to declare a positive result are shown on the left. Two *p*-value thresholds were tested for accepting two-way models (0.01 and 0.1), and four *p*-value thresholds were tested for rejecting one-way models (0.01, 10^{-10}, 10^{-20}, and 10^{-50}). For each simulation setup, the analysis relies on simulation replicate no. 1 only since PCA and *ADMIXTURE* results were not generated for multiple replicates (see Discussion).

These *p*-value distributions (**Fig. S6**) suggest that introducing different thresholds for rejecting one-way *qpAdm* models and for accepting two-way models may reduce FDR. Indeed, setting the former threshold to 10^{-50} and the latter to 0.1 reduces FDR from 55% – 66% to 33% – 59% on high-quality data, with FOR from 63% to 71% (**Table S2**). FDR can be reduced even further, down to 27%, if support by proximal rotating *qpAdm*, PCA and *ADMIXTURE* is required to

declare a positive result. This approximately two-fold reduction in FDR is associated with a very high FOR of up to 89% (**Table S2**).

In summary, we conclude that without the introduction of temporal stratification, on our simulated data it was impossible to reduce FDR of the proximal rotating *qpAdm* protocol even below 20%.

Figure. S1.

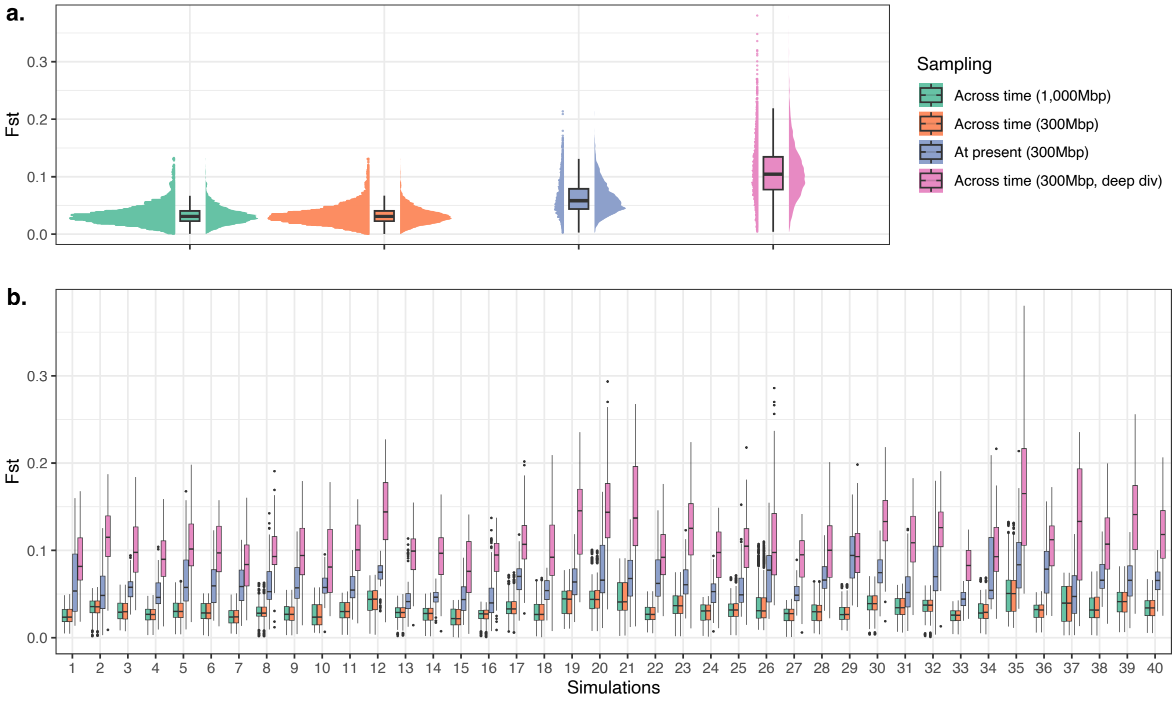

F_{ST} distributions for four sets of simulations: 1 & 2) populations sampled at different points in time and with maximal graph depth at 800 generations, 1,000-Mbp-sized or 300-Mbp-sized genomes, 10 simulation replicates per topology (in green and orange, respectively); 3) all populations sampled at "present" and with maximal graph depth at 800 generations, 300-Mbp-sized genomes, one simulation replicate per topology (in blue); 4) and populations sampled at different points in time and with maximal graph depth at 3,000 generations, 300-Mbp-sized genomes, one simulation replicate per topology (in magenta). In all cases, diploid data were used. The distributions are shown either for all 40 topologies pooled (**a**), or for each topology separately (**b**).

Figure S2.

a

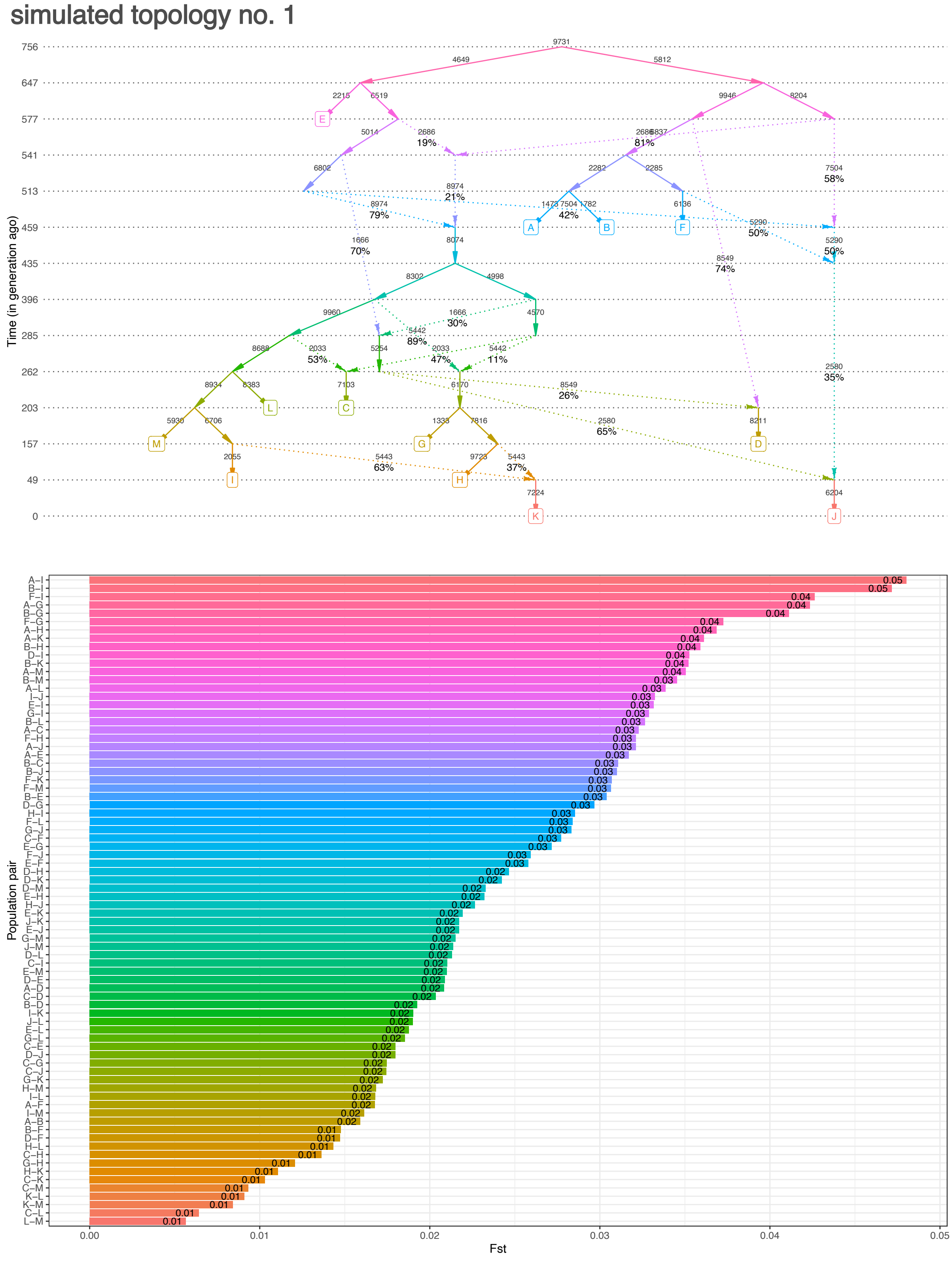

simulated topology no. 1
Time (in generation ago)
756
647
577
541
513
459
435
396
285
262
203
157
49
0
A
B
C
D
E
F
G
H
I
J
K
L
M
Population pair
Fst
0.00
0.01
0.02
0.03
0.04
0.05

b

simulated topology no. 1

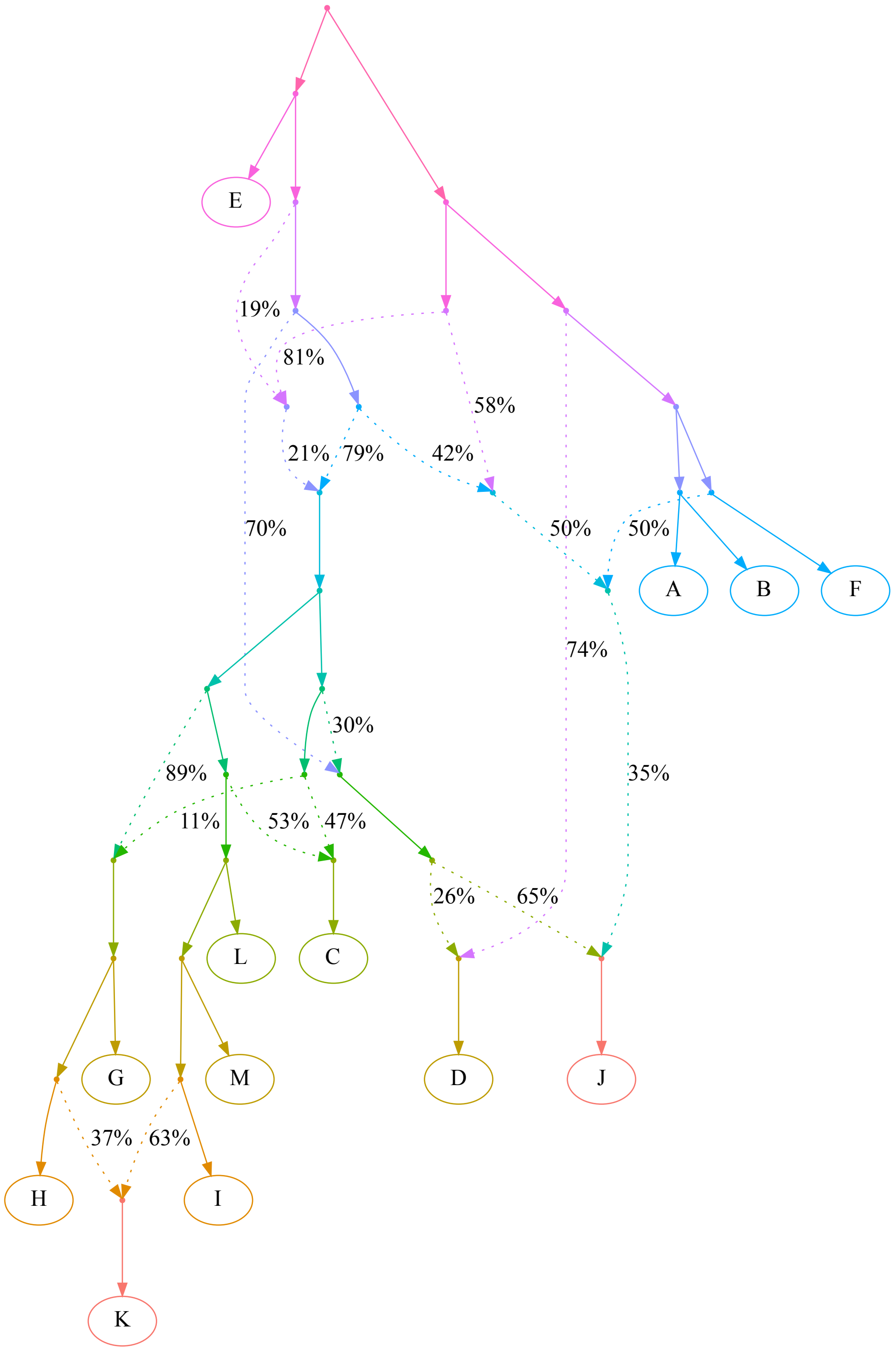

c

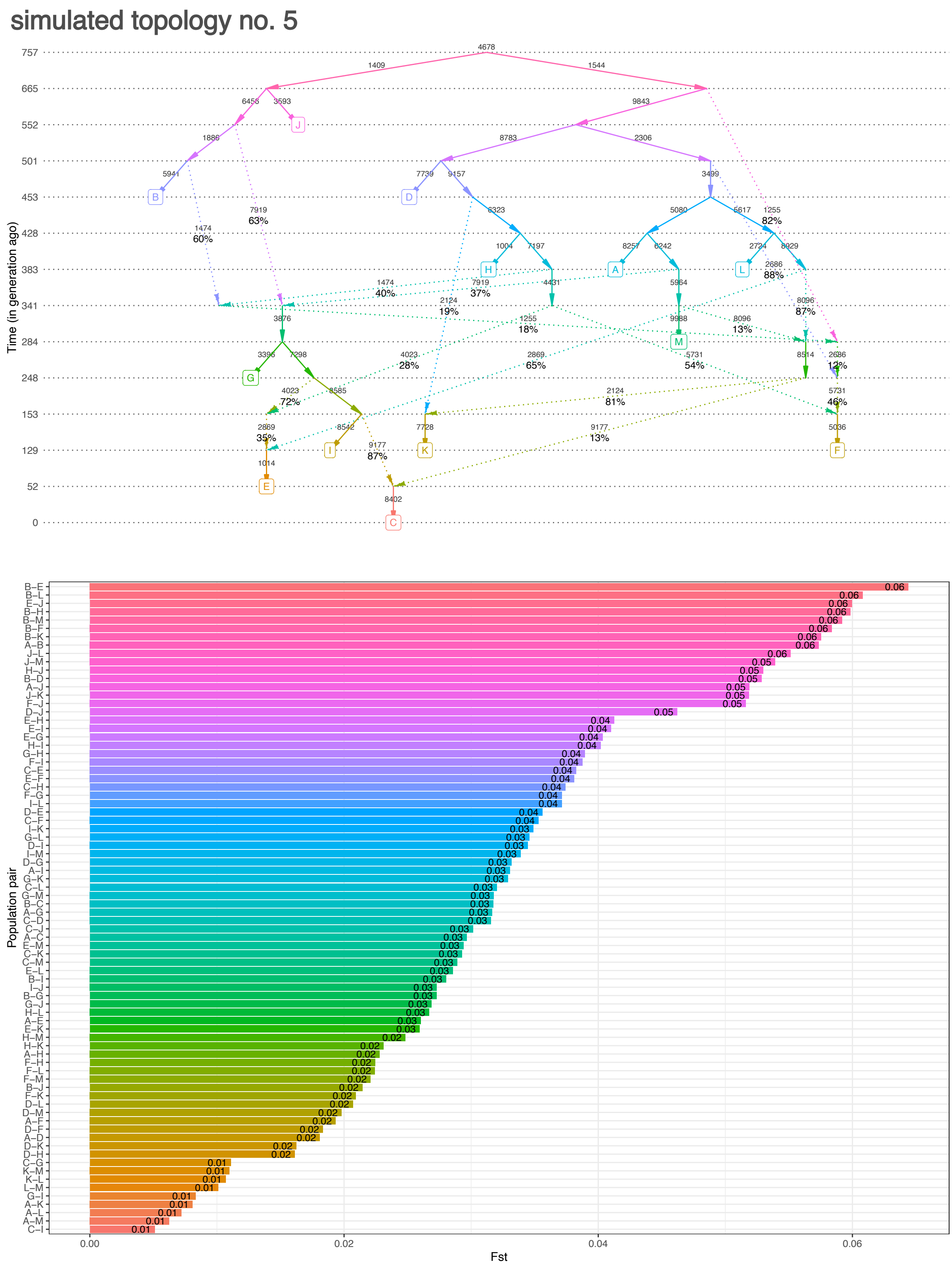
simulated topology no. 5
Time (in generation ago)
757
665
552
501
453
428
383
341
284
248
153
129
52
0
B
J
D
H
A
L
M
G
I
K
F
E
C
Population pair
B–E
B–L
E–J
B–H
B–M
B–F
B–K
A–B
J–L
J–M
H–J
B–D
A–J
J–K
F–J
D–J
E–H
E–I
E–G
H–I
G–H
F–I
C–E
E–F
C–H
F–G
I–L
D–E
C–F
I–K
G–L
D–I
I–M
D–G
A–I
G–K
C–L
G–M
B–C
A–G
C–D
C–J
A–C
E–M
E–K
C–M
E–L
B–I
I–J
B–G
G–J
H–L
A–E
E–K
H–M
H–K
A–H
F–H
F–L
F–M
B–J
F–K
D–L
D–M
A–F
D–F
A–D
D–K
D–H
C–G
K–M
K–L
L–M
G–I
A–K
A–L
A–M
C–I
0.00
0.02
0.04
0.06
Fst

d

simulated topology no. 5

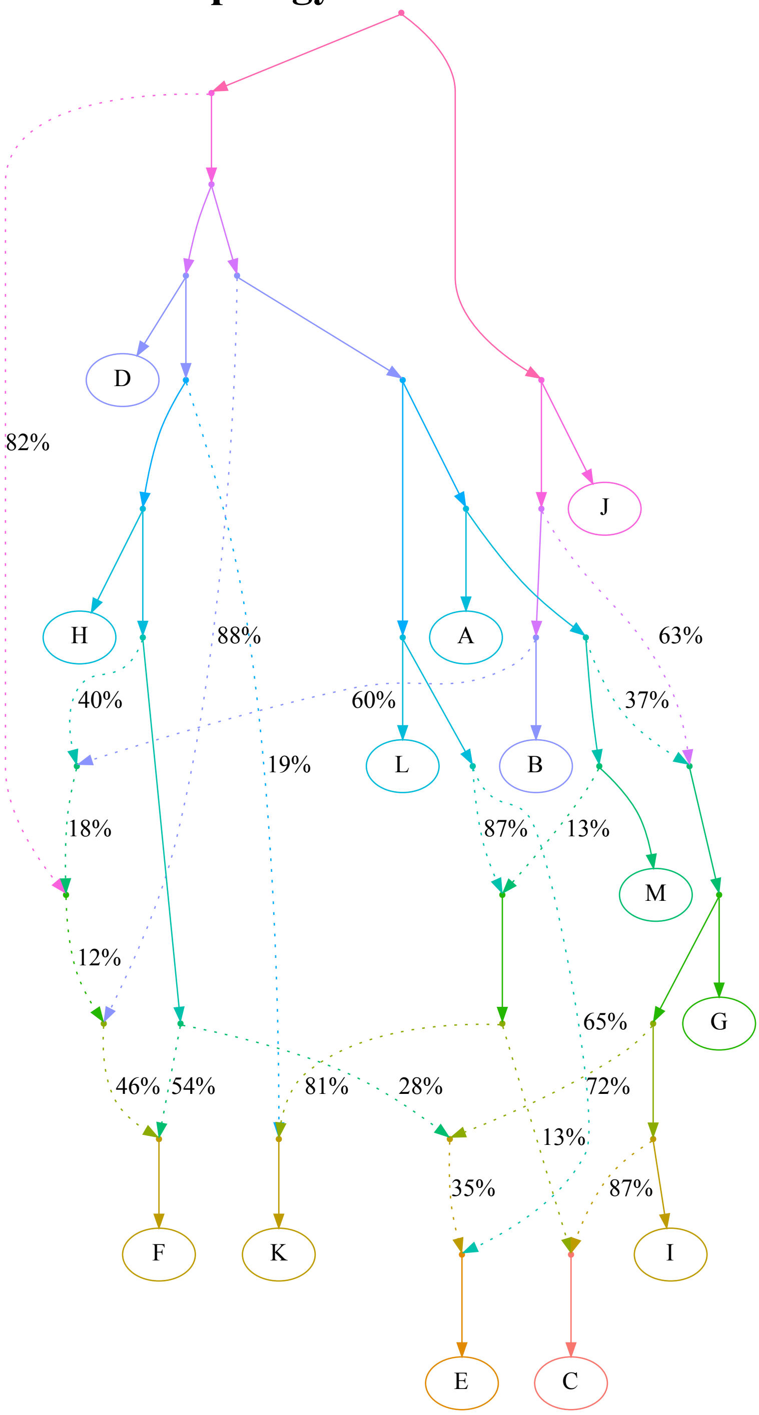

e

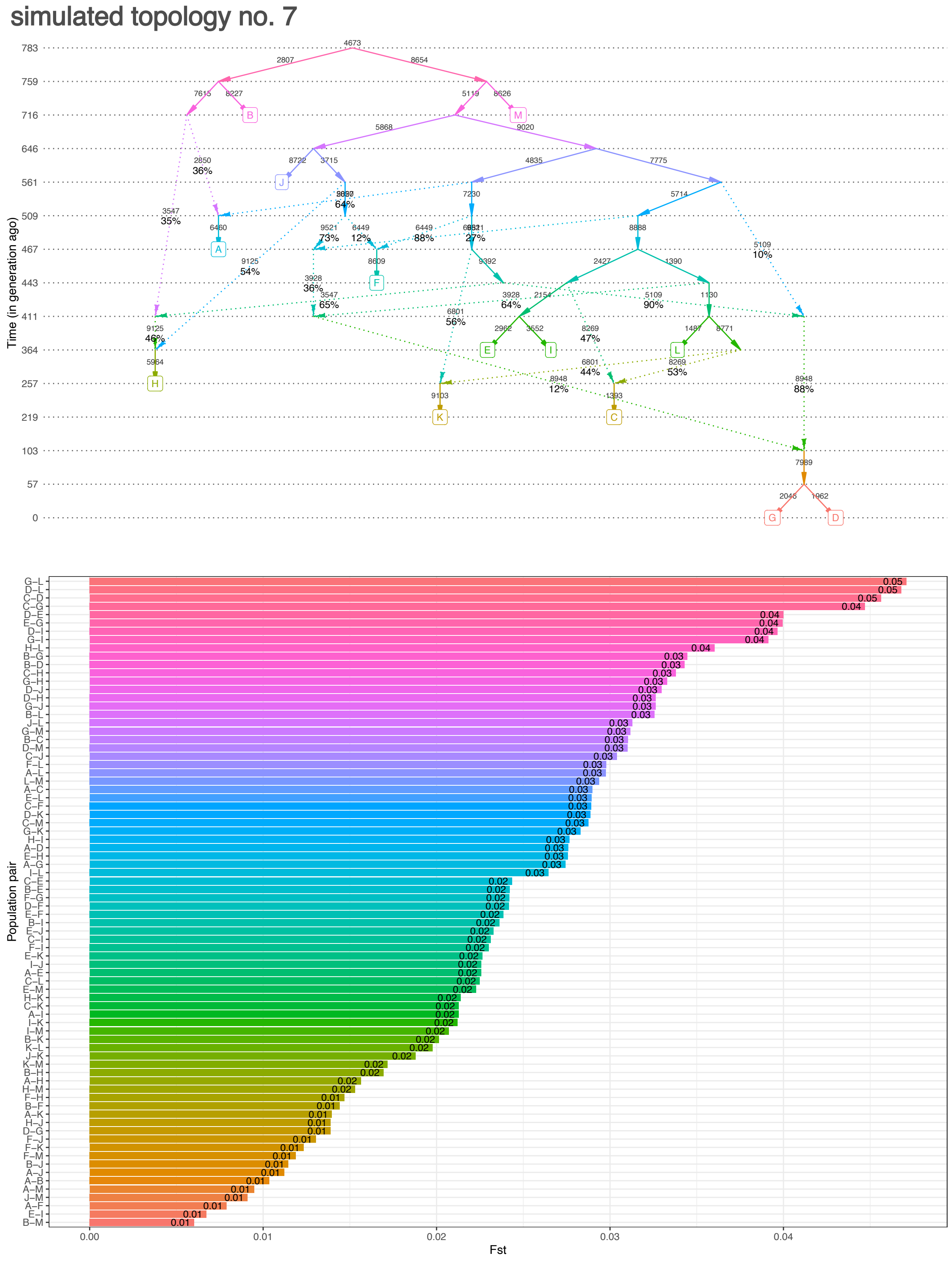

simulated topology no. 7
Time (in generation ago)
783
759
716
646
561
509
467
443
411
364
257
219
103
57
0
Population pair
Fst
0.00
0.01
0.02
0.03
0.04

f

simulated topology no. 7

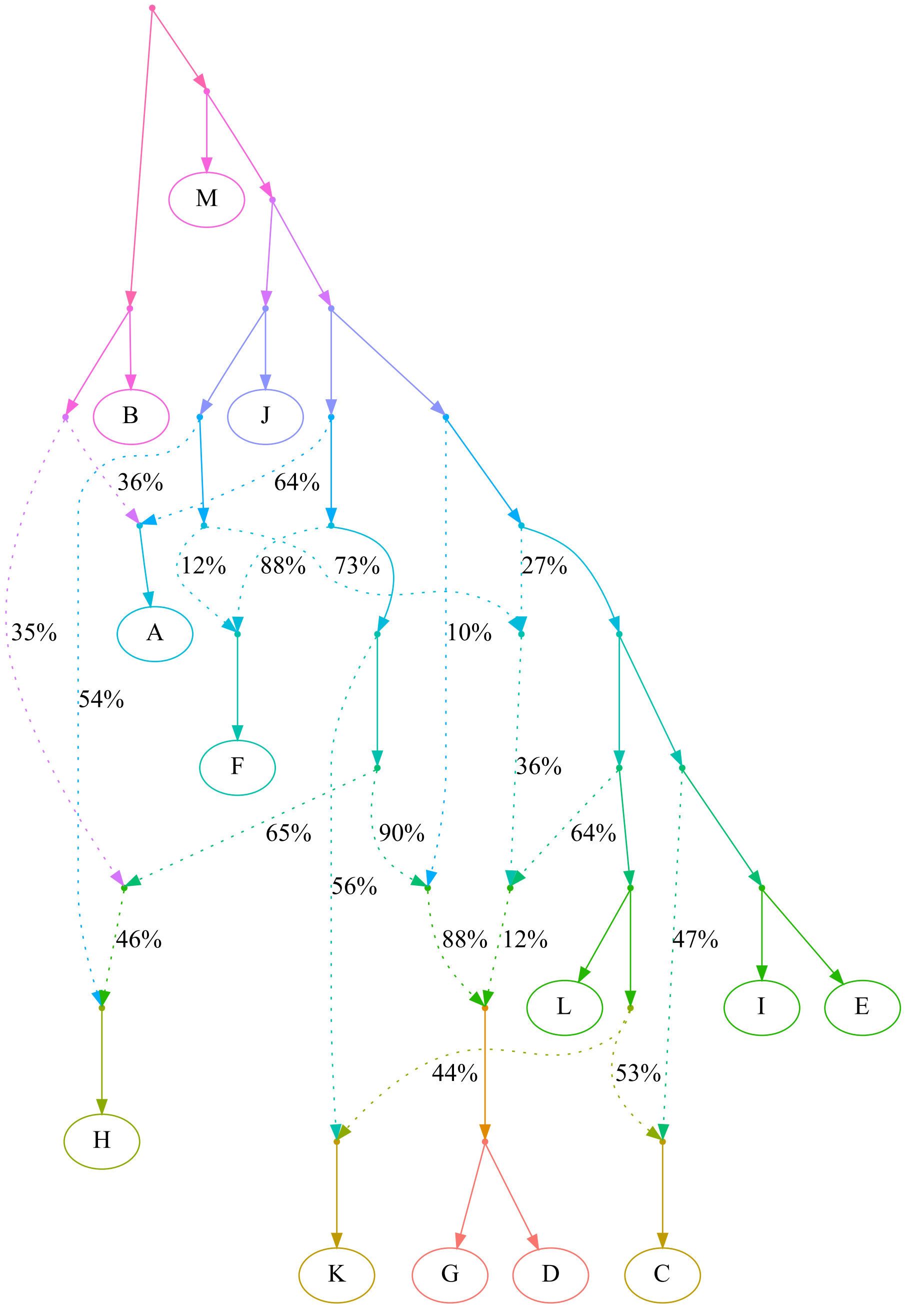

g

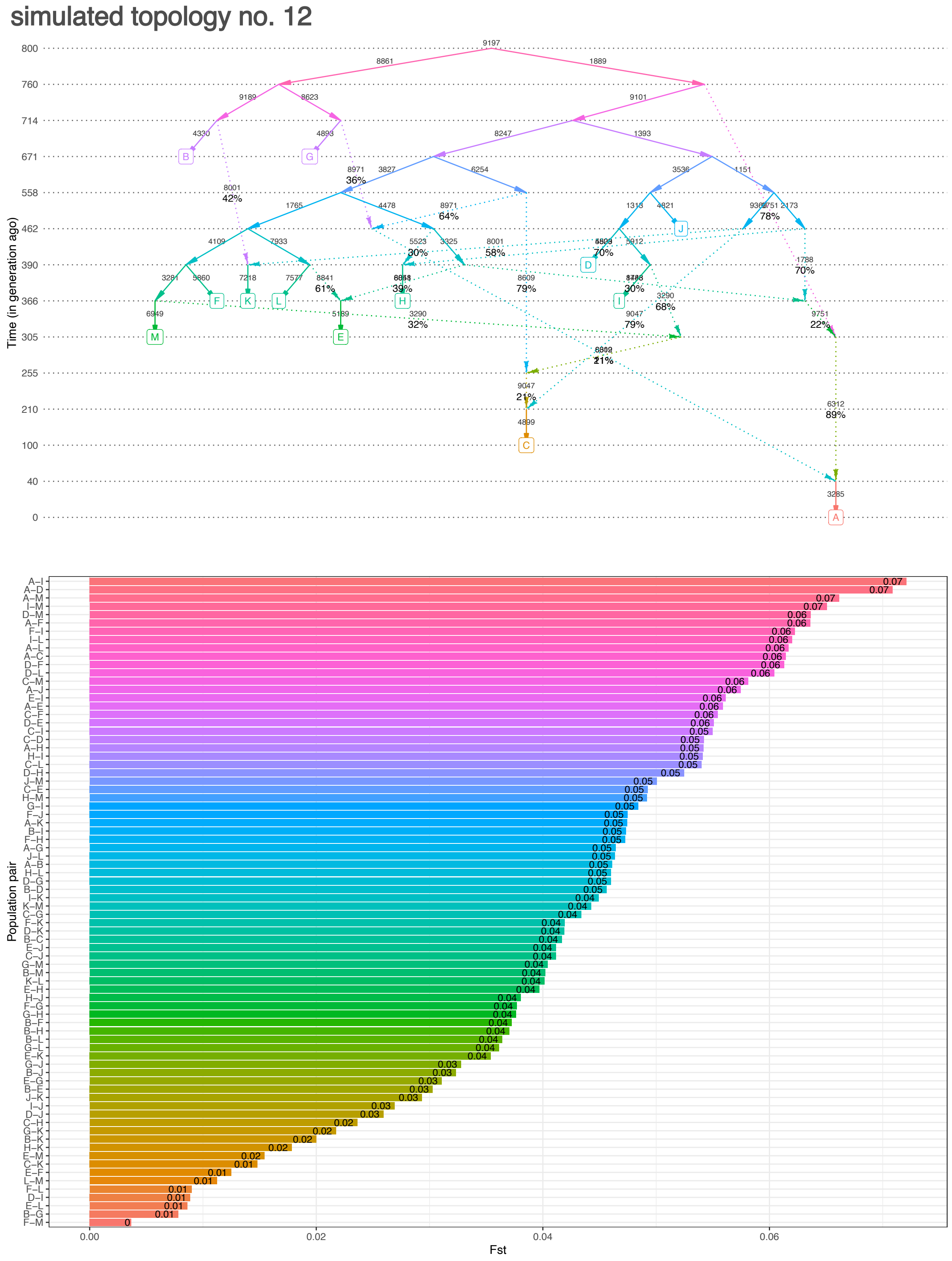
simulated topology no. 12
Time (in generation ago)
800
760
714
671
558
462
390
366
305
255
210
100
40
0
B
G
J
D
F
K
L
H
I
M
E
C
A
Population pair
Fst
0.00
0.02
0.04
0.06

h

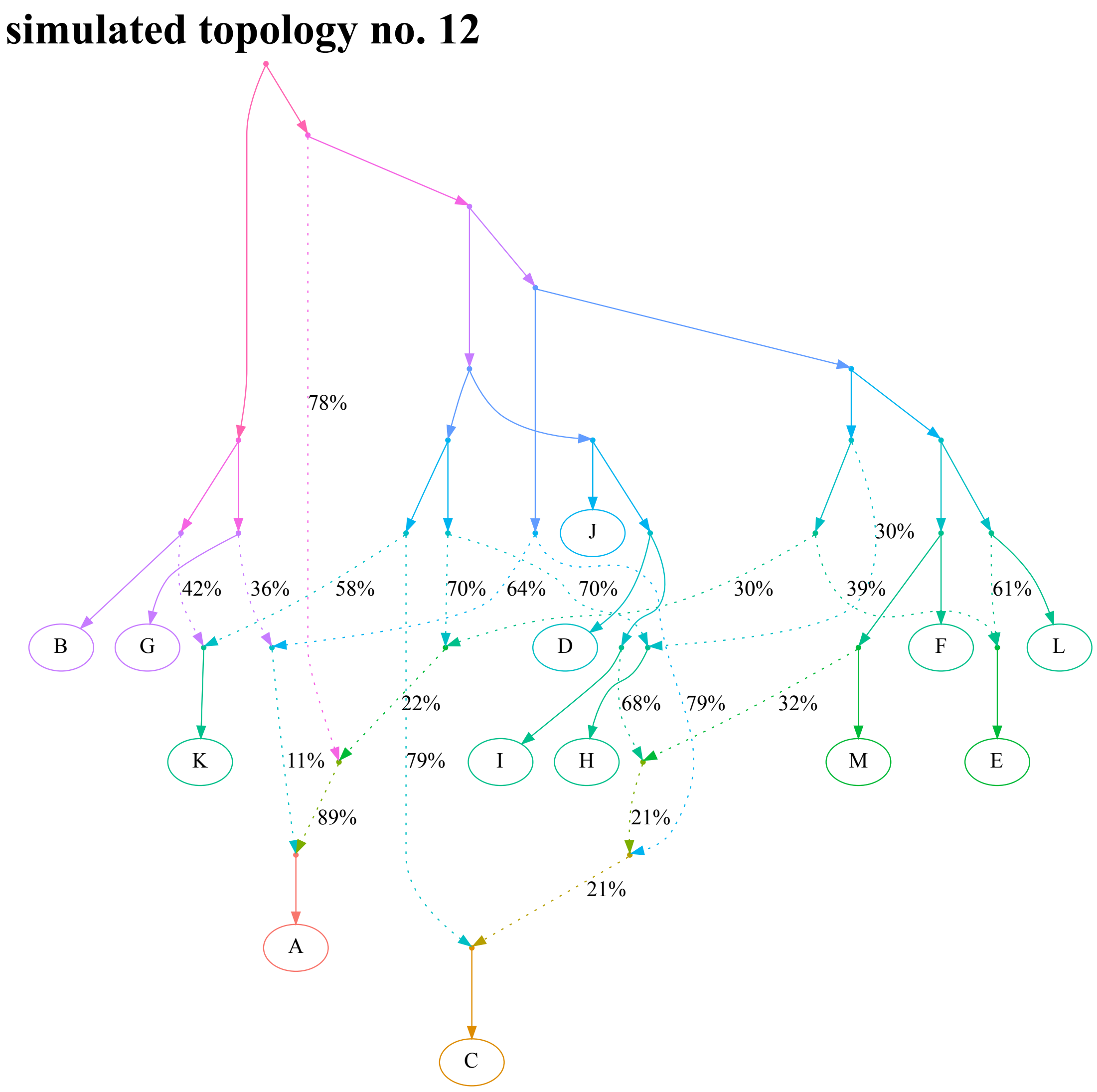
simulated topology no. 12
78%
42%
36%
58%
70%
64%
70%
30%
30%
39%
61%
22%
68%
79%
32%
11%
79%
89%
21%
21%
A
B
C
D
E
F
G
H
I
J
K
L
M

i

simulated topology no. 17

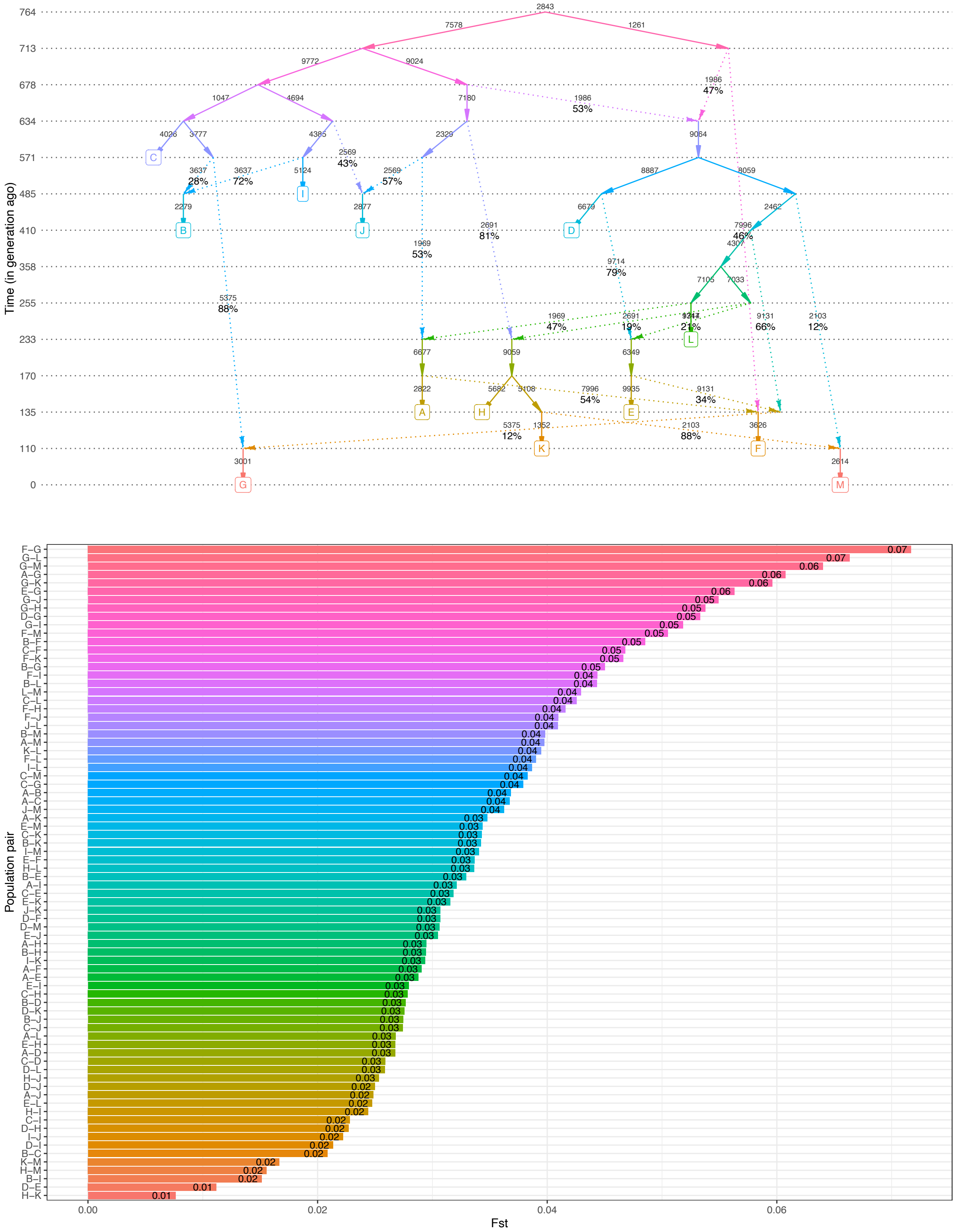

j

simulated topology no. 17

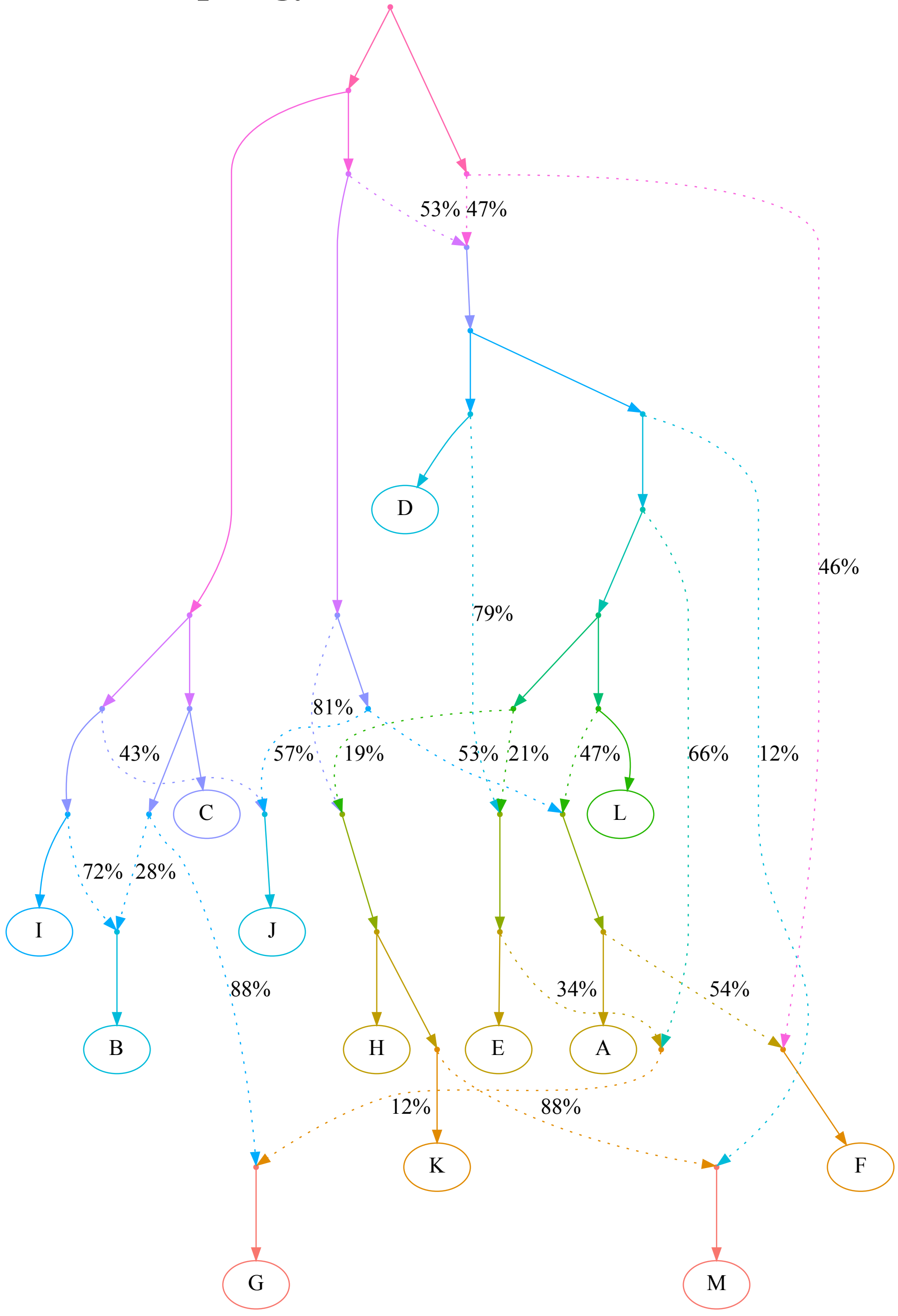

k

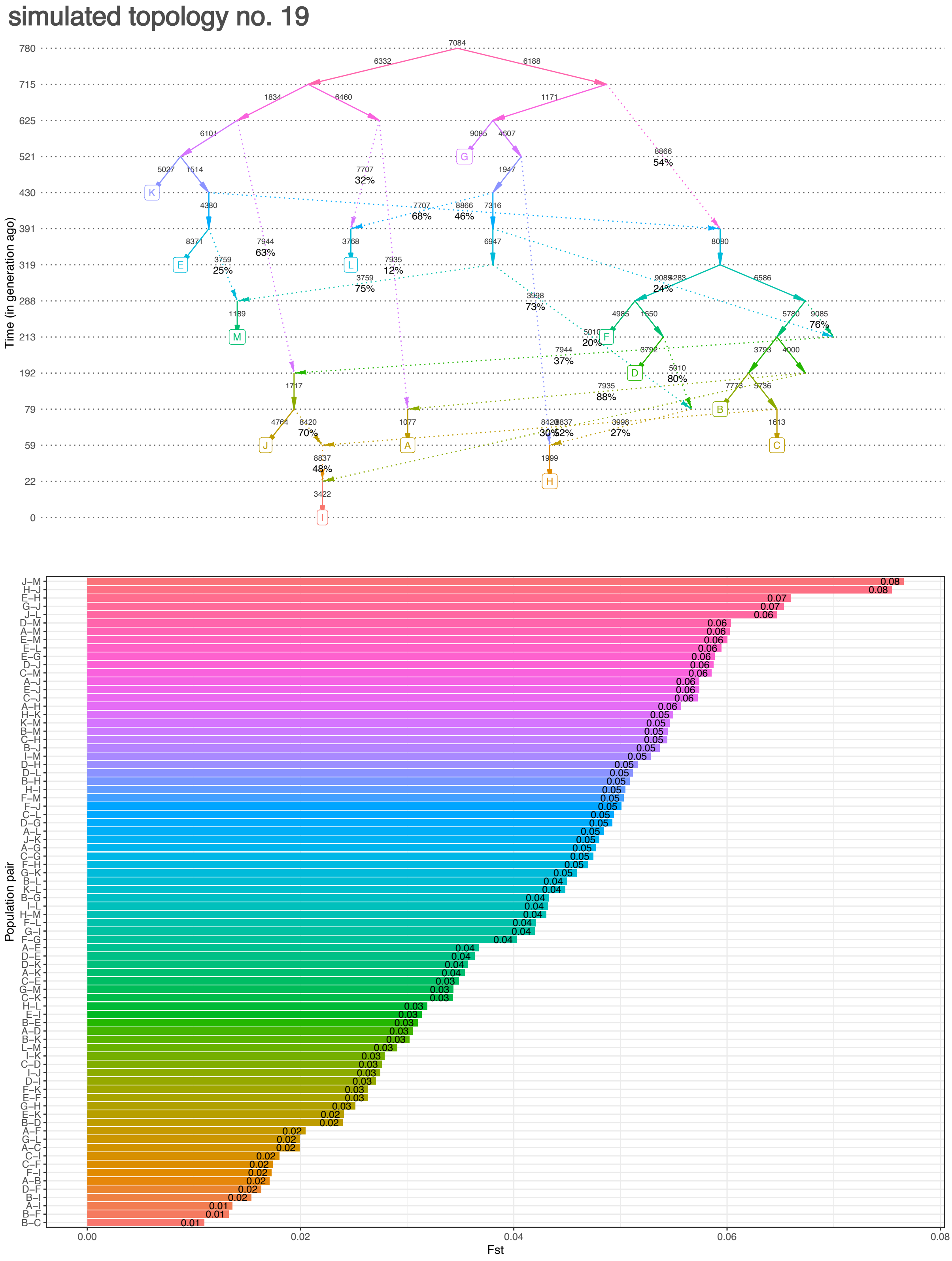
simulated topology no. 19
Time (in generation ago)
Population pair
Fst

simulated topology no. 19

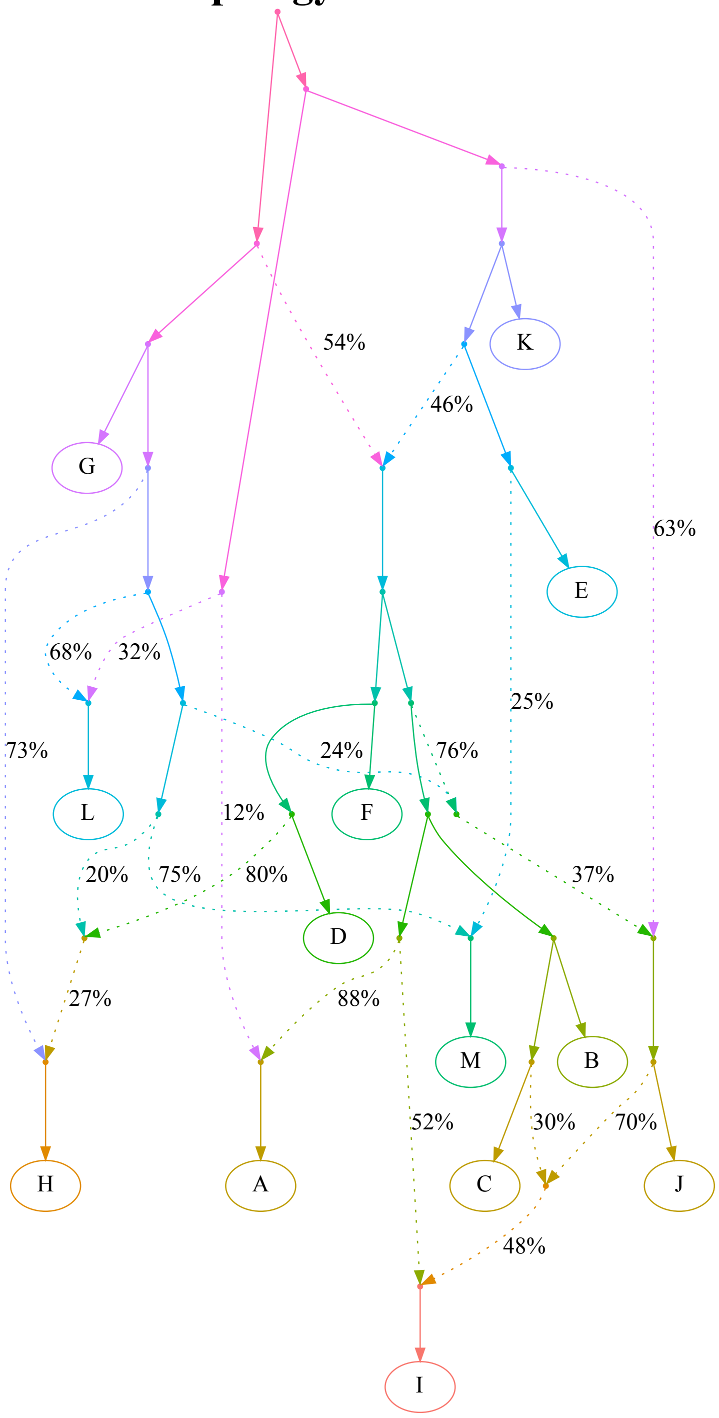

m

simulated topology no. 20

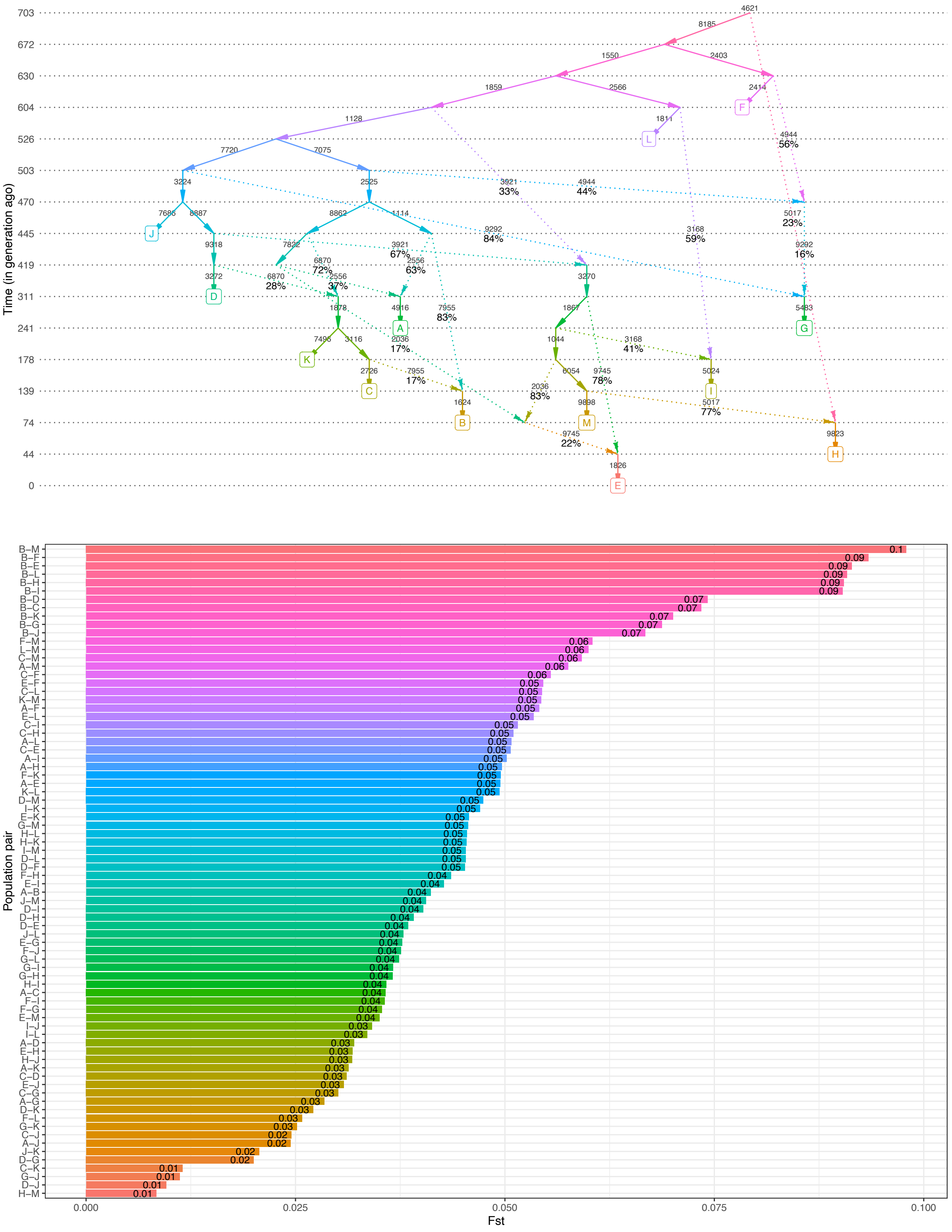

n

simulated topology no. 20

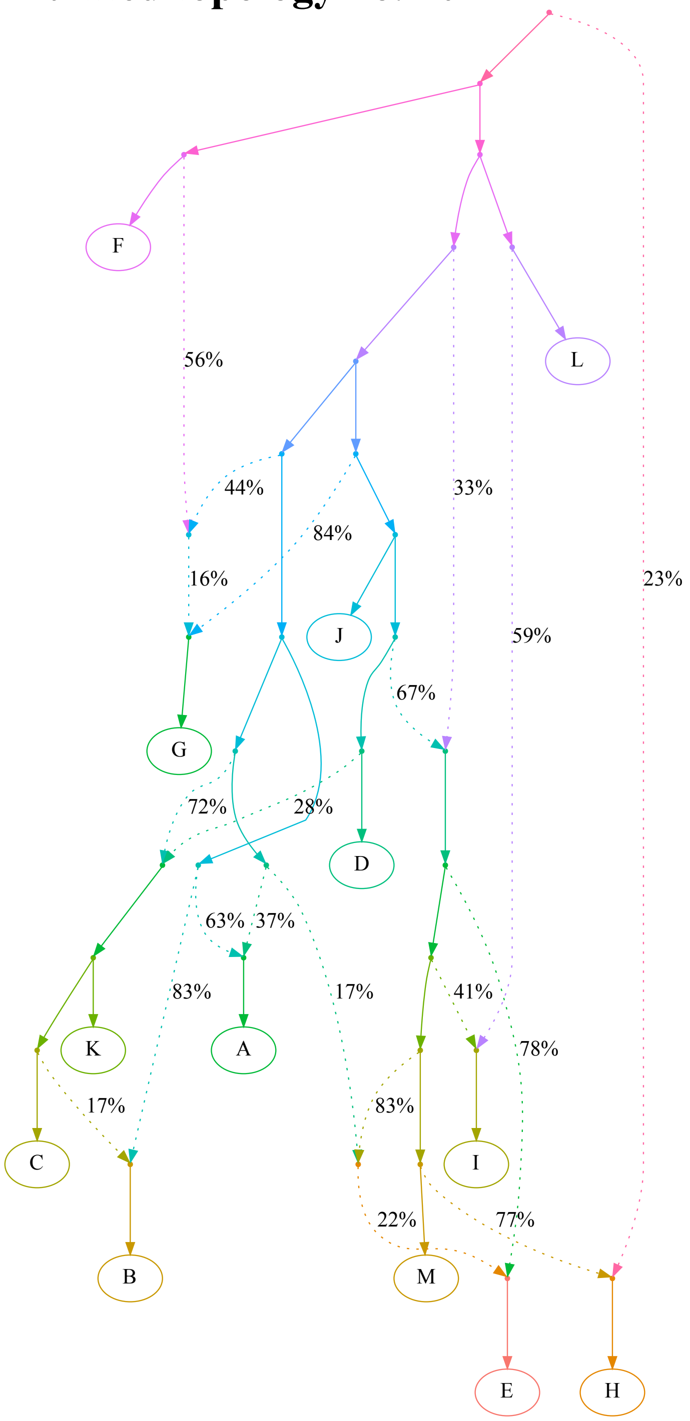

o

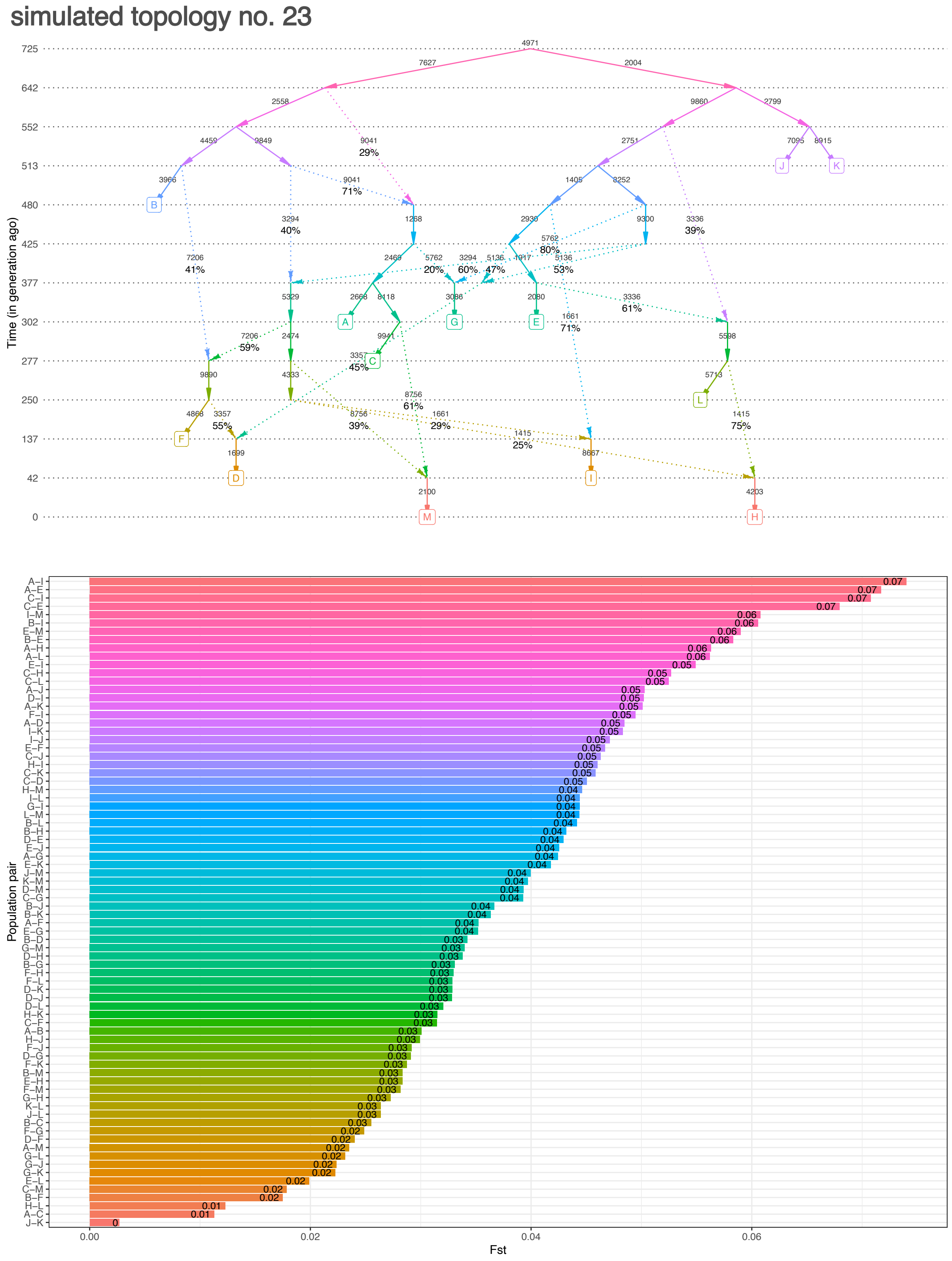

simulated topology no. 23
Time (in generation ago)
725
642
552
513
480
425
377
302
277
250
137
42
0
A
B
C
D
E
F
G
H
I
J
K
L
M
Population pair
Fst
0.00
0.02
0.04
0.06

p

simulated topology no. 23

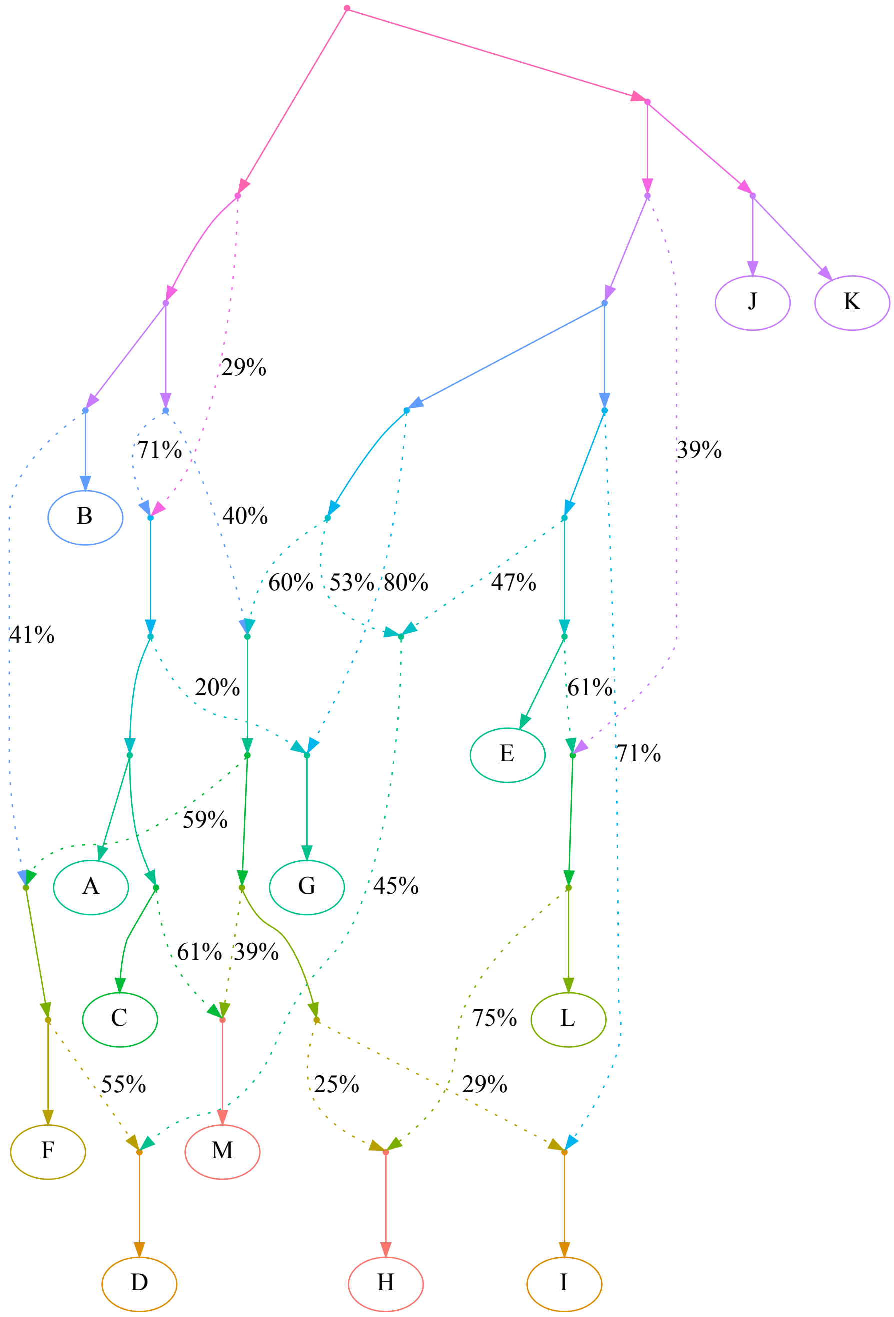

q

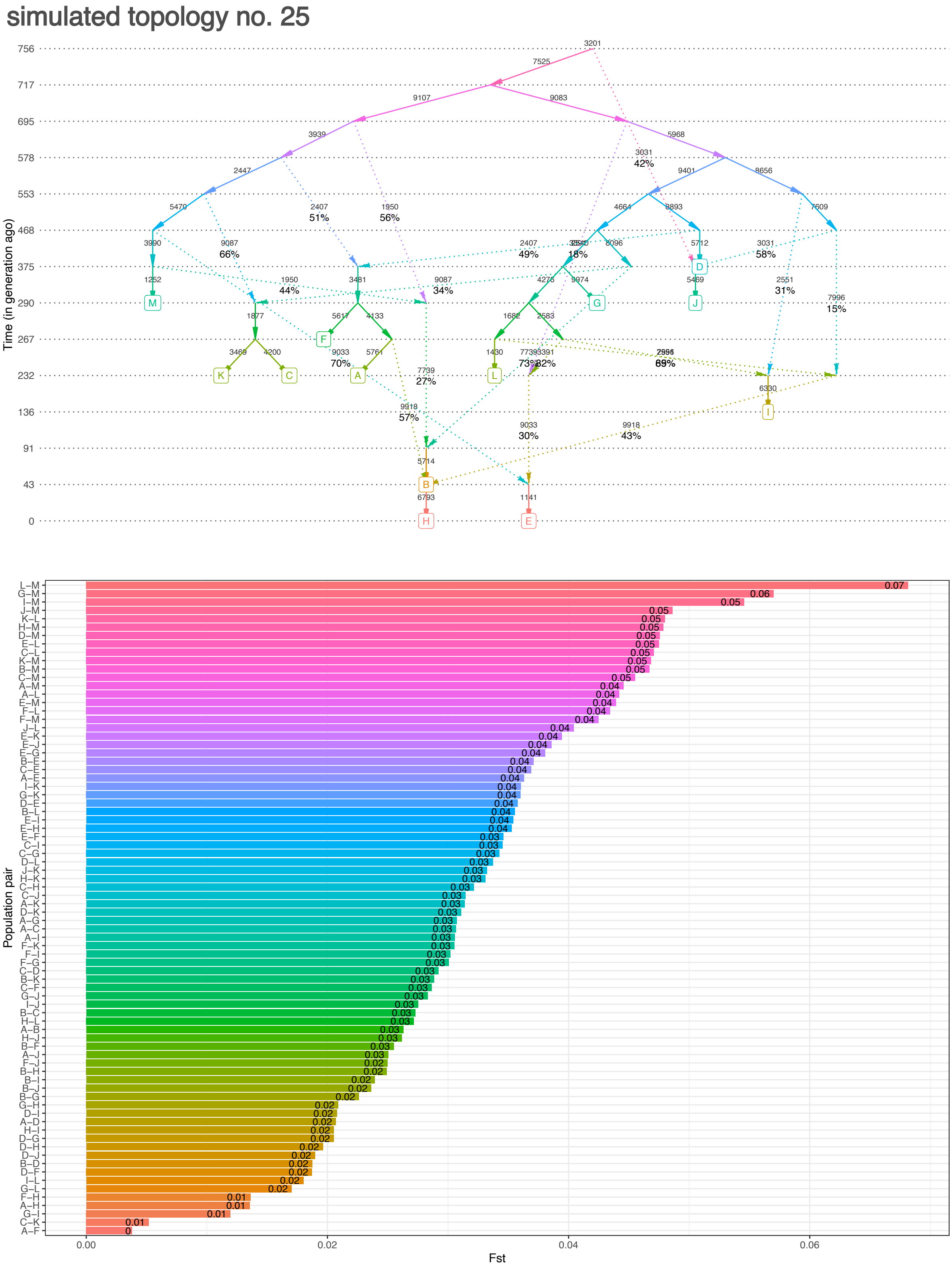
simulated topology no. 25
Time (in generation ago)
M
K
C
F
A
B
H
E
L
G
J
D
I
Population pair
Fst

r

simulated topology no. 25

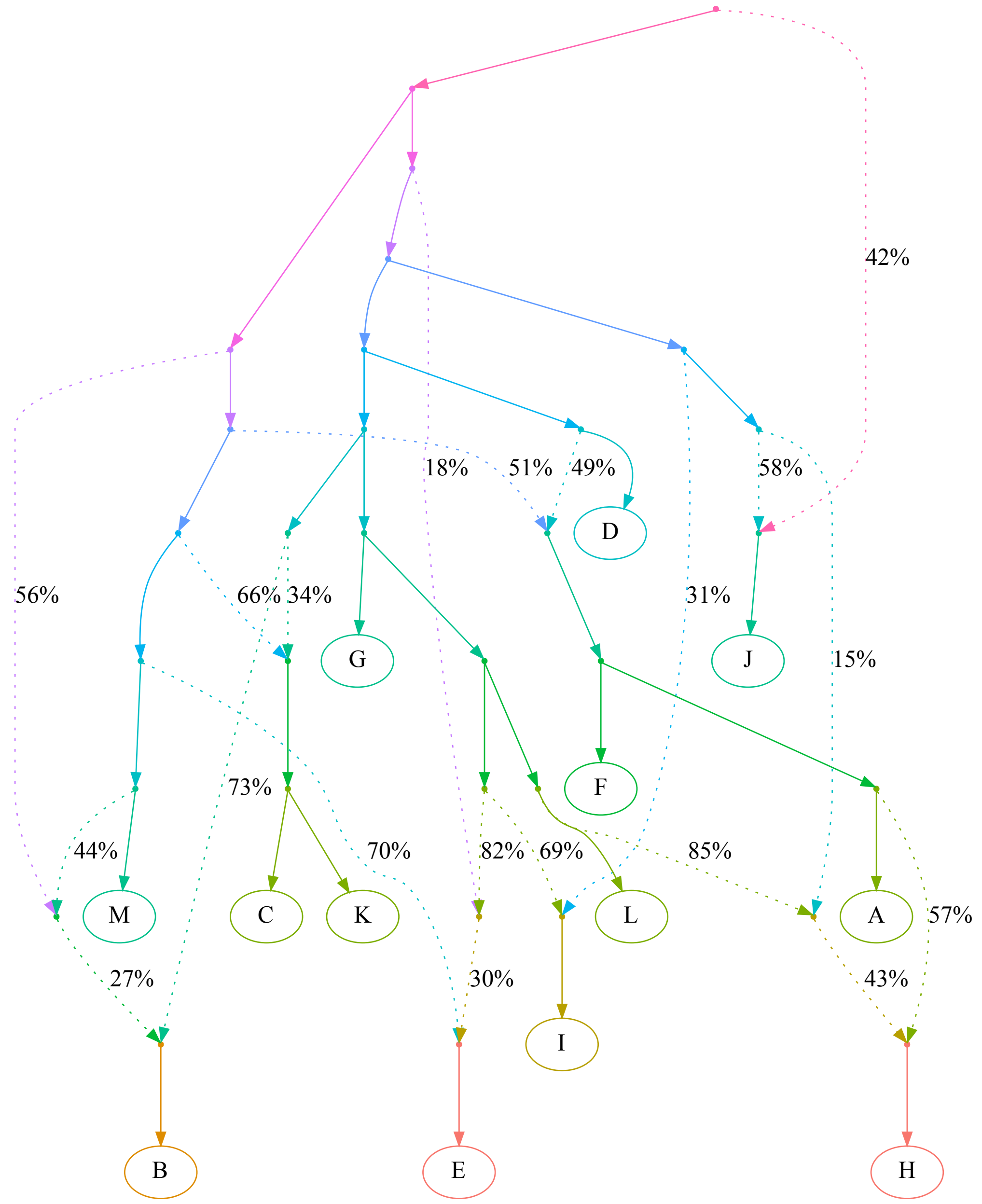

s

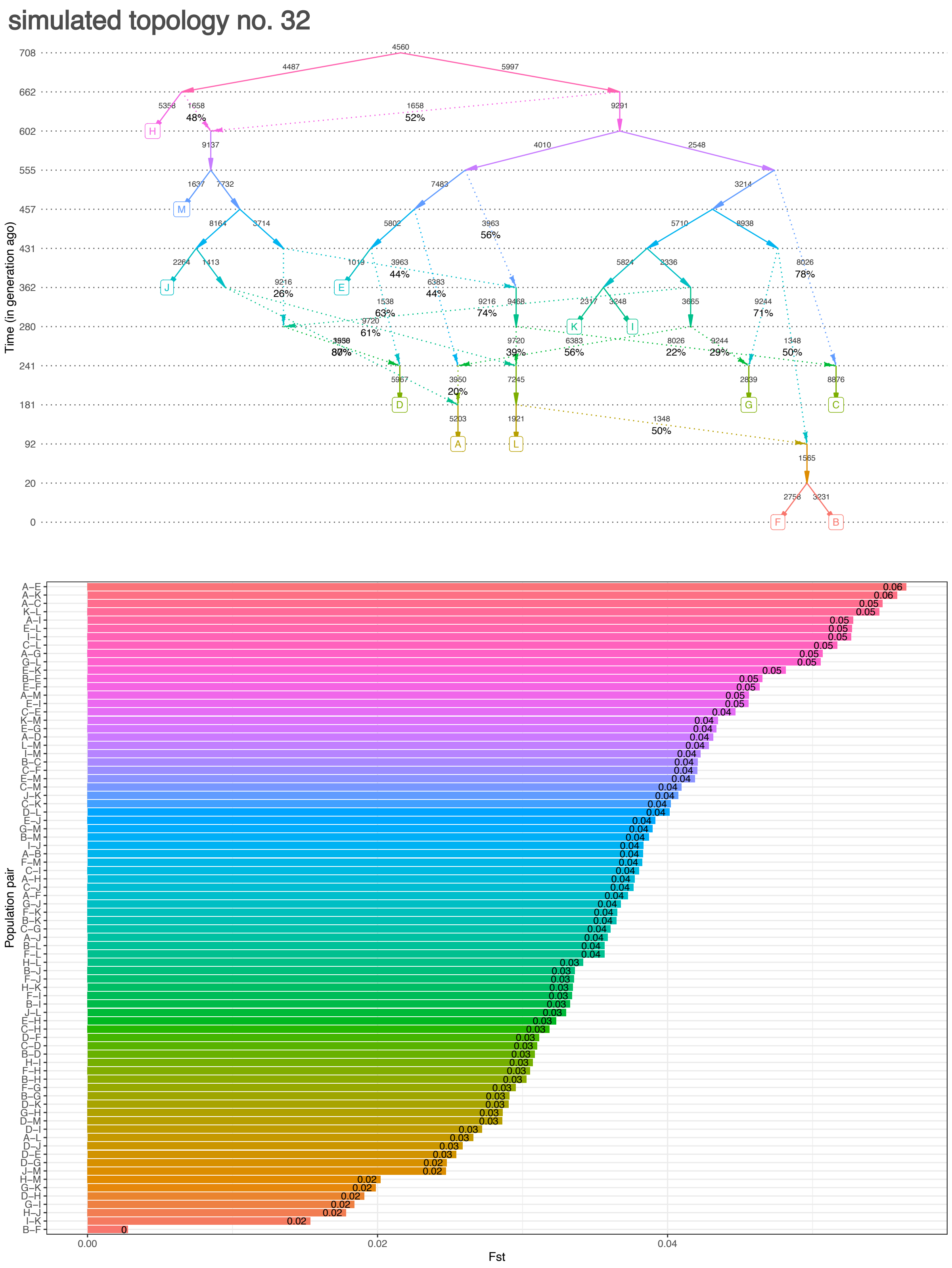

simulated topology no. 32
Time (in generation ago)
Population pair
Fst

t

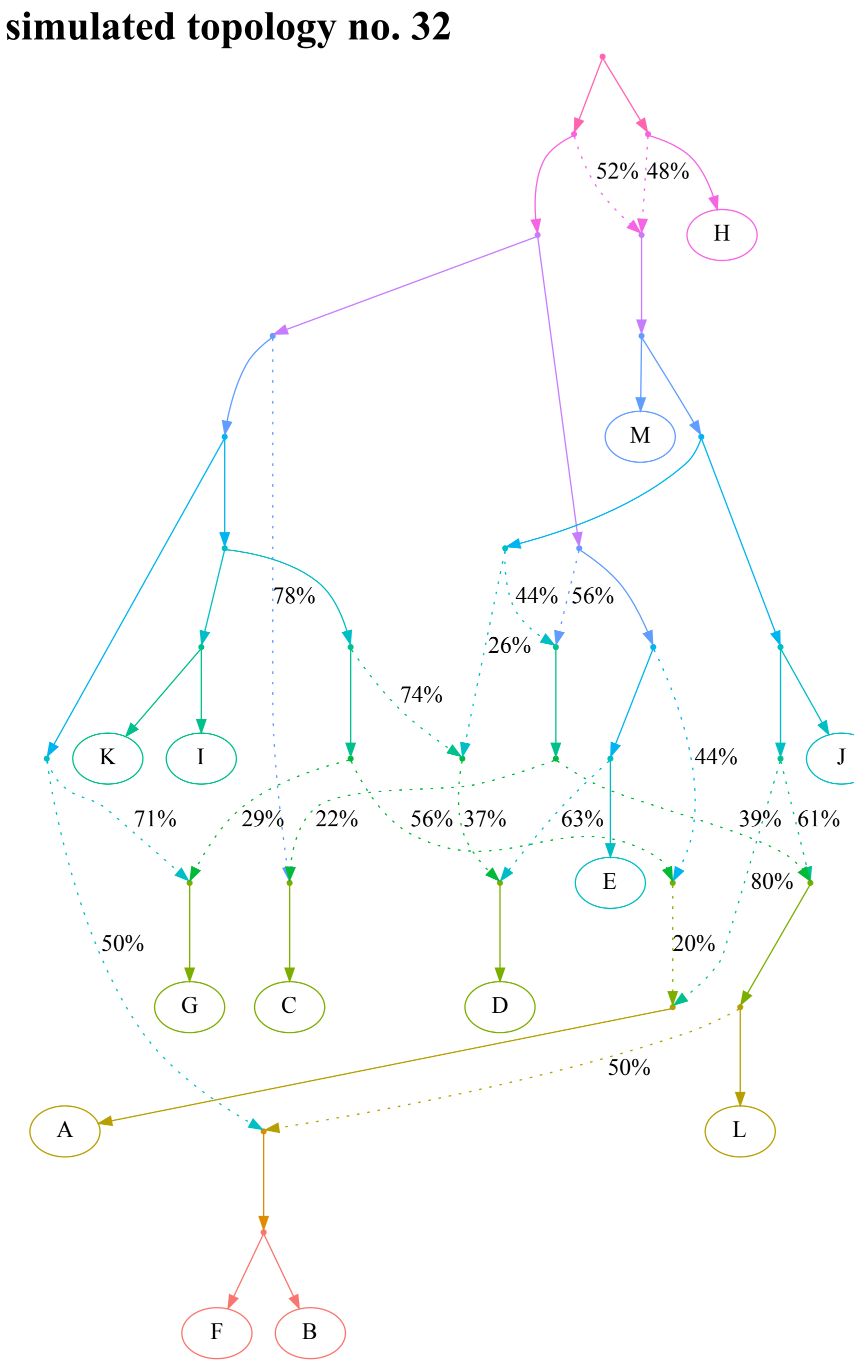

Simulated genetic histories in the shape of random admixture graphs including 13 populations and 10 pulse-like admixture events. Ten topologies are shown as examples, along with F_{ST} values for all population pairs: topologies no. 1 (**a, b**), 5, (**c, d**), 7 (**e, f**), 12 (**g, h**), 17 (**i, j**), 19 (**k, l**), 20 (**m, n**), 23 (**o, p**), 25 (**q, r**), and 32 (**s, t**). These topologies correspond to the case studies presented in **Fig. 4** and **Fig. S4**. In all cases, F_{ST} values are shown for simulation replicate no. 1, 300-Mbp-sized genomes, divergence times up to 800 generations, and diploid data. Time in generations is shown on the y-axis (for visual clarity, points on this axis are not spaced proportionately). Effective population sizes (in diploid individuals) are shown beside each

edge. Sampled populations are labelled by letters in squares. Gene flows going from the past to the future were simulated as unsampled ghost populations. The same simulated histories are shown in a form more accessible to topological analysis, but without any additional information, in panels **b**, **d**, **f**, **h**, **j, l, n, p, r, t**. The color gradients have no special meaning and are used for visual clarity only.

Figure S3.

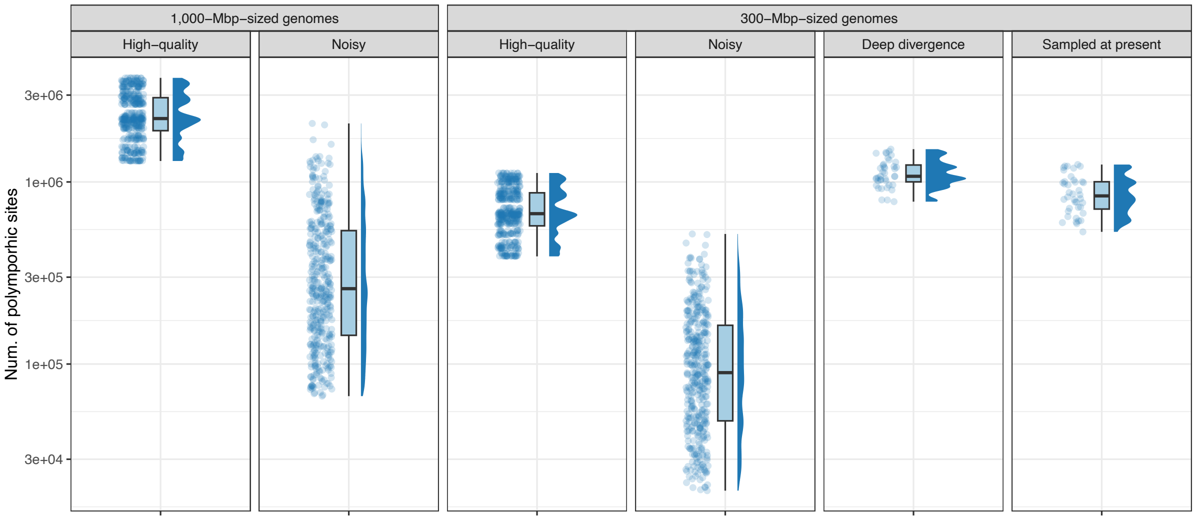

Number of SNPs with no missing data at the group level that are polymorphic in 13 simulated populations. SNP counts are summarized across simulated graph topologies and simulation replicates (for high-quality data) or subsampling replicates (for noisy pseudohaploid data). In the case of the "deep divergence" and "sampled at present" simulation setups, only one simulation replicate was generated.

Figure S4.

a

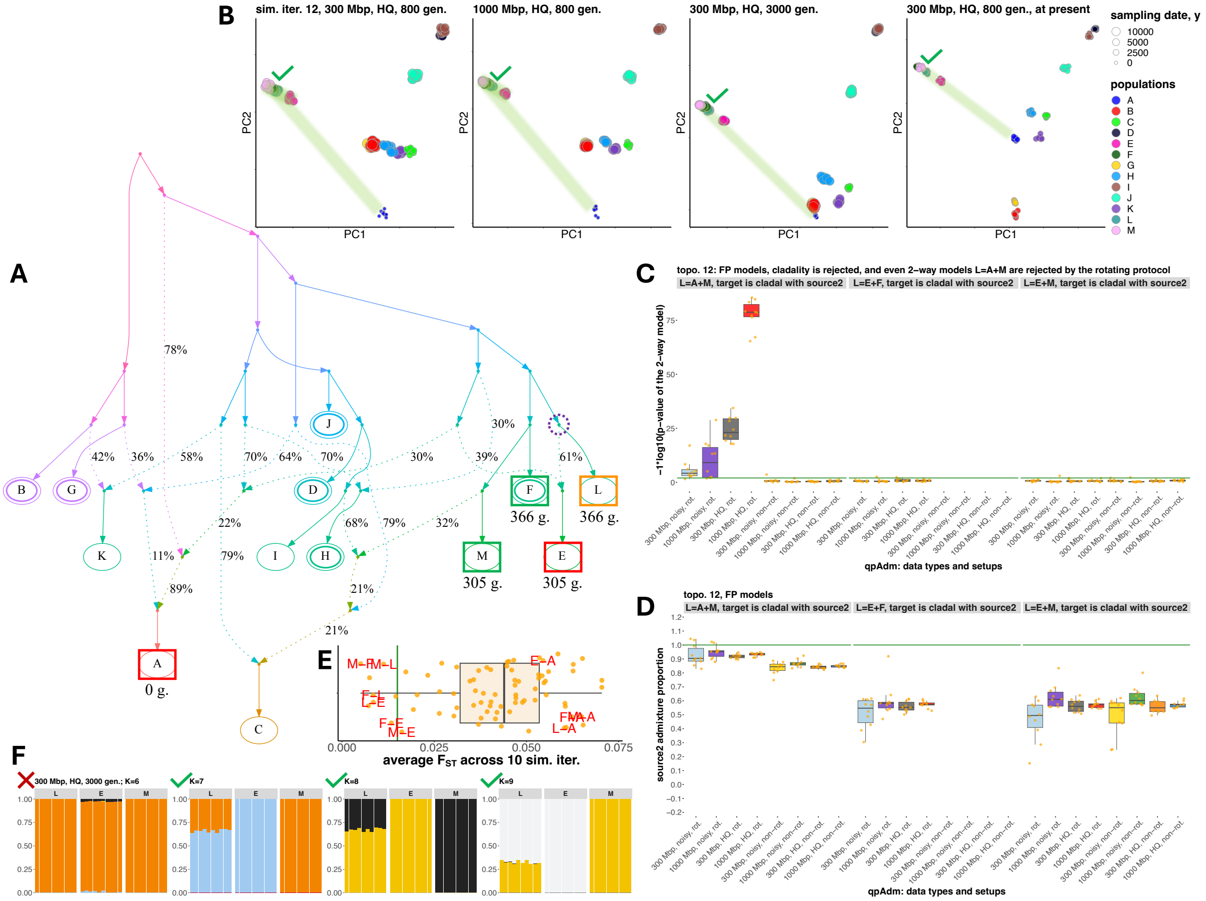

B
sim. iter. 12, 300 Mbp, HQ, 800 gen.
1000 Mbp, HQ, 800 gen.
300 Mbp, HQ, 3000 gen.
300 Mbp, HQ, 800 gen., at present
PC1
PC2
sampling date, y
10000
5000
2500
0
populations
A
B
C
D
E
F
G
H
I
J
K
L
M
A
78%
42%
36%
58%
70%
64%
70%
30%
30%
39%
61%
22%
68%
79%
32%
11%
79%
89%
21%
21%
J
D
F
L
366 g.
366 g.
M
E
305 g.
305 g.
K
I
H
A
0 g.
C
C
topo. 12: FP models, cladality is rejected, and even 2–way models L=A+M are rejected by the rotating protocol
L=A+M, target is cladal with source2
L=E+F, target is cladal with source2
L=E+M, target is cladal with source2
−1*log10(p–value of the 2–way model)
qpAdm: data types and setups
D
topo. 12, FP models
source2 admixture proportion
E
average F$_{ST}$ across 10 sim. iter.
0.000
0.025
0.050
0.075
F
300 Mbp, HQ, 3000 gen.; K=6
K=7
K=8
K=9

206 **b**

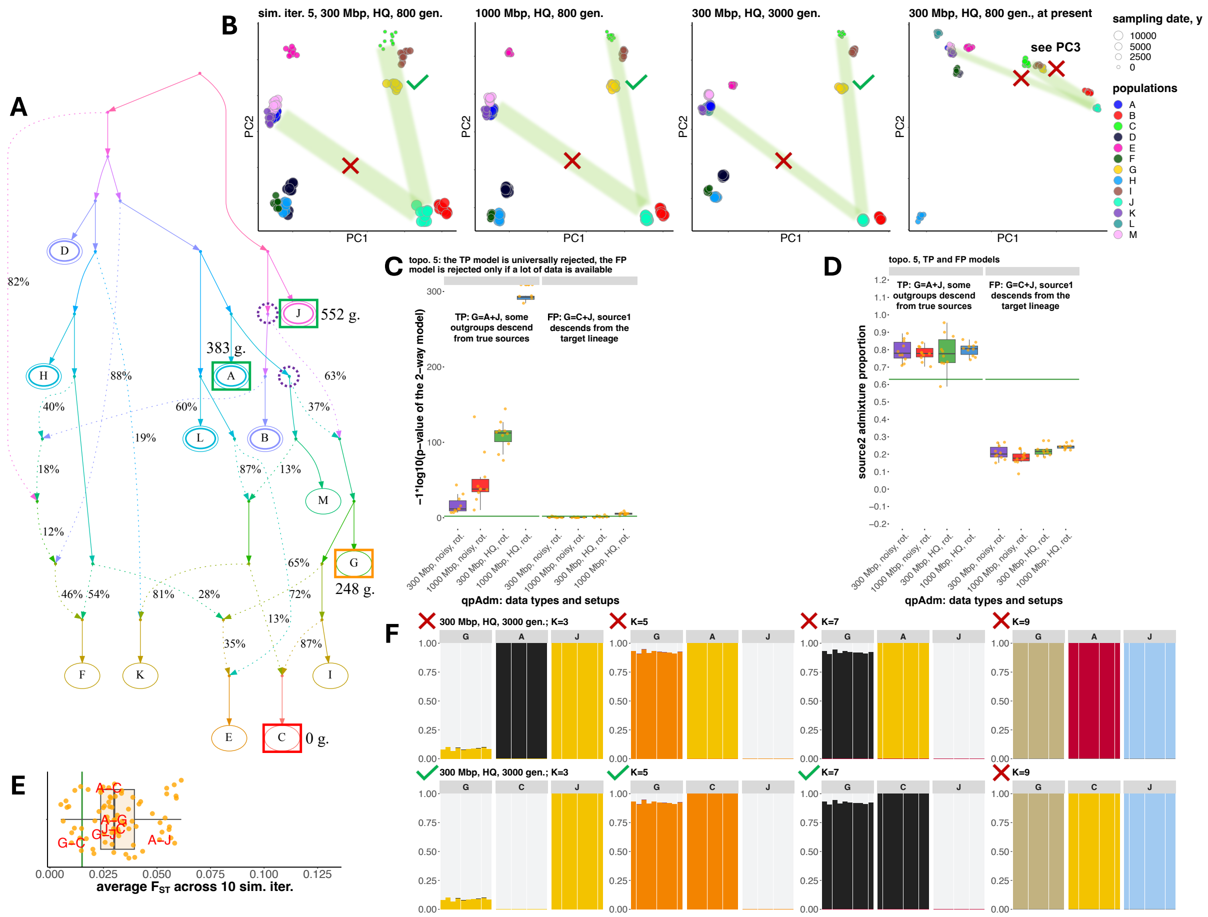
A
B
C
D
E
F
sim. iter. 5, 300 Mbp, HQ, 800 gen.
1000 Mbp, HQ, 800 gen.
300 Mbp, HQ, 3000 gen.
300 Mbp, HQ, 800 gen., at present
see PC3
sampling date, y
populations
topo. 5: the TP model is universally rejected, the FP model is rejected only if a lot of data is available
TP: G=A+J, some outgroups descend from true sources
FP: G=C+J, source1 descends from the target lineage
−1*log10(p−value of the 2−way model)
qpAdm: data types and setups
topo. 5, TP and FP models
source2 admixture proportion
average F_{ST} across 10 sim. iter.
300 Mbp, HQ, 3000 gen.; K=3
K=5
K=7
K=9
552 g.
383 g.
248 g.
0 g.

207
208

c

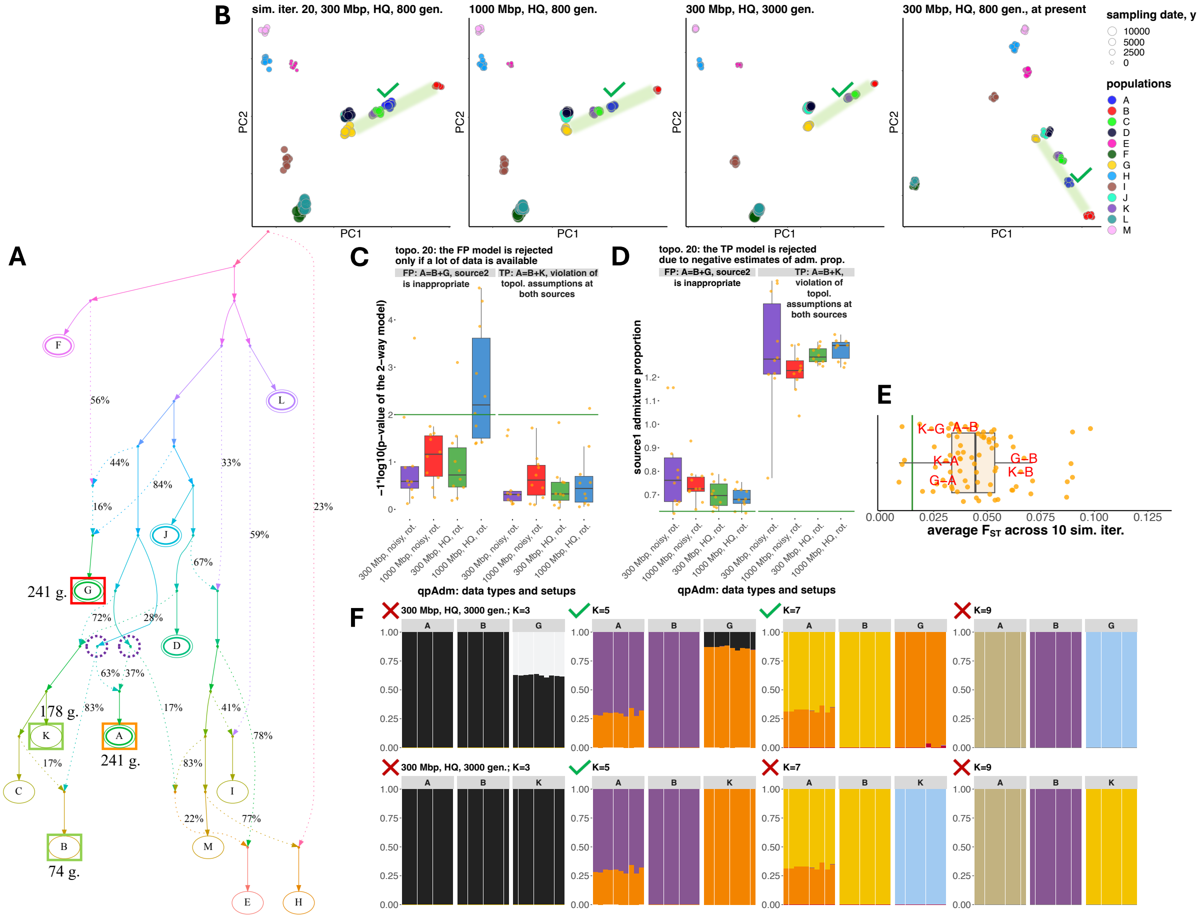

A
F
L
56%
44%
84%
33%
16%
J
23%
59%
67%
241 g.
G
72%
28%
D
63%
37%
178 g.
83%
17%
41%
K
A
78%
241 g.
17%
83%
C
I
B
22%
77%
M
74 g.
E
H
B
sim. iter. 20, 300 Mbp, HQ, 800 gen.
1000 Mbp, HQ, 800 gen.
300 Mbp, HQ, 3000 gen.
300 Mbp, HQ, 800 gen., at present
PC1
PC2
sampling date, y
10000
5000
2500
0
populations
A
B
C
D
E
F
G
H
I
J
K
L
M
C
topo. 20: the FP model is rejected only if a lot of data is available
FP: A=B+G, source2 is inappropriate
TP: A=B+K, violation of topol. assumptions at both sources
−1*log10(p−value of the 2−way model)
300 Mbp, noisy, rot.
1000 Mbp, noisy, rot.
300 Mbp, HQ, rot.
1000 Mbp, HQ, rot.
qpAdm: data types and setups
D
topo. 20: the TP model is rejected due to negative estimates of adm. prop.
FP: A=B+G, source2 is inappropriate
TP: A=B+K, violation of topol. assumptions at both sources
source1 admixture proportion
E
K=G
A=B
K=A
G=A
G=B
K=B
average F_{ST} across 10 sim. iter.
F
300 Mbp, HQ, 3000 gen.; K=3
K=5
K=7
K=9

d

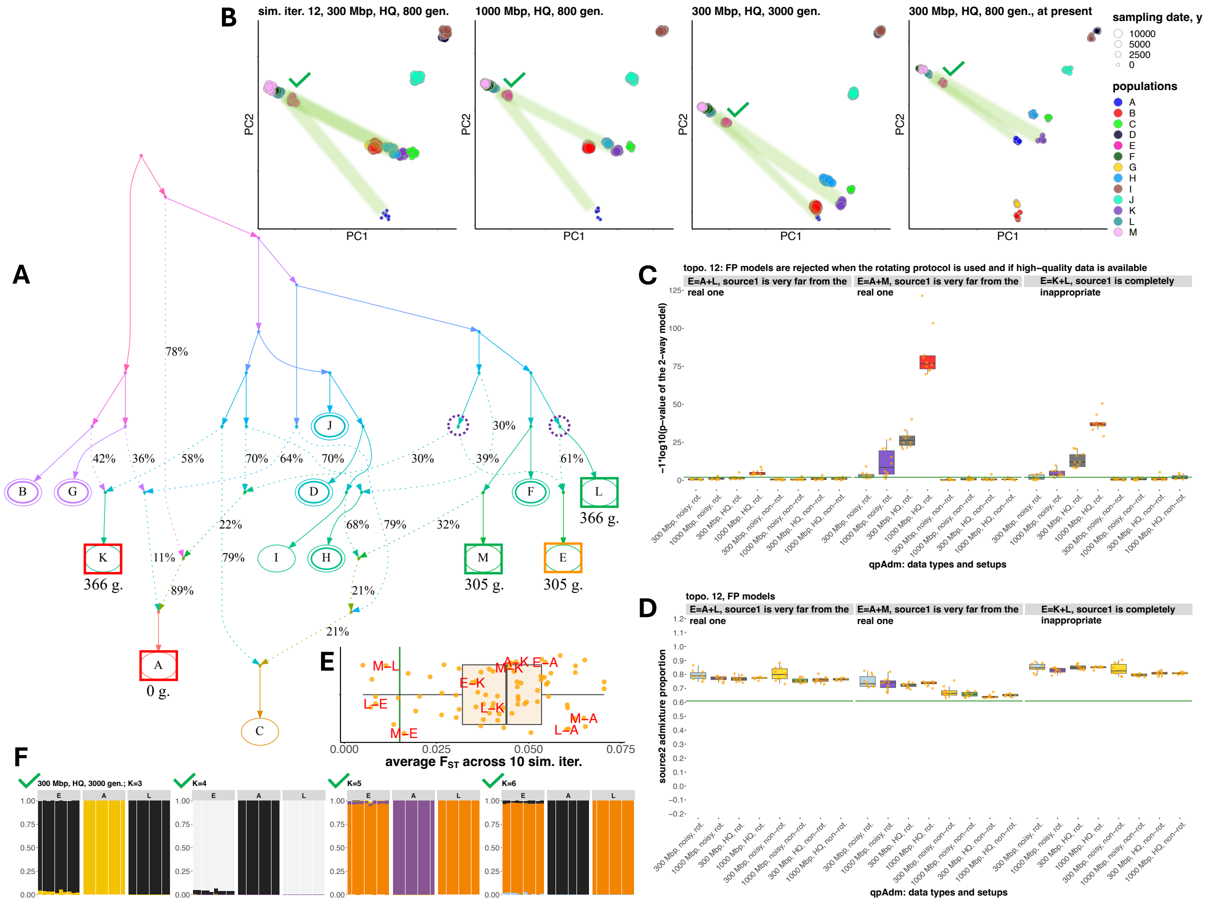

A
78%
42%
36%
58%
70%
64%
70%
30%
39%
30%
61%
22%
68%
79%
32%
11%
79%
89%
21%
21%
B
G
J
D
F
L
366 g.
K
366 g.
I
H
M
305 g.
E
305 g.
A
0 g.
C
B
sim. iter. 12, 300 Mbp, HQ, 800 gen.
1000 Mbp, HQ, 800 gen.
300 Mbp, HQ, 3000 gen.
300 Mbp, HQ, 800 gen., at present
PC1
PC2
sampling date, y
10000
5000
2500
0
populations
A
B
C
D
E
F
G
H
I
J
K
L
M
C
topo. 12: FP models are rejected when the rotating protocol is used and if high-quality data is available
E=A+L, source1 is very far from the real one
E=A+M, source1 is very far from the real one
E=K+L, source1 is completely inappropriate
−1*log10(p-value of the 2-way model)
qpAdm: data types and setups
300 Mbp, noisy, rot.
1000 Mbp, noisy, rot.
300 Mbp, HQ, rot.
1000 Mbp, HQ, rot.
300 Mbp, noisy, non-rot.
1000 Mbp, noisy, non-rot.
300 Mbp, HQ, non-rot.
1000 Mbp, HQ, non-rot.
D
topo. 12, FP models
E=A+L, source1 is very far from the real one
E=A+M, source1 is very far from the real one
E=K+L, source1 is completely inappropriate
source2 admixture proportion
qpAdm: data types and setups
E
M–L
L–E
M–E
E–K
L–K
M–K
A–K
E–A
M–A
L–A
0.000
0.025
0.050
0.075
average F_{ST} across 10 sim. iter.
F
300 Mbp, HQ, 3000 gen.; K=3
K=4
K=5
K=6

e

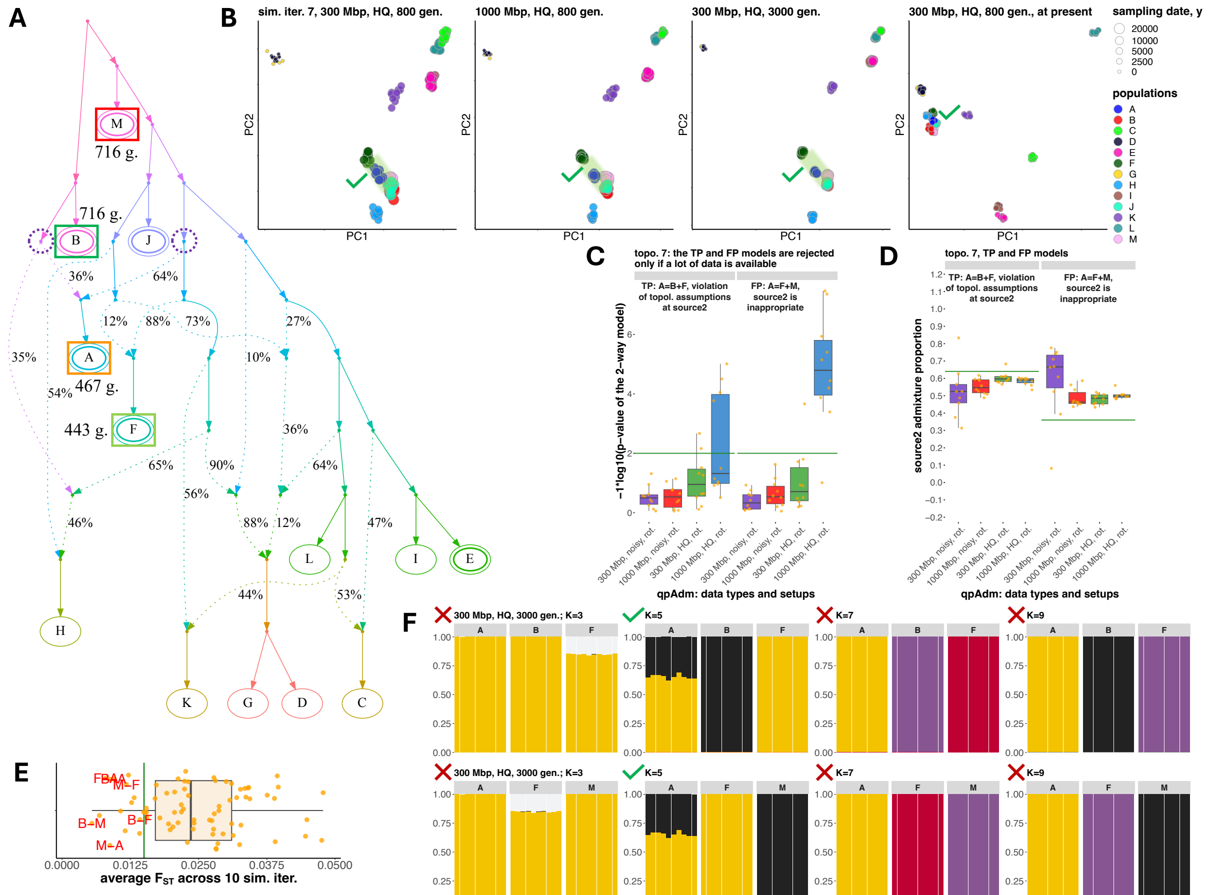
A
B
sim. iter. 7, 300 Mbp, HQ, 800 gen.
1000 Mbp, HQ, 800 gen.
300 Mbp, HQ, 3000 gen.
300 Mbp, HQ, 800 gen., at present
sampling date, y
populations
C
topo. 7: the TP and FP models are rejected only if a lot of data is available
TP: A=B+F, violation of topol. assumptions at source2
FP: A=F+M, source2 is inappropriate
−1*log10(p−value of the 2−way model)
qpAdm: data types and setups
D
topo. 7, TP and FP models
source2 admixture proportion
E
average F_ST across 10 sim. iter.
F
300 Mbp, HQ, 3000 gen.; K=3
K=5
K=7
K=9

f

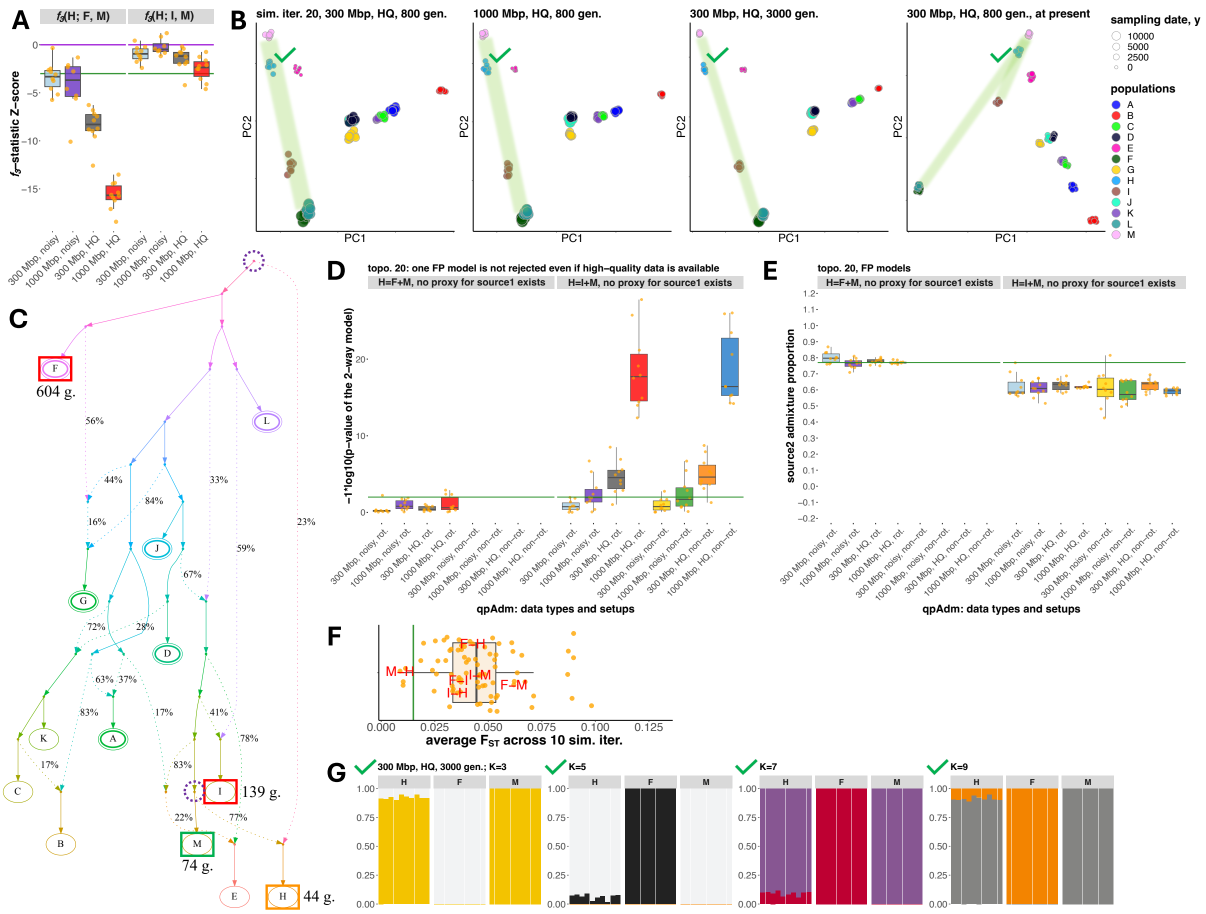
A
f_3(H; F, M)
f_3(H; I, M)
f_3-statistic Z-score
300 Mbp, noisy
1000 Mbp, noisy
300 Mbp, HQ
1000 Mbp, HQ
B
sim. iter. 20, 300 Mbp, HQ, 800 gen.
1000 Mbp, HQ, 800 gen.
300 Mbp, HQ, 3000 gen.
300 Mbp, HQ, 800 gen., at present
PC1
PC2
sampling date, y
10000
5000
2500
0
populations
A
B
C
D
E
F
G
H
I
J
K
L
M
C
604 g.
139 g.
74 g.
44 g.
56%
44%
16%
84%
33%
59%
23%
67%
72%
28%
63%
37%
83%
17%
41%
78%
17%
83%
22%
77%
D
topo. 20: one FP model is not rejected even if high-quality data is available
H=F+M, no proxy for source1 exists
H=I+M, no proxy for source1 exists
−1*log10(p-value of the 2-way model)
qpAdm: data types and setups
E
topo. 20, FP models
H=F+M, no proxy for source1 exists
H=I+M, no proxy for source1 exists
source2 admixture proportion
qpAdm: data types and setups
300 Mbp, noisy, rot.
1000 Mbp, noisy, rot.
300 Mbp, HQ, rot.
1000 Mbp, HQ, rot.
300 Mbp, noisy, non-rot.
1000 Mbp, noisy, non-rot.
300 Mbp, HQ, non-rot.
1000 Mbp, HQ, non-rot.
F
M–H
F–H
F–I
I–M
I–H
F–M
average F_{ST} across 10 sim. iter.
G
300 Mbp, HQ, 3000 gen.; K=3
K=5
K=7
K=9

g

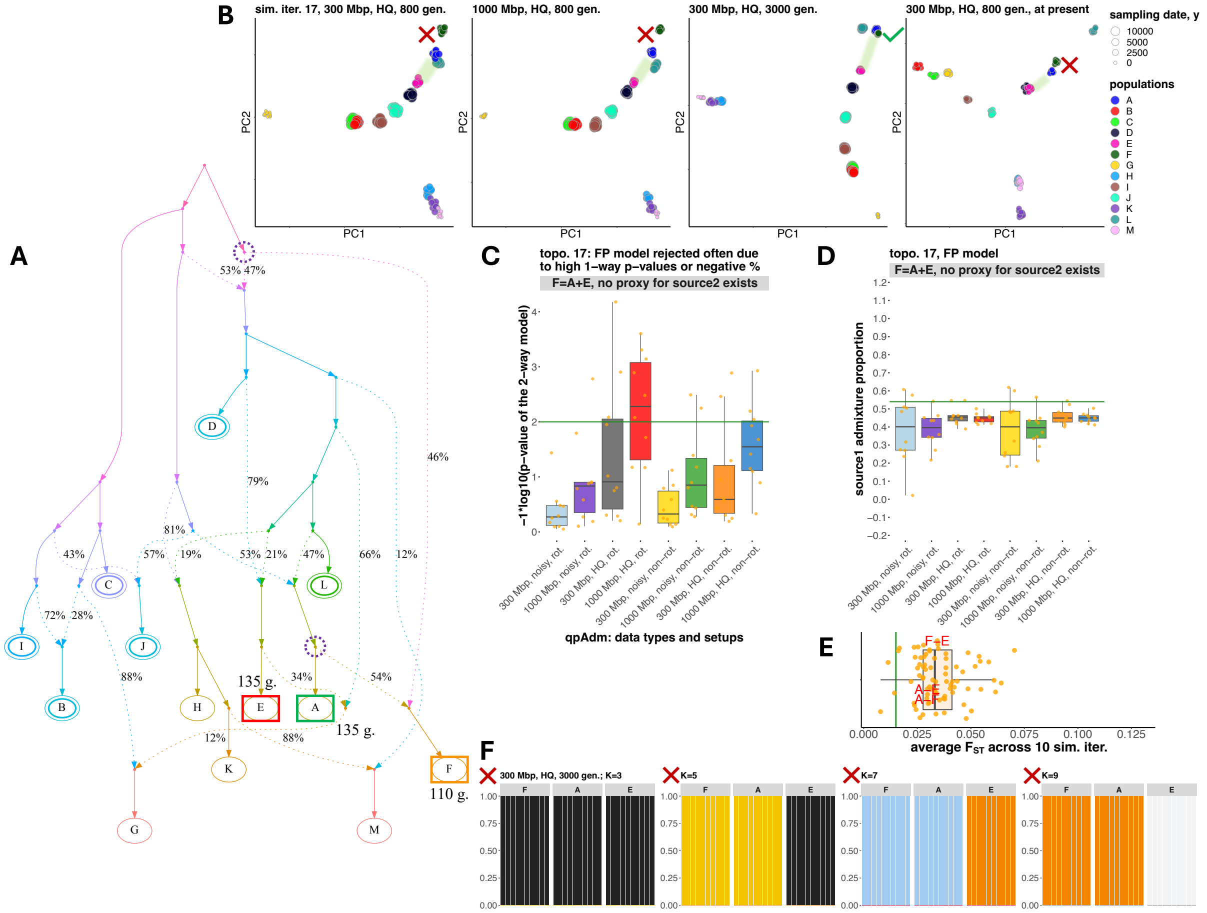
A
B
sim. iter. 17, 300 Mbp, HQ, 800 gen.
1000 Mbp, HQ, 800 gen.
300 Mbp, HQ, 3000 gen.
300 Mbp, HQ, 800 gen., at present
PC1
PC2
sampling date, y
10000
5000
2500
0
populations
A
B
C
D
E
F
G
H
I
J
K
L
M
C
topo. 17: FP model rejected often due to high 1-way p-values or negative %
F=A+E, no proxy for source2 exists
−1*log10(p−value of the 2−way model)
qpAdm: data types and setups
300 Mbp, noisy, rot.
1000 Mbp, noisy, rot.
300 Mbp, HQ, rot.
1000 Mbp, HQ, rot.
300 Mbp, noisy, non−rot.
1000 Mbp, noisy, non−rot.
300 Mbp, HQ, non−rot.
1000 Mbp, HQ, non−rot.
D
topo. 17, FP model
F=A+E, no proxy for source2 exists
source1 admixture proportion
E
F−E
A−E
A−F
average F_{ST} across 10 sim. iter.
F
300 Mbp, HQ, 3000 gen.; K=3
K=5
K=7
K=9
135 g.
110 g.
53% 47%
79%
46%
81%
43%
57%
19%
53%
21%
47%
66%
12%
72%
28%
88%
34%
54%
12%
88%

h

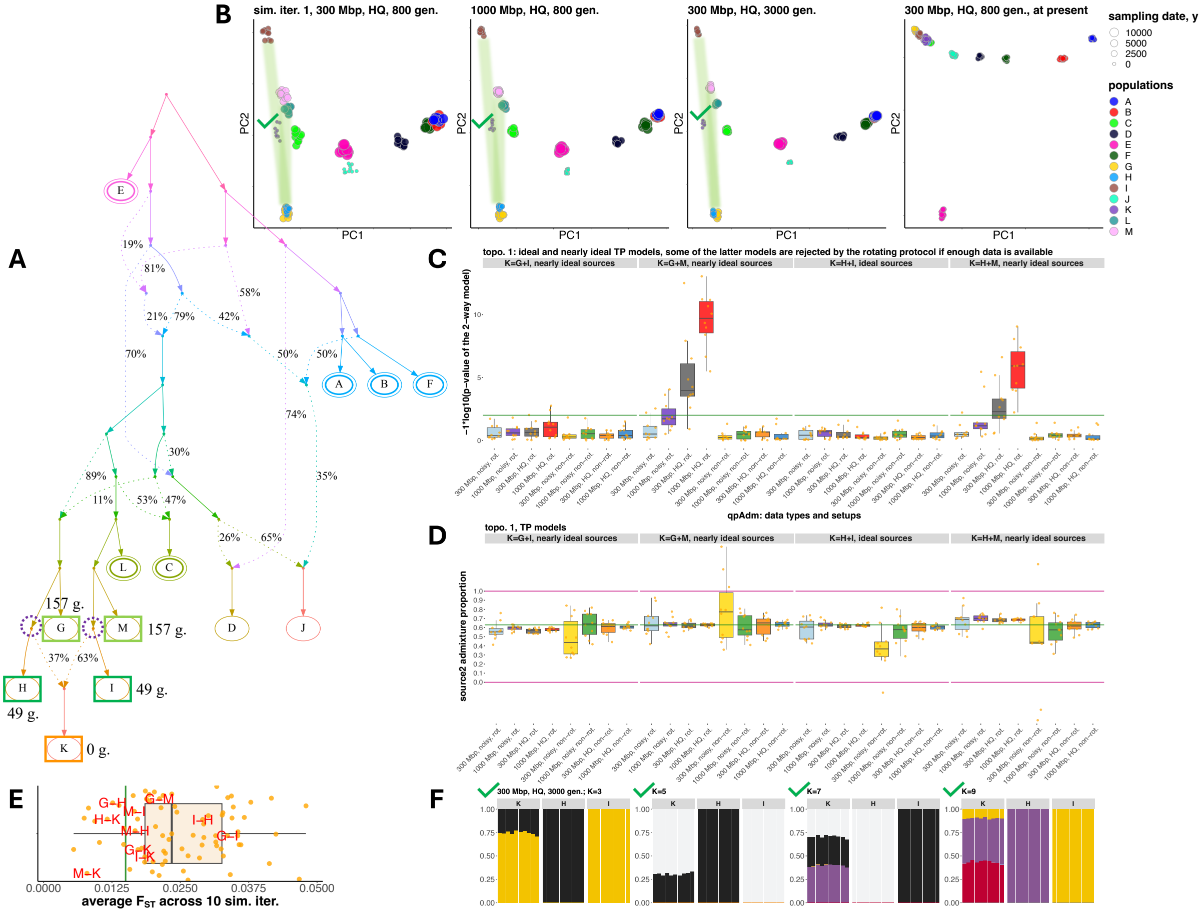
A
E
A
B
F
L
C
D
J
G
M
H
I
K
157 g.
157 g.
49 g.
49 g.
0 g.
19%
81%
21%
79%
58%
42%
70%
50%
50%
74%
30%
35%
89%
11%
53%
47%
26%
65%
37%
63%
B
sim. iter. 1, 300 Mbp, HQ, 800 gen.
1000 Mbp, HQ, 800 gen.
300 Mbp, HQ, 3000 gen.
300 Mbp, HQ, 800 gen., at present
PC1
PC2
sampling date, y
10000
5000
2500
0
populations
A
B
C
D
E
F
G
H
I
J
K
L
M
C
topo. 1: ideal and nearly ideal TP models, some of the latter models are rejected by the rotating protocol if enough data is available
K=G+I, nearly ideal sources
K=G+M, nearly ideal sources
K=H+I, ideal sources
K=H+M, nearly ideal sources
−1*log10(p−value of the 2−way model)
qpAdm: data types and setups
D
topo. 1, TP models
source2 admixture proportion
E
average FST across 10 sim. iter.
F
300 Mbp, HQ, 3000 gen.; K=3
K=5
K=7
K=9

i

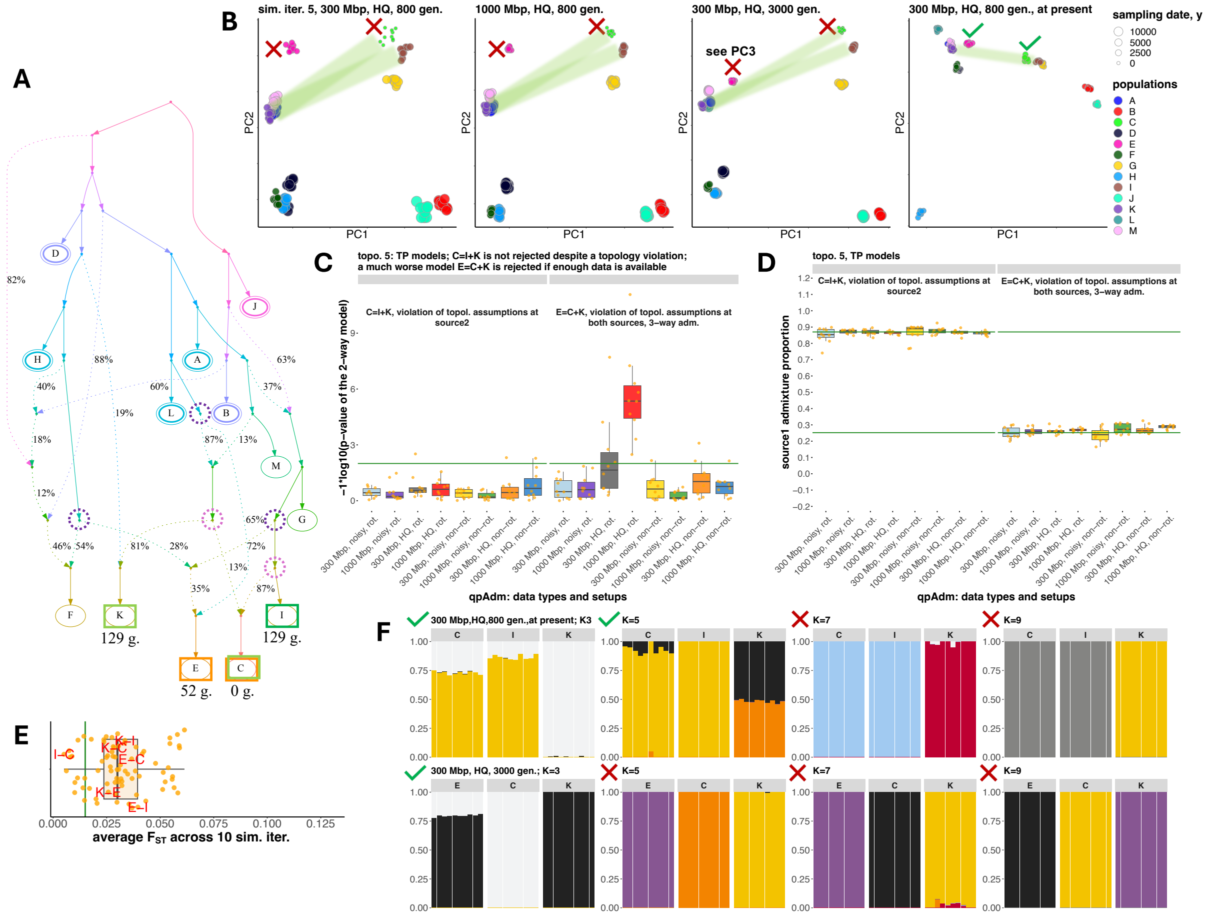

A
B
sim. iter. 5, 300 Mbp, HQ, 800 gen.
1000 Mbp, HQ, 800 gen.
300 Mbp, HQ, 3000 gen.
see PC3
300 Mbp, HQ, 800 gen., at present
PC1
PC2
sampling date, y
10000
5000
2500
0
populations
A
B
C
D
E
F
G
H
I
J
K
L
M
C
topo. 5: TP models; C=I+K is not rejected despite a topology violation;
a much worse model E=C+K is rejected if enough data is available
C=I+K, violation of topol. assumptions at source2
E=C+K, violation of topol. assumptions at both sources, 3-way adm.
−1*log10(p−value of the 2-way model)
qpAdm: data types and setups
D
topo. 5, TP models
C=I+K, violation of topol. assumptions at source2
E=C+K, violation of topol. assumptions at both sources, 3-way adm.
source1 admixture proportion
qpAdm: data types and setups
300 Mbp, noisy, rot.
1000 Mbp, noisy, rot.
300 Mbp, HQ, rot.
1000 Mbp, HQ, rot.
300 Mbp, noisy, non-rot.
1000 Mbp, noisy, non-rot.
300 Mbp, HQ, non-rot.
1000 Mbp, HQ, non-rot.
E
average F_ST across 10 sim. iter.
I−C
K−I
K−C
E−C
K−E
E−I
F
300 Mbp,HQ,800 gen.,at present; K3
K=5
K=7
K=9
300 Mbp, HQ, 3000 gen.; K=3
129 g.
52 g.
0 g.

j

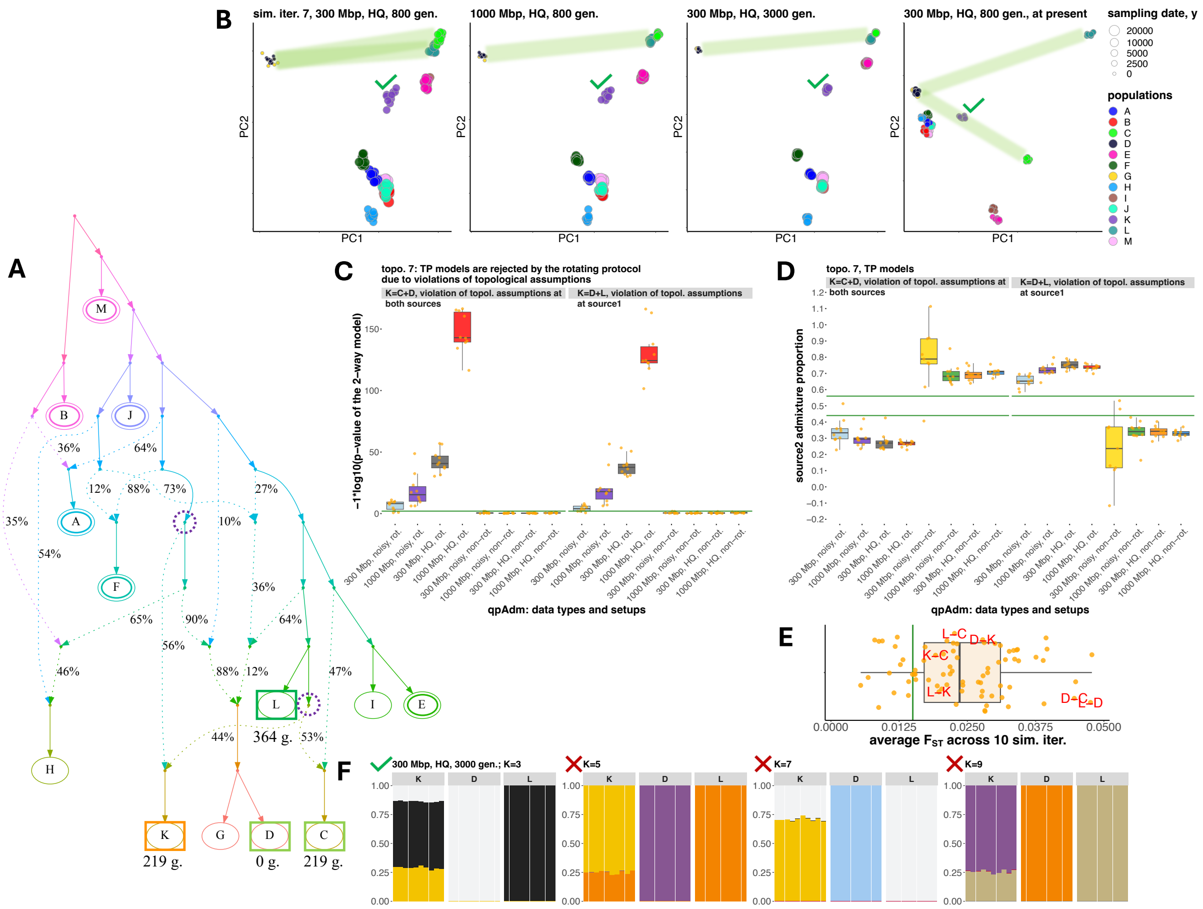
A
B
sim. iter. 7, 300 Mbp, HQ, 800 gen.
1000 Mbp, HQ, 800 gen.
300 Mbp, HQ, 3000 gen.
300 Mbp, HQ, 800 gen., at present
PC1
PC2
sampling date, y
20000
10000
5000
2500
0
populations
A
B
C
D
E
F
G
H
I
J
K
L
M
C
topo. 7: TP models are rejected by the rotating protocol due to violations of topological assumptions
K=C+D, violation of topol. assumptions at both sources
K=D+L, violation of topol. assumptions at source1
−1*log10(p−value of the 2-way model)
qpAdm: data types and setups
D
topo. 7, TP models
K=C+D, violation of topol. assumptions at both sources
K=D+L, violation of topol. assumptions at source1
source2 admixture proportion
qpAdm: data types and setups
E
average F_ST across 10 sim. iter.
F
300 Mbp, HQ, 3000 gen.; K=3
K=5
K=7
K=9
364 g.
219 g.
0 g.
219 g.

k

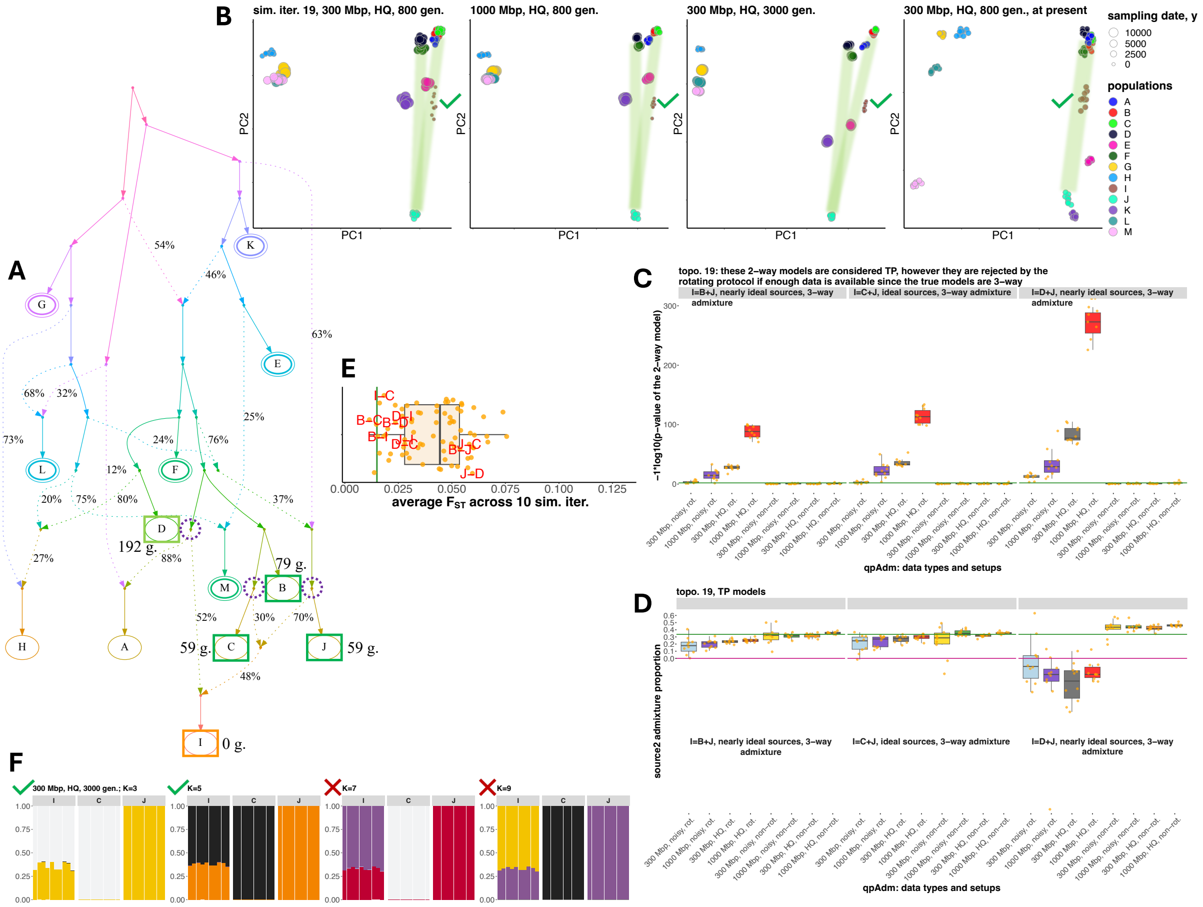
A
B
C
D
E
F
sim. iter. 19, 300 Mbp, HQ, 800 gen.
1000 Mbp, HQ, 800 gen.
300 Mbp, HQ, 3000 gen.
300 Mbp, HQ, 800 gen., at present
PC1
PC2
sampling date, y
10000
5000
2500
0
populations
A
B
C
D
E
F
G
H
I
J
K
L
M
topo. 19: these 2-way models are considered TP, however they are rejected by the rotating protocol if enough data is available since the true models are 3-way
I=B+J, nearly ideal sources, 3-way admixture
I=C+J, ideal sources, 3-way admixture
I=D+J, nearly ideal sources, 3-way admixture
−1*log10(p-value of the 2-way model)
qpAdm: data types and setups
topo. 19, TP models
source2 admixture proportion
average F$_{ST}$ across 10 sim. iter.
54%
46%
63%
68%
32%
73%
25%
24%
76%
12%
20%
75%
80%
37%
27%
88%
52%
30%
70%
48%
192 g.
79 g.
59 g.
0 g.
300 Mbp, HQ, 3000 gen.; K=3
K=5
K=7
K=9

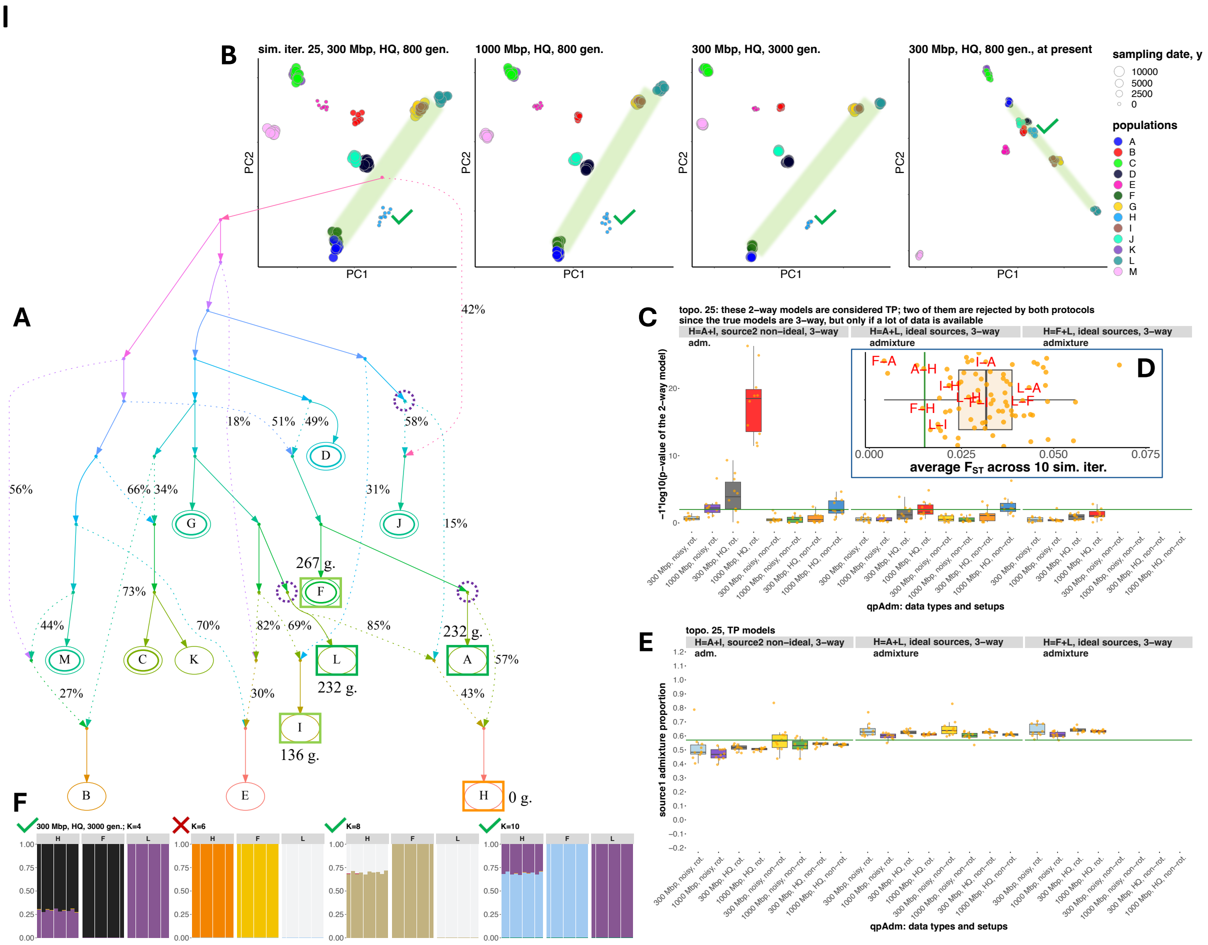

B
sim. iter. 25, 300 Mbp, HQ, 800 gen.
1000 Mbp, HQ, 800 gen.
300 Mbp, HQ, 3000 gen.
300 Mbp, HQ, 800 gen., at present
PC1
PC2
sampling date, y
10000
5000
2500
0
populations
A
B
C
D
E
F
G
H
I
J
K
L
M
A
42%
18%
51%
49%
58%
56%
66%
34%
31%
15%
267 g.
73%
44%
70%
82%
69%
85%
232 g.
57%
27%
30%
43%
232 g.
136 g.
0 g.
C
topo. 25: these 2–way models are considered TP; two of them are rejected by both protocols
since the true models are 3–way, but only if a lot of data is available
H=A+I, source2 non–ideal, 3–way adm.
H=A+L, ideal sources, 3–way admixture
H=F+L, ideal sources, 3–way admixture
−1*log10(p–value of the 2–way model)
qpAdm: data types and setups
D
F→A
A→H
I→A
I→H
L→A
F→H
L→F
L→I
average F_{ST} across 10 sim. iter.
E
topo. 25, TP models
H=A+I, source2 non–ideal, 3–way adm.
H=A+L, ideal sources, 3–way admixture
H=F+L, ideal sources, 3–way admixture
source1 admixture proportion
qpAdm: data types and setups
300 Mbp, noisy, rot.
1000 Mbp, noisy, rot.
300 Mbp, HQ, rot.
1000 Mbp, HQ, rot.
300 Mbp, noisy, non–rot.
1000 Mbp, noisy, non–rot.
300 Mbp, HQ, non–rot.
1000 Mbp, HQ, non–rot.
F
300 Mbp, HQ, 3000 gen.; K=4
K=6
K=8
K=10

m

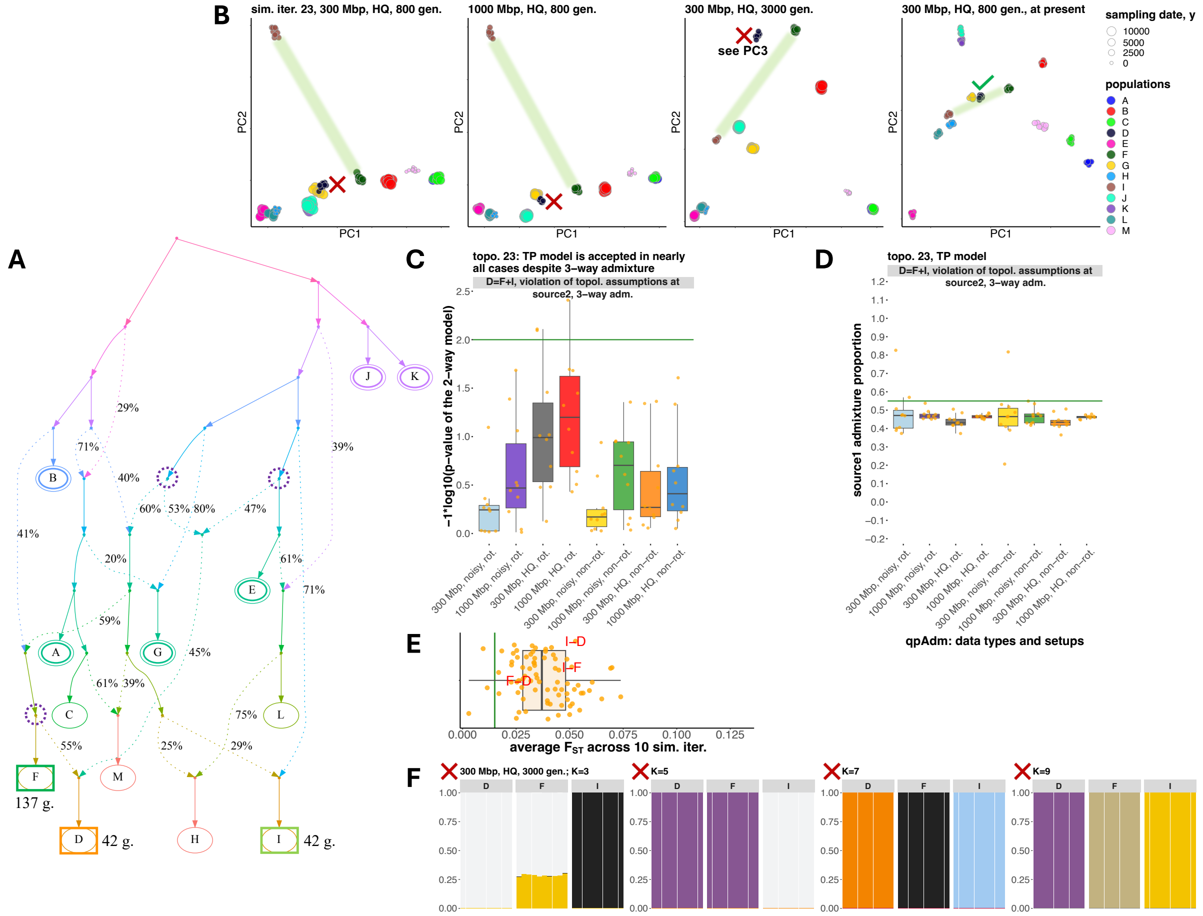
A
B
sim. iter. 23, 300 Mbp, HQ, 800 gen.
1000 Mbp, HQ, 800 gen.
300 Mbp, HQ, 3000 gen.
see PC3
300 Mbp, HQ, 800 gen., at present
PC1
PC2
sampling date, y
10000
5000
2500
0
populations
A
B
C
D
E
F
G
H
I
J
K
L
M
137 g.
42 g.
42 g.
C
topo. 23: TP model is accepted in nearly all cases despite 3–way admixture
D=F+I, violation of topol. assumptions at source2, 3–way adm.
−1*log10(p−value of the 2−way model)
300 Mbp, noisy, rot.
1000 Mbp, noisy, rot.
300 Mbp, HQ, rot.
1000 Mbp, HQ, rot.
300 Mbp, noisy, non−rot.
1000 Mbp, noisy, non−rot.
300 Mbp, HQ, non−rot.
1000 Mbp, HQ, non−rot.
D
topo. 23, TP model
D=F+I, violation of topol. assumptions at source2, 3–way adm.
source1 admixture proportion
qpAdm: data types and setups
E
average F_{ST} across 10 sim. iter.
I–D
I–F
F–D
F
300 Mbp, HQ, 3000 gen.; K=3
K=5
K=7
K=9

Case studies illustrating false (**a-g**) and true (**b, c, e, h-m**) positive *qpAdm* models of various topological types, with corresponding simulated histories, PCA, F_{ST}, and unsupervised *ADMIXTURE* results also shown. Each panel of this figure illustrates one case study and is composed of several standardized sub-panels described below. **A**) Simulated history in the form of an admixture graph (only topology is shown here, for divergence/admixture dates and effective population sizes see the respective simulated history presented in **Fig. S2**). Sampled populations are marked by letters. The target population from the *qpAdm* models illustrated here is enclosed in an orange rectangle; correct proxy sources – in green rectangles, correct proxy sources with violations of the topological assumptions of *qpAdm* – in green-yellow rectangles, and inappropriate proxy sources, if any – in red rectangles. Sampling dates for these groups (in generations before present) are shown beside the rectangles (the dates are from the "shallow" simulations up to 800 generations deep). True simulated ancestry source(s) for the target are enclosed in dashed circles. Six populations used as outgroups for the non-rotating *qpAdm* protocol are enclosed in double ovals. **B**) Two-dimensional PCA plots for four simulated high-quality datasets indicated in plot titles. Simulated populations are coloured according to the legend on the right, and larger points correspond to older sampling dates. The space between the proxy sources is shaded in green. If admixture model(s) are supported by three-dimensional (sic!) PCA (PC1 vs. PC2 vs. PC3) according to the criteria listed in Methods, a green tick mark is placed beside the target group in the plot, and a red cross mark is placed otherwise. **C**) Boxplots summarizing *p*-values of two-way *qpAdm* models (indicated in plot titles) across simulation or subsampling replicates, grouped by simulation setups and *qpAdm* protocols as indicated on the x-axis. Green lines show the *p*-value threshold of 0.01 used in this study for model rejection. *P*-values for one-way models are not shown for brevity. Some *qpAdm* models were not tested using the non-rotating protocol since at least one "left" population belongs to the fixed set of six "right" populations. **D**) Boxplots summarizing estimated admixture proportions across simulation or subsampling replicates, grouped by simulation setups and *qpAdm* protocols. The proportions are shown either for the first or second proxy source, as indicated on the y-axis; green lines show the simulated admixture proportion. **E**) All F_{ST} values for population pairs from the simulated history. Average F_{ST} values are shown across 10 simulation replicates for 1000-Mbp-sized genomes and high-quality data. Population pairs formed by components of the illustrated two-way *qpAdm* model(s) are labelled in red. The green line shows median F_{ST} (0.015) for the Bronze Age West Asian populations analyzed by Lazaridis et al. (2016) as an example of human genetic diversity at the sub-continental scale. **F**) Ancestry proportions estimated with unsupervised *ADMIXTURE* for the groups constituting the *qpAdm* model(s). For brevity, results are shown for one or two *qpAdm* models, for one simulated dataset indicated in the plot title (usually simulation setup no. 3: 300-Mbp-sized genomes, max. divergence date of 3,000 generations, high-quality data), and for four selected *K* values. If admixture model(s) are supported by this analysis at a given *K* value, a green tick mark is placed beside the target group in the plot, and a red cross mark is placed otherwise. In panel **f**, f_3-statistics f_3(target; proxy source$_1$, proxy source$_2$) are shown in sub-panel **A**, and the other information is shown in sub-panels **B** to **G**.

Figure S5.

a

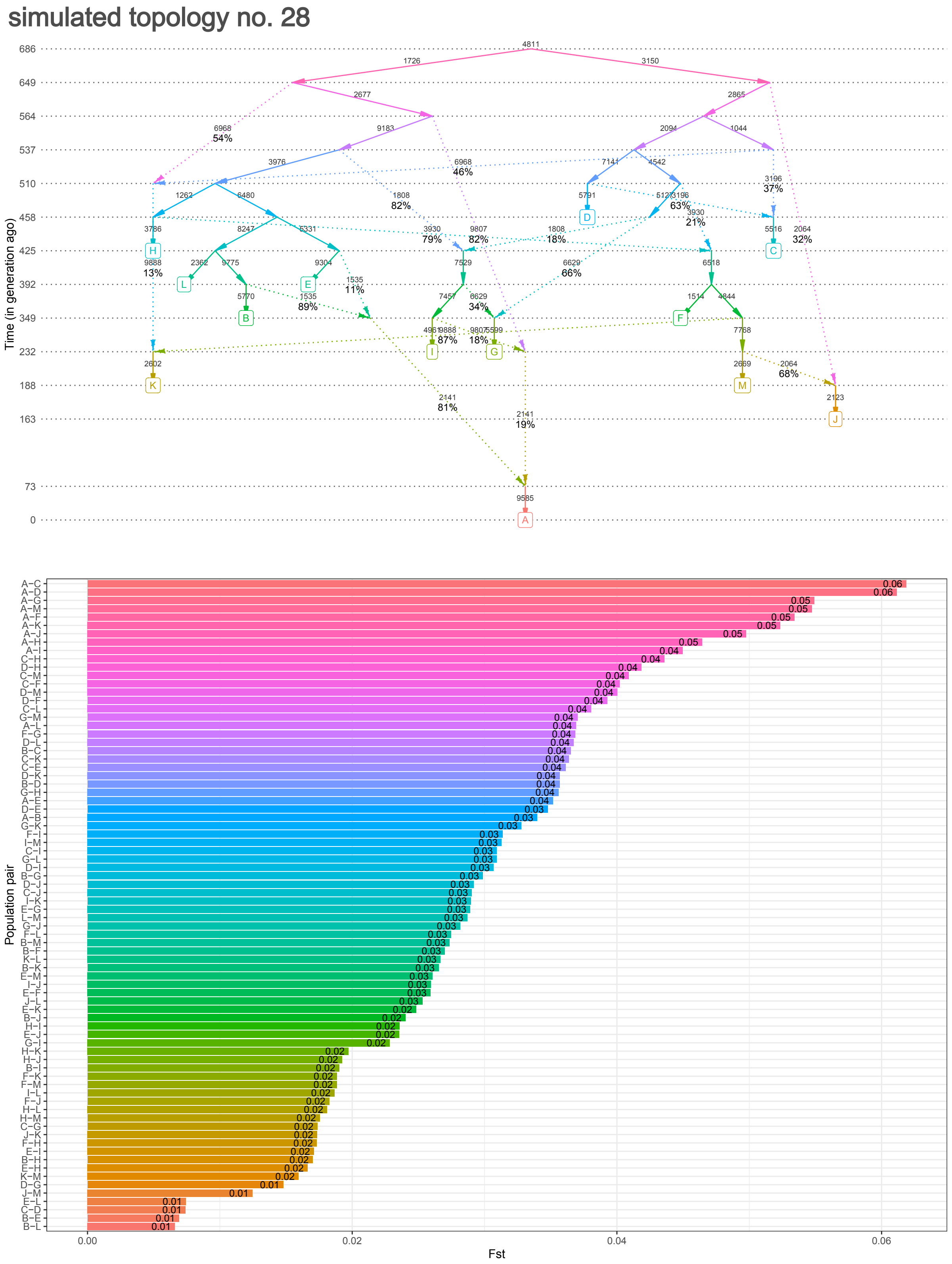
simulated topology no. 28
Time (in generation ago)
686
649
564
537
510
458
425
392
349
232
188
163
73
0
A
B
C
D
E
F
G
H
I
J
K
L
M
Population pair
Fst
0.00
0.02
0.04
0.06

b

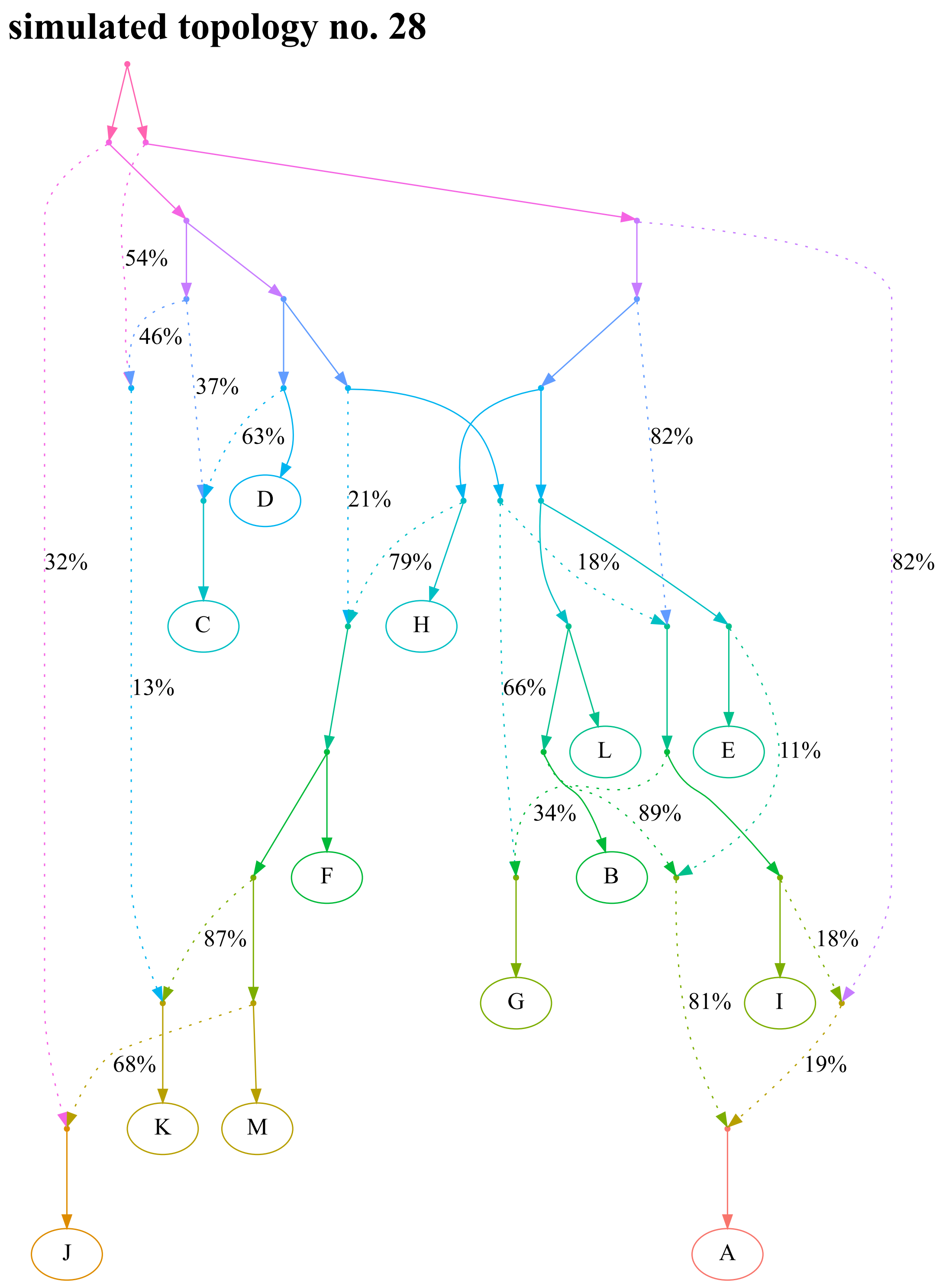
simulated topology no. 28
54%
46%
37%
63%
82%
D
21%
32%
79%
18%
82%
C
H
13%
66%
L
E
11%
34%
89%
F
B
87%
18%
G
81%
I
68%
19%
K
M
J
A

c

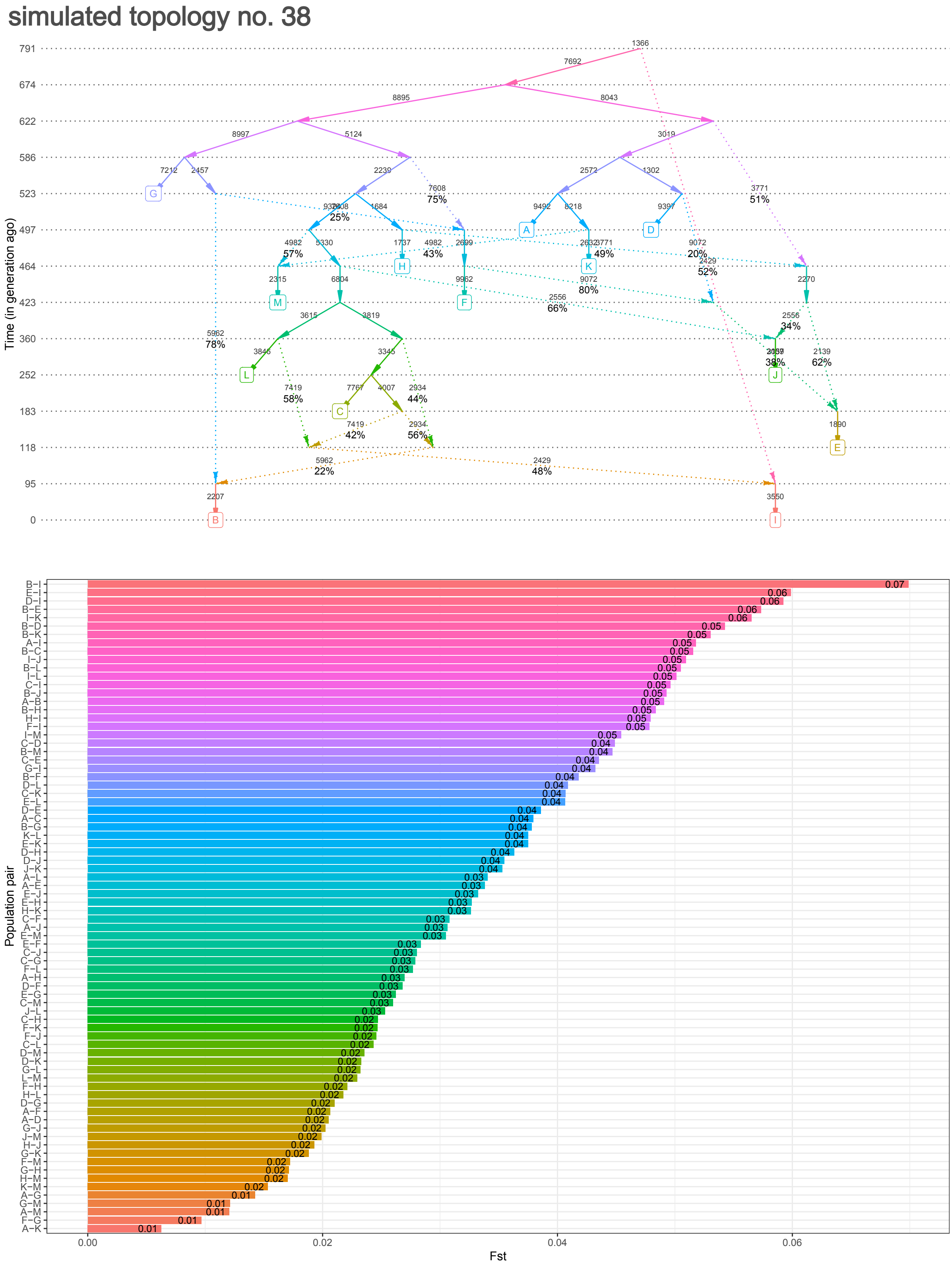

simulated topology no. 38
Time (in generation ago)
791
674
622
586
523
497
464
423
360
252
183
118
95
0
A
B
C
D
E
F
G
H
I
J
K
L
M
Population pair
Fst
0.00
0.02
0.04
0.06

d

simulated topology no. 38

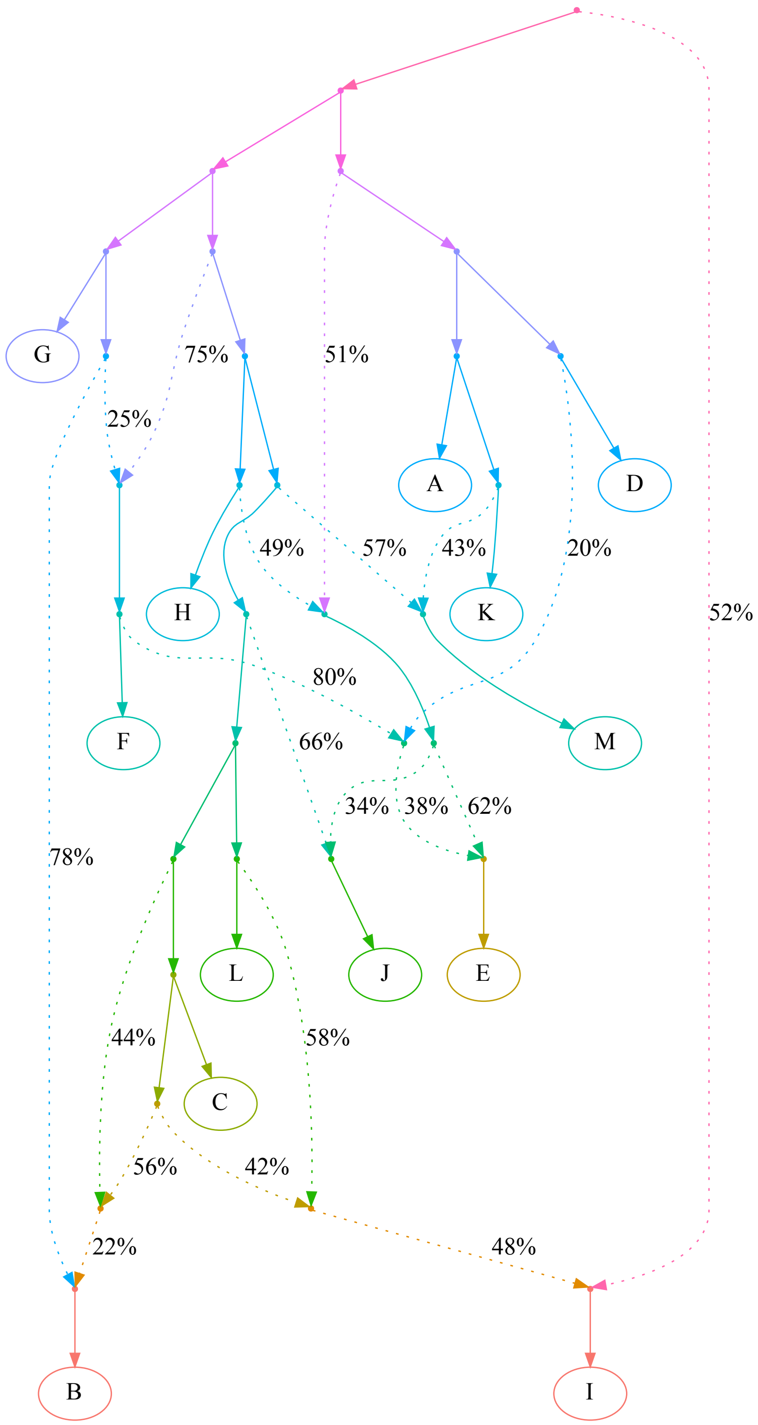

e

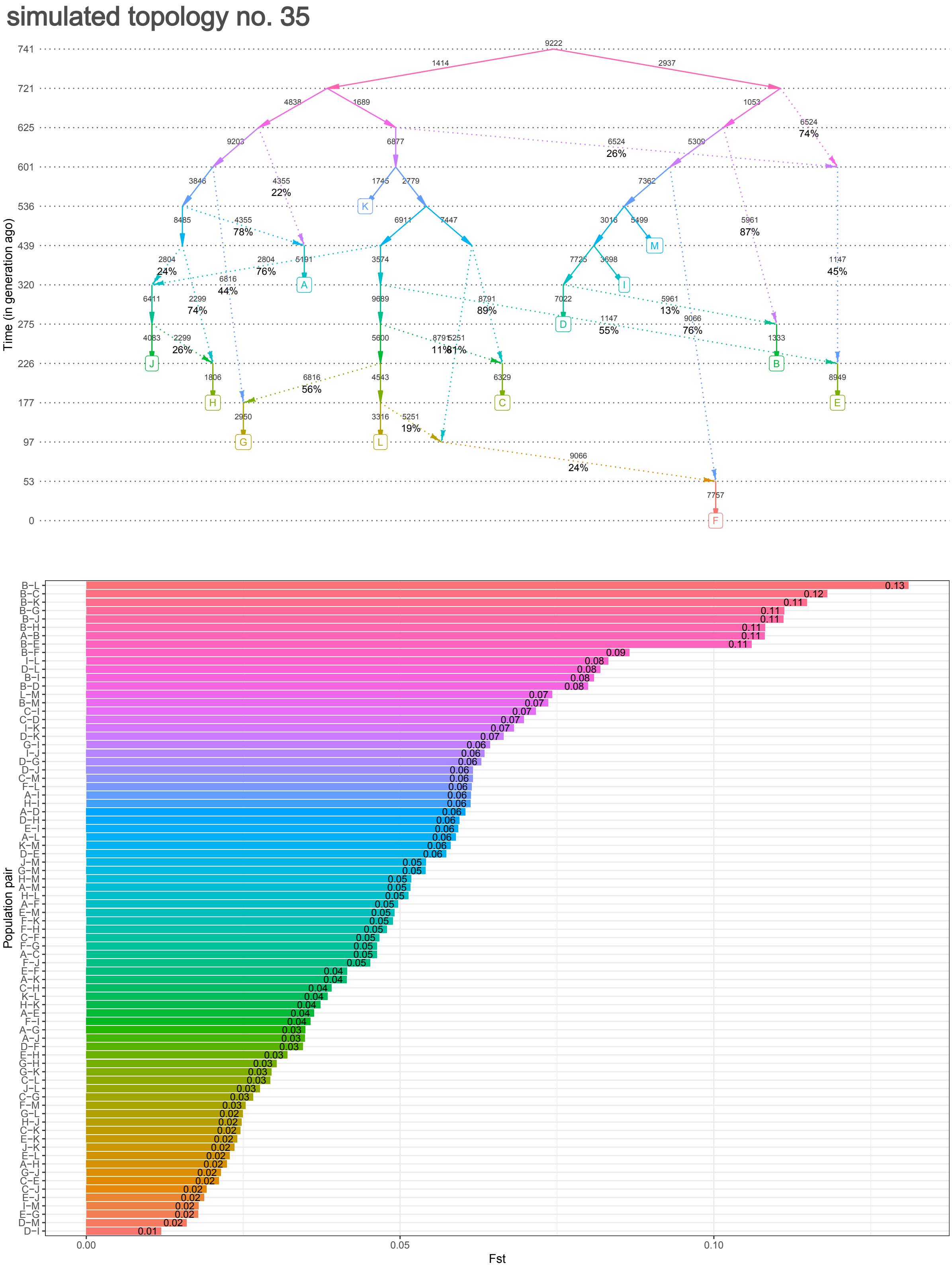
simulated topology no. 35
Time (in generation ago)
Population pair
Fst
A
B
C
D
E
F
G
H
I
J
K
L
M

f

simulated topology no. 35

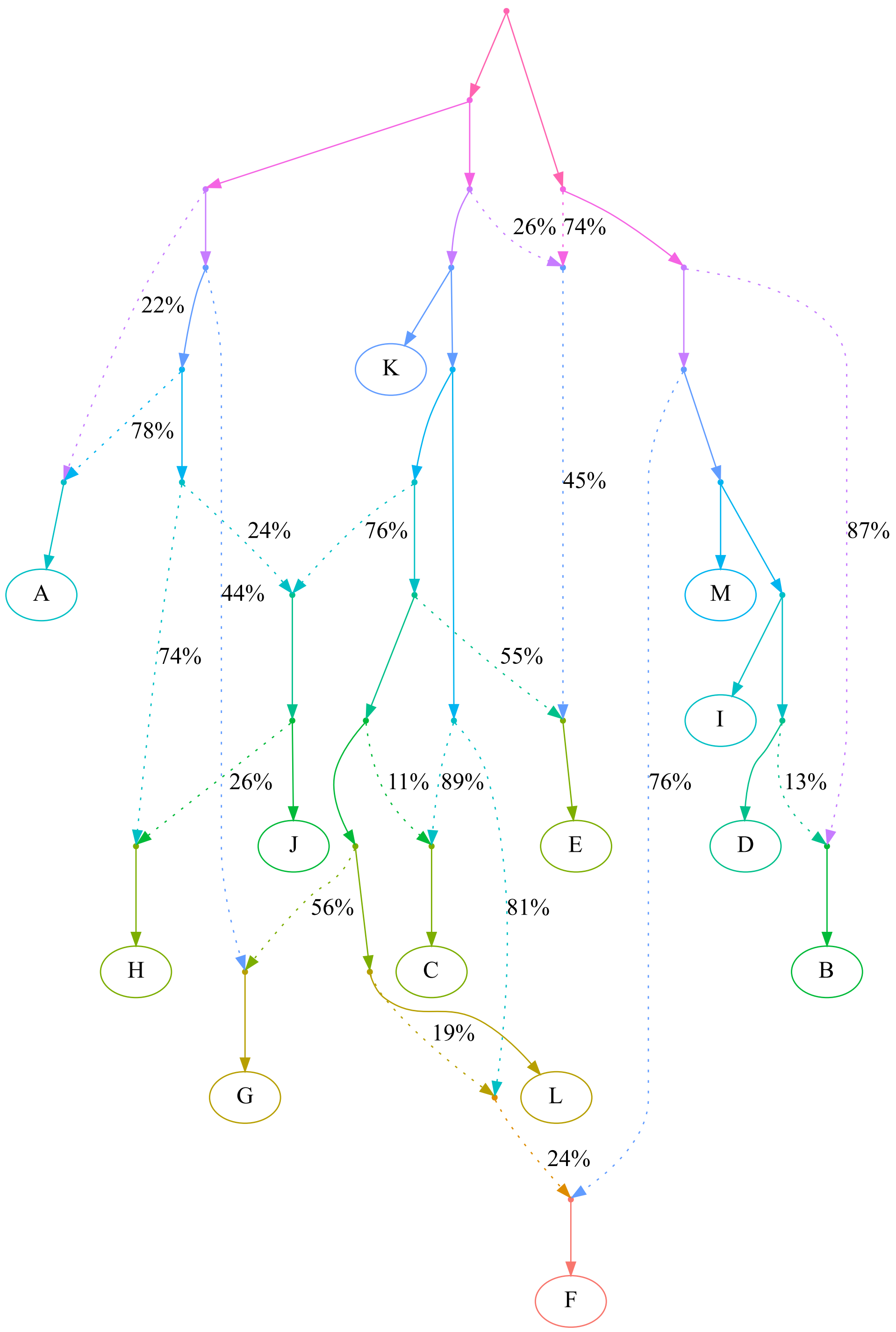

Simulated graph topologies most problematic for distal *qpAdm* protocols, i.e., those responsible for >20% of all FP 2-way *qpAdm* models in three or more sets of results. By a "set of results" we mean 40 genetic histories in the form of random admixture graph topologies of the same complexity simulated once and analysed with a certain *qpAdm* protocol. The following topologies are shown: no. 28 (panels **a, b**; problematic under 8 of 80 "protocol/simulation setup/replicate" combinations, 4 of them including the rotating protocol), no. 38 (panels **c, d**; problematic under 8 of 80 "protocol/simulation setup/replicate" combinations, 7 of them including the rotating protocol), and no. 35 (panels **e, f**; problematic under 3 of 80 "protocol/simulation setup/replicate" combinations, all of them including the rotating protocol). In all cases, F_{ST} values are shown for populations sampled at different times, simulation replicate no. 1, 300-Mbp-sized genomes, divergence times up to 800 generations, and diploid data. Time in generations is shown on the y-axis (for visual clarity, points on this axis are not spaced proportionately). Effective population sizes (in diploid individuals) are shown beside each edge. Sampled populations are labelled by letters in squares. Gene flows going from the past to the future were simulated as unsampled ghost populations. The same simulated histories are shown in a form more accessible to topological analysis, but without any additional information, in panels **b**, **d**, and **f**. The color gradients have no special meaning and are used for visual clarity only.

Figure S6.

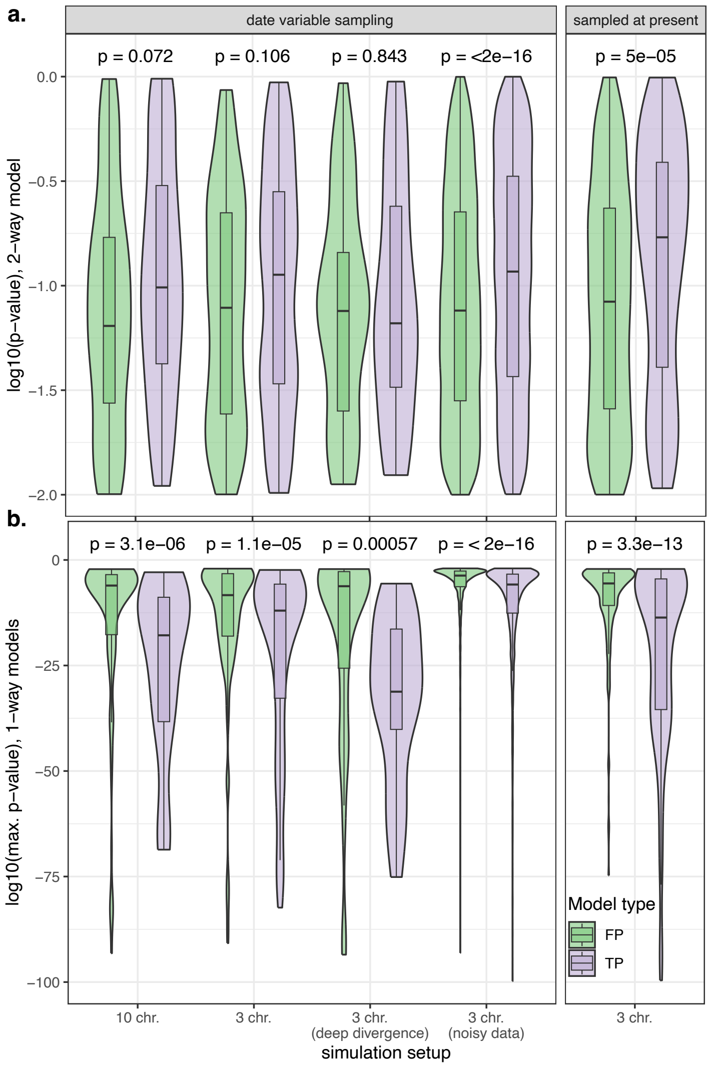

Comparing distributions of log_{10}(*p*-values) for true (TP) and false positive (FP) two-way *qpAdm* models (outcomes of the proximal rotating protocol). The distributions are summarized with boxplots and violin plots. In panel **a**, *p*-values for feasible two-way *qpAdm* models are shown. In panel **b**, the highest *p*-values in each group of three one-way models corresponding to a feasible two-way model are shown. Outliers having log_{10}(*p*-value) < -100 were removed (163 such models in total). The results are presented for five simulation setups labelled on the *x*-axis, for one simulation replicate per setup. Multiple subsampling replicates were considered in the case of low-quality data. The means were compared using the non-parametric Wilcoxon test (see *p*-values above the paired plots).
